# Analyzing Abstraction and Hierarchical Decision-Making in Absolute Identification by Information-Theoretic Bounded Rationality

**DOI:** 10.3389/fnins.2019.01230

**Published:** 2019-11-20

**Authors:** Cecilia Lindig-León, Sebastian Gottwald, Daniel A. Braun

**Affiliations:** Faculty of Engineering, Computer Science and Psychology, Institute of Neural Information Processing, Ulm University, Ulm, Germany

**Keywords:** bounded rationality, absolute identification, decision-making, abstraction, information

## Abstract

In the face of limited computational resources, bounded rational decision theory predicts that information-processing should be concentrated on actions that make a significant contribution in terms of the utility achieved. Accordingly, information-processing can be simplified by choosing stereotypic actions that lead to satisfactory performance over a range of different inputs rather than choosing a specific action for each input. Such a set of similar inputs with similar action responses would then correspond to an abstraction that can be harnessed with possibly negligible loss in utility, but with potentially considerable savings in information-processing effort. Here we test this prediction in an identification task, where human subjects were asked to estimate the roundness of ellipses varying from a straight line to a perfect circle. Crucially, when reporting their estimates, subjects could choose between three different levels of precision corresponding to three levels of abstraction in a decision-making hierarchy. To induce changes in level selection, we manipulated the information-processing resources available at the perceptual and action stages by varying the difficulty of identifying the stimulus and by enforcing different response times in the action stage. In line with theoretical predictions, we find that subjects adapt their abstraction level depending on the available resources. We compare subjects' behavior to the maximum efficiency predicated by the bounded rational decision-making model and investigate possible sources of inefficiency.

## 1. Introduction

Consider the following guessing game where you have to identify different animals drawn from a sample set of photographs. Assume that the sample set includes specimens, such as different kinds of cats (e.g., Persian, Siamese), dogs (e.g., German Shepherd, Rottweiler), snakes (e.g., Ball Python, Corn Snake), lizards (e.g., Chameleon, Leopard Gecko), frogs (e.g., Poison Dart Frog, European Tree Frog), and salamanders (e.g., Axolotl, Fire Salamander). When shown a particular exemplar, you can choose to respond with the precise name of the specimen (e.g., Rottweiler) or you can content yourself with identifying a subset corresponding to an abstract category (e.g., Dog) or even a larger subset corresponding to a more abstract super-category (e.g., Mammal, Reptile, or Amphibian). Let us assume that a precise identification is rewarded with $1, identifying a category with $0.8, and naming a super-category with $0.6. A misclassification results in no payment. Such a payment scheme naturally affords different levels of abstraction, since it allows for generic responses for various subsets of exemplars. The degree of abstraction can be measured by the amount of Shannon information contained in each subset, ultimately counting the effective number of possibilities. For example, in our sample set with 12 possibilities, identifying a specimen requires ~3.6 bits of information, identifying a category ~2.6 bits of information, and selecting a super-category distinguishes between three possibilities, i.e., ~1.6 bits of information. Clearly, in the absence of information-processing limitations—in our example the availability of at least 3.6 bits—the best response is to always identify each exemplar by its exact name.

Limitations in information-processing can have many reasons, such as limited processing time or limited memory capacity (Hick, 1952; Fitts, 1954; Miller, 1956; Slovic, 1972). These limitations can affect both perceptual and action processing, as it requires processing resources to identify an object as well as to select between different action alternatives. Also the fact that a decision-maker may not have learned yet about a particular specimen can be considered as a resource limitation, as learning about it might exceed the available response time. Abstractly, we can model limited information-processing capacity by bounding the availability of information. For instance, a decision-maker with only 2.6 bits of information available in the above example would benefit from naming categories with a reward of $0.8 instead of guessing exact names with an expected reward of only $\frac{1}{2}\times \$1=\$0.5$. Similarly, a decision-maker with even less resources (e.g., 1.6 bits) may have to abstract even more and simply distinguish between mammals, reptiles and amphibians with a reward of $0.6, rather than randomly guessing categories ($\frac{1}{2}\times \$0.8=\$0.4$) or exact object names ($\frac{1}{4}\times \$1=\$0.25$). In general, we can phrase the problem of choosing the right level of abstraction as the question of how to trade off utility and information.

**Figure 1:**
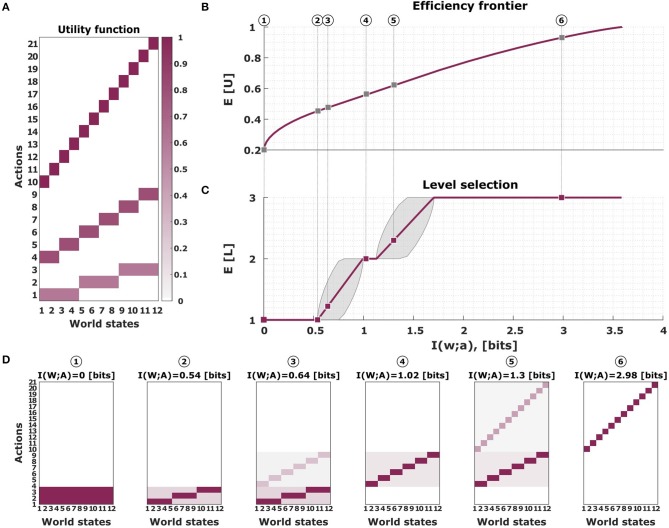
The bounded rational model of abstraction in the guessing game. In our introductory example there are twelve possible world states *w* occurring with equal probability *p*(*w*) = 1/12. The action *a* consists in choosing one of the twelve object names, one of six category names, or one of three super-category names, i.e., in total there are 21 possible actions. **(A)** The utility function of the guessing game. **(B)** The efficiency frontier for utility and information in the guessing game. The more information that is available, the higher the utility that can be achieved. **(C)** Average level selection along the efficiency frontier. The more information is available, the higher the level of concreteness. Shaded areas indicate confidence intervals given by the directed standard deviations (10). **(D)** Posterior action distributions for bounded-optimal decision-makers with varying degrees of information resources. The most limited bounded rational decision-makers have a response distribution *p*(*a*|*w*) that is confined to the lowest level (most left panel). The fully rational decision-maker chooses its actions in the top level (most right panel). The six distributions correspond to the six dots in **(B,C)**.

The trade-off between utility and information lies at the heart of information-theoretic bounded rationality (Braun et al., 2011; Ortega and Braun, 2011, 2013; Genewein et al., 2015) which encompasses a wide range of previous information-theoretic models of perception-action systems (McKelvey and Palfrey, 1995; Mckelvey and Palfrey, 1998; Mattsson and Weibull, 2002; Sims, 2003; Körding and Wolpert, 2004; Still, 2009; Todorov, 2009; Feldman and Friston, 2010; Friston, 2010; Friston et al., 2010; Tishby and Polani, 2011; Kappen et al., 2012). In these models the information-processing cost is measured by the relative Shannon information between a prior and posterior distribution corresponding to the state of knowledge before and after information-processing, respectively. The change in Shannon information can be thought to reflect the costs of information-processing, since any expenditure of computational resources improves the discriminability between alternatives. When applying this framework to our introductory example, we not only obtain an optimal efficiency frontier for utility and information, but we also obtain an optimal distribution for selecting the right level of abstraction (see Figure 1).

Here, we address the question of how efficiently human subjects can abstract in a hierarchical decision-making task, where subjects (i) select a partition with a given level of abstraction, and (ii) select the correct response inside the partition. As a behavioral assay we use an absolute identification task where subjects are offered multiple levels of precision in which they identify ellipses depending on their degree of roundness. This way we can manipulate the bounds of information-processing both by making the perceptual task more difficult by distorting the visual stimulus and by varying the processing time allowed in the action selection stage. Absolute identification tasks lend themselves for the study of limited information capacity, because of their finite number of states allowing for intuitive information measures. Accordingly, absolute identification tasks have been extensively studied in the literature in the context of information theory (Norwich, 1981; Treisman, 1985; Sims, 2016). The main novelty in our study is to manipulate the information capacity in order to investigate the effect on level selection and abstraction. In the following, we first describe the experimental paradigm and then the theoretical framework for modeling bounded rational decision-making. In the results, we measure subjects' efficiency with respect to the bounded rational optimum and investigate a number of possible sources of subjects' inefficiency.

## 2. Methods

### 2.1. Experimental Methods

#### 2.1.1. Participants

Eleven subjects, six females and five males, participated in this study. The lead author was one of the subjects (S7). All other participants provided written informed consent for participation and were remunerated with a base payment of 8 Euros per hour plus an extra incentive according to performance up to 12 Euros per hour in total. The participants were undergraduate students with normal or corrected to normal vision and no known motor deficits.

#### 2.1.2. Setup

The task was run through a graphical user interface based on Psychtoolbox in MATLAB^*TM*^ R2017a and displayed on a touch screen (Dell 27 Monitor-Touch-P2714T, 27″, 68.6 cm VIS) with maximum refresh rate of 60*Hz*. During the experiment subjects were seated in front of a desk where the touch screen was placed and tilted up at 60°.

#### 2.1.3. Trial Sequence

The experiment was divided into a training phase to facilitate subjects' adaptation and the subsequent evaluation stage. Both together required around 7 h per subject to be completed, distributed along 2 consecutive days to avoid performance loss due to fatigue.

#### 2.1.4. Experimental Design

**Figure 2:**
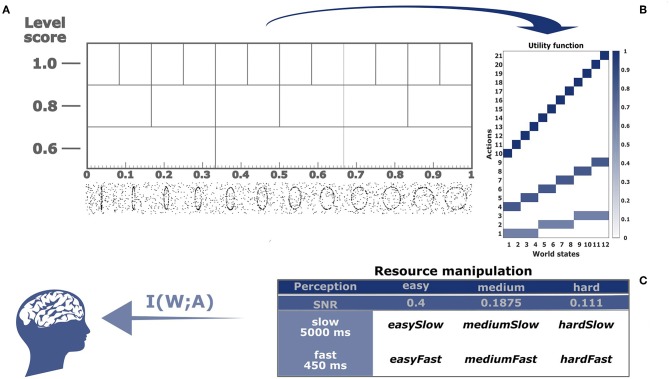
Experimental setup. **(A)** After subjects observed a noisy stimulus—see exemplary stimulus frames on the bottom—they were shown a three-stacked response panel, where each cell represents an interval of increasing roundness from left to right. At the same time, these cells are the response buttons for the subject. The buttons with highest precision and highest payoff (1 point for correct identification) are on the top of the response panel. The buttons with intermediate precision have a payoff of 0.8 points. The least precise buttons are on the bottom with a payoff of 0.6 points. **(B)** Utility function of the identification task. There are twelve world states and 3 + 6 + 12 = 21 actions, where the magnitude of the utility is color-coded and given by the number of points. **(C)** The subject is modeled as an information channel with certain capacity *I*(*W*; *A*). We manipulated the capacity in three perceptual conditions ranging from easy to hard and in two response time conditions fast and slow.

Subjects were asked to identify the roundness of animated ellipses presented to them in short video clips. They could provide their response by tapping differently sized touch screen buttons on a response panel. The panel represents intervals on a linear roundness scale with hierarchical organization reflecting three different levels of precision decreasing from top to bottom in three stacked rows (see Figure 2A). On the linear scale, roundness increased from an almost vertical line on the left to an almost perfect circle on the right. The twelve buttons on the top level of the scale would identify the roundness of the stimulus most precisely into twelve possible classes, whereas the three big buttons on the bottom of the scale would give the coarsest identification. The six intermediate sized buttons allowed for an intermediate precision for identification. Subjects were given points for each correct identification with the prospect that the points would later be converted into bonus payments. For a correct identification on the most precise level, they were given 1 point per trial, at the intermediate level 0.8 points and at the bottom level 0.6 points, and zero points for incorrect answers. This payoff scheme results in a utility function that is analogous to the utility function in our introductory example (see Figure 1A). Naturally, the three button sizes with their respective payoffs correspond to three levels of abstraction. To investigate how subjects' choice of abstraction level depends on the degree of their information boundedness, we manipulated both subjects' perceptual and motor planning capacity by introducing easy, medium and hard conditions for perceptual processing together with slow and fast conditions for motor planning (see Figure 2C).

#### 2.1.5. Stimuli

The set of world states in our task consists of twelve possible ellipses whose major axis is vertically oriented with a constant unit length that is later scaled up to ~10*cm* on the display, and whose minor axis varies for each world state ranging from an almost vertical line to an almost perfect circle. In particular we generated ellipses with vertical unit length from the following equation

$$\frac{x^{2}}{a^{2}}+\frac{y^{2}}{b^{2}}=1$$

with $b=\frac{1}{2}$ and $a=\frac{1}{24}+\frac{1}{12}\left(i-1\right)$ with *i* = 1, …, 12 so that $0<a<\frac{1}{2}$. Small values of *a* correspond to the most non-circular vertically elongated ellipses, intermediate values of *a* would be egg-shaped and large values of *a* close to 1/2 the most circular shapes. Note that we can define the roundness $w=\frac{a}{b}$ where a perfect circle requires *a* = *b* or equivalently *w* = 1.

The stimuli are generated from the twelve world states by creating video clips showing a (~12 × 12*cm*^2^) square filled with black moving dots on a gray background. In order to manipulate subjects' perceptual information-processing capacity, the majority of dots are *random dots* following random movement paths and representing noise, with a small fraction of dots (signal dots) moving along the boundary of the invisible ellipse which is always placed at the center of the touch screen. To increase the difficulty of the task, in each video frame a certain percentage of randomly selected dots—given by the replacement rate—is removed from the ellipse's path and replaced by surrounding random dots that incorporate themselves smoothly into the coherent movement. Subjects have to identify from a set of randomly moving dots the signal dots that follow a systematic movement in order to recognize the ellipse's shape. By manipulating the number of signal and random dots and the replacement rate as indicated in Table 1, we create three conditions differing in perceptual difficulty: *easy, medium*, and *hard* perception. Each video lasts 500*ms* and is produced before the start of the experiment from 30 frames. For each condition each video was only shown once.

**Table 1:** Stimulus parameters.

**Perception**	**Signal**	**Random**	**Replacement**	**Video**
**level**	**dots**	**dots**	**rate**	**duration (ms)**
Easy	100	250	35	500
Medium	75	400	40	500
Hard	50	450	40	500

Importantly, the manipulation of the perceptual difficulty does not render the stimuli ambiguous, but only makes them more difficult to process for human subjects. In all three perceptual conditions the stimuli can, in principle, be identified perfectly, as the identity of the stimulus is preserved. To demonstrate this fact, we designed an automatic recognition algorithm for ellipses with $b=\frac{1}{2}$ that computes an estimate â_*w*_ of the minor semi-axis for every point with coordinates (*x, y*) and $x\leq \frac{1}{2}$ such that

$${\hat{a}}_{w}=\sqrt{\frac{b^{2}y^{2}}{b^{2}-x^{2}}}$$

where for points that are part of the ellipse ${â}_{w}\approx \frac{1}{24}+\frac{1}{12}\left(w-1\right)$, whereas for points that are random â_*w*_ will also be random. To distinguish better between random and non-random points, the recognition algorithm compares two consecutive video frames with the idea that for pairs of non-random points the estimate â_*w*_ should be consistent, whereas for pairs of random points the estimates for â_*w*_ will differ. Pairs of points across two frames are determined simply by minimum distance. We throw away all pairs of points whose estimates for â_*w*_ are larger than a threshold value of 0.1. With the remaining points we create a histogram of all values of â_*w*_ over all frames. The stimulus *w* can then be identified by selecting the most frequent â_*w*_. As shown in Figure S1, this simple recognition algorithm achieves 100% accuracy, which means that subjects' uncertainty in identifying the stimuli arises not due to ambiguity inherent in the stimulus, but due to difficulty in perceptual processing.

#### 2.1.6. Trial Design

In the top left corner of the screen subjects could activate a start/pause button to commence each batch of trials. Each trial involves two parts: a perception stage during which the stimulus is displayed, and a subsequent action stage where subjects indicate their response on the touch screen. The perception stage starts with the presentation of a video clip according to one of the above-mentioned stimulus conditions. After 500 ms the video disappears and the response panel shown in Figure 2A is displayed. There are two response conditions. In the *slow* condition, subjects had 5 s to respond, whereas in the *fast* condition they had to select a touch screen button within 450 ms. During this deliberation time, they could freely choose both the abstraction level and the response inside the level. If the stimulus is identified correctly, then the pressed button turns green, otherwise it turns red. If subjects are too slow and exceed the response time or tap the touch screen outside the response panel, then the trial is skipped and repeated randomly at a later point in time. At any moment in time, subjects can inspect their total accumulative point score and the trial number. Subjects could pause the experiment any time by activating the pause button.

#### 2.1.7. Trial Sequence

The design of three perceptual processing conditions and two response time conditions leads to a total of six conditions: *easySlow, easyFast, mediumSlow, mediumFast, hardSlow*, and *hardFast*. Subjects were exposed to these six conditions in this exact order to allow for enough practice to master the more difficult conditions. As one might expect improvement over time due to learning, this sequence order allows us to attribute any decrement in performance observed in later trial blocks to resource constraints of the experimental condition, even though it is possible that the differences between conditions would be even larger if subjects had been trained more extensively in the beginning. Each condition consisted of 600 trials made up of six batches with 100 trials each. Accordingly, subjects experienced each world state 50 times per condition represented with different video clips to avoid over-fitting.

#### 2.1.8. Pre-training

First, subjects are allowed to experience a linear roundness scale where they can observe how their horizontal finger position is mapped continuously into the roundness of an ellipse with 0 ≤ λ ≤ 1. Second, subjects are exposed to stimuli of the easy perceptual condition with a duration of 2 × 500*ms* together with a maximum response time of 10 s. To ensure subjects gathered experience with all possible abstraction levels, in these pre-training trials level selection was enforced and marked by enhancing the color of the lines surrounding a level. The levels were selected randomly with a uniform distribution. Feedback is provided in a supervised manner across all three levels. That is, if the correct response is provided the pressed button turns green and also the correct responses in the other two levels are displayed in green. If an incorrect response is provided, the button turns red and simultaneously the correct button for each of the three levels is shown in green. The repetitions of the world-states are equally assigned over the three levels, where each one of the twelve ellipses is displayed twenty times, which makes a total of 720 trials for the whole pre-training stage. The total of trials is randomly divided into a series of six runs of 120 trials each, between which subjects can take longer pauses.

### 2.2. Theoretical Methods

#### 2.2.1. Information-Theoretic Bounded Rationality

Subjects are presented with a stimulus w∈W for which they are required to find an action a∈A whose pay-off is indicated by the utility function *U*(*w, a*). In our experiment, 0 ≤ *w* ≤ 1 represents ellipses with different degrees of roundness, and *a* corresponds to 21 possible intervals of roundness (three partitionings of [0, 1] with 3, 6, and 12 evenly sized partitions) that can be used to classify the circularity of a given ellipse. A perfectly rational decision-maker with unlimited resources would choose actions according to $a^{*}\left(w\right)=\arg\max_{a}U\left(w,a\right)$, which can be obtained as the unbounded limit case of a bounded rational decision-maker with information constraints (Ortega and Braun, 2011; Ortega et al., 2014; Genewein et al., 2015) choosing according to a distribution given by

(1) $$\begin{array}{l} p^{*}\left(a|w\right)=\underset{p\left(a|w\right)}{arg max}\left\{{\text{𝔼}}_{p\left(w,a\right)}\left[U\left(w,a\right)\right]\;|\;D_{\text{KL}}\left(p\left(a|w\right)\|p_{0}\left(a\right)\right)\leq K\right\}, \end{array}$$

where *p*_0_(*a*) represents the prior choice strategy before processing the stimulus *w*, and *p*(*a*|*w*) is the posterior policy depending on the input *w*. The decision-maker aims to maximize the expected utility 𝔼[*U*] but is limited by the upper bound *K* on the number of information bits the decision-maker can afford to deviate from the prior strategy, measured by the relative entropy $D_{\text{KL}}\left(p||q\right)=\underset{x}{\sum }p\left(x\right)\log\left(p\left(x\right)/q\left(x\right)\right)$ between prior and posterior. The constrained optimization problem in Equation (1) can be reformulated by (i) using a Lagrange multiplier β to obtain the unconstrained optimization problem

(2) $$\begin{array}{l} p^{*}\left(a|w\right)=\underset{p\left(a|w\right)}{arg max}\left\{{\text{𝔼}}_{p\left(w,a\right)}\left[U\left(w,a\right)\right]-\frac{1}{\beta }D_{\text{KL}}\left(p\left(a|w\right)\|p_{0}\left(a\right)\right)\right\}, \end{array}$$

and (ii) by choosing the optimal prior $p\left(a\right)=\underset{w}{\sum }p\left(w\right)p^{*}\left(a|w\right)$ instead of an arbitrary prior *p*_0_(*a*), which results in

(3) $$\begin{array}{l} p^{*}\left(a|w\right)=\underset{p\left(a|w\right)}{arg max}\left\{{\text{𝔼}}_{p\left(w,a\right)}\left[U\left(w,a\right)\right]-\frac{1}{\beta }I\left(W;A\right)\right\}, \end{array}$$

where *I*(*W*; *A*) = 𝔼_*p*(*w*)_[*D*_KL_(*p*(*a*|*w*)||*p*(*a*))] measures the mutual information between stimuli *w* and actions *a*. Intuitively, the mutual information measures how many bits are minimally required to process the world state *w* when determining the action *a*, given that the decision-maker wants to achieve a strategy *p*(*a*|*w*). The solution to Equation (3) can be obtained by iterating the set of equations,

(4) $$\begin{array}{l} \left\{ \begin{array}{c} p^{*}\left(a|w\right)=\frac{1}{Z\left(w\right)}p\left(a\right)e^{\beta U\left(w,a\right)}\\ p\left(a\right)=\underset{w}{\sum }p\left(w\right)p^{*}\left(a|w\right) \end{array}\right. \end{array}$$

with partition sum $Z\left(w\right)=\underset{a}{\sum }p\left(a\right)e^{\beta U\left(w,a\right)}$, which results in a Blahut-Arimoto-type algorithm well-known in rate-distortion theory (Cover and Thomas, 2006). The parameter β is determined from the bound *K* and plays the role of a resource parameter that interpolates between a decision-maker without any resources that has to choose the actions according to its prior strategy (β → 0), and a perfectly rational decision-maker with unlimited resources (β → ∞).

#### 2.2.2. The Efficiency Frontier

By traversing β from zero to infinity in Equation (4) we generate a family of bounded rational solutions $p_{\beta }^{*}\left(a|w\right)$ with expected utility ${\text{𝔼}}_{p_{\beta }^{*}\left(a|w\right)p\left(w\right)}\left[U\left(w,a\right)\right]$ and information resources $I_{\beta }\left(W;A\right)={\text{𝔼}}_{p\left(w\right)}\left[D_{\text{KL}}\left(p_{\beta }^{*}\left(a|w\right)\|p\left(a\right)\right)\right]$, resulting in a pareto-optimal efficiency frontier (see Figure 1B). The resulting rate-utility curve indicates the lowest information processing rate that is required to reach a certain level of expected utility and, conversely, the highest expected utility that is achievable given a certain rate. Decision-makers that optimally trade off expected utility against their computational resources lie exactly on the curve. Decision-makers lying above the curve are infeasible. Decision-makers lying below the curve are suboptimal considering that they could achieve a higher expected utility given the available information or, conversely, they could obtain the same benefit by investing less informational resources. In order to compare subjects' performance with respect to the efficiency frontier, we need to determine two measures from the experiment, namely the mutual information

(5) $$\begin{array}{l} I^{exp}=\underset{w}{\sum }p\left(w\right)\underset{a}{\sum }p_{\text{exp}}\left(a|w\right)\log\left(\frac{p_{\text{exp}}\left(a|w\right)}{E_{p\left(w\right)}\left[p_{\text{exp}}\left(a|w\right)\right]}\right) \end{array}$$

between stimuli *w* and subjects' responses *a*, and the average utility

(6) $$\begin{array}{l} U^{exp}={\text{𝔼}}_{p_{\text{exp}}\left(a|w\right)p\left(w\right)}\left[U\left(w,a\right)\right]. \end{array}$$

**Figure 3:**
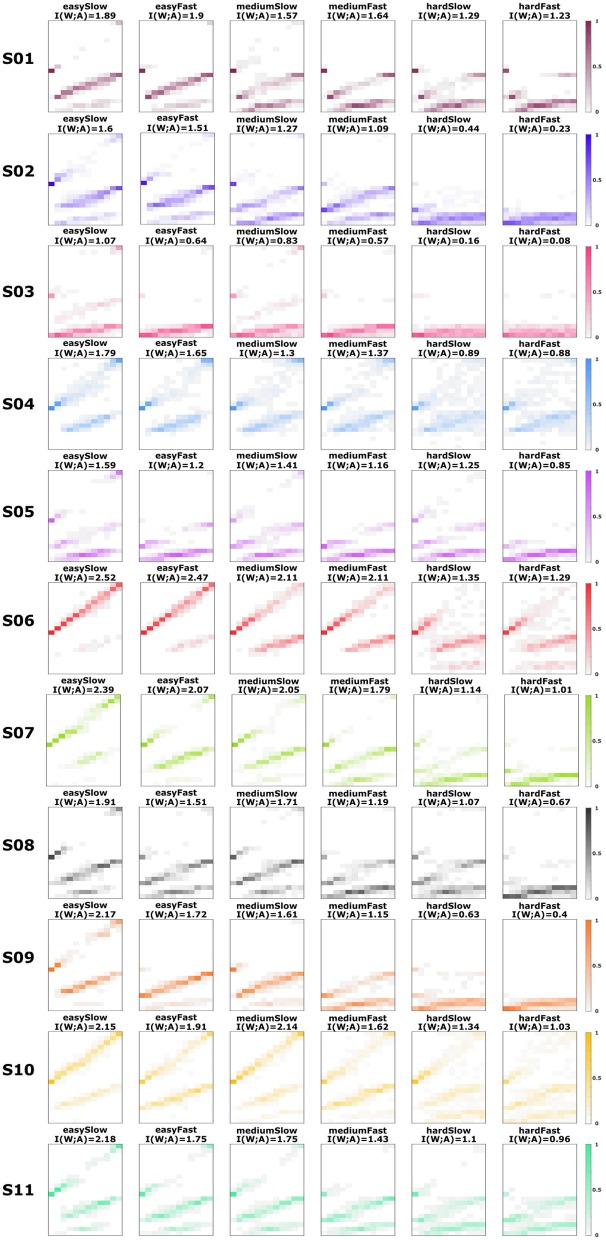
Subjects' response distributions. Estimated response distributions *p*_exp_(*a*|*w*) for all subjects across the six different experimental conditions with easy, medium and hard perception, and slow and fast reaction time. The roundness of the stimulus ellipses increases from world state 1 to world state 12. The actions are organized in three levels of abstraction: low (1–3), medium (4–9), and high (10–21).

Both quantities can be determined by estimating subjects' response probabilities *p*_exp_(*a*|*w*) for each stimulus *w*. In our scenario with discrete variables, these estimates are simply given by the empirical distributions. Consequently, subjects' performance can be represented by the tuple {*I*^*exp*^, *U*^*exp*^} in the utility-information-plane, as shown in Figure 3. Additionally, we can quantify subjects' efficiencies by

(7) $$\begin{array}{l} \epsilon =\frac{U^{exp}-U^{min}}{U^{max}-U^{min}}\leq 1, \end{array}$$

where *U*^*min*^ is the highest feasible theoretical utility in the absence of computational resources, and *U*^*max*^ indicates the maximal theoretical utility of a channel whose information processing rate is equal to *I*^*exp*^.

#### 2.2.3. Abstraction and Level Selection

The optimization problem in Equation (3) penalizes computational complexity in terms of the mutual information between actions and stimuli. An action that is exclusively selected for a particular stimulus, and that is not chosen under other circumstances, is expensive in terms of mutual information. One way to reduce informational costs while optimizing the expected utility consists in selecting an action that yields a “good enough” expected utility for many different inputs. In other words, different world states end up being treated as if they were the same. This is the essence of abstraction (Genewein and Braun, 2013). Consequently, the decision-maker does not invest its resources in discriminating among different stimuli from a subset of world states, but rather uses the same action for the entire subset. Importantly, these abstractions are shaped by the nature of the task, which is represented by the utility function *U*(*w, a*). The different levels of abstraction become apparent when formulating the decision problem as an equivalent two-step decision, where the action *a* is conceived as a tuple (*l, a*_*l*_). The high-level decision with distribution *p*(*l*|*w*) determines the abstraction level *l* which corresponds to choosing a partition with a particular granularity and distribution *p*(*a*_*l*_|*l, w*). For example at the lowest level *l* = 1, we would have the choice of *a*_1_ ∈ {Mammal, Reptile, Amphibian}. At the intermediate level *l* = 2, we would choose *a*_2_ ∈ {Dog, Cat, Snake, Lizard, Frog, Salamander}. And at the highest resolution *l* = 3, we would choose one of the twelve specimen, e.g., *a*_3_ = Rottweiler. Since the level is part of the decision, it is treated as a random variable *L*, and thus we may drop the index *l* from the action variable, so that *A* = *a* given *L* = *l* corresponds to *a*_*l*_ = *a*, in particular *p*(*w, l, a*_*l*_) = *p*(*w, l, a*).

Mathematically, the decision-making problem (3) can be equivalently reformulated as

(8) $$\begin{array}{l} \underset{p\left(l|w\right),p\left(a|w,l\right)}{arg max}\left\{{\text{𝔼}}_{p\left(w,l,a\right)}\left[U\left(w,l,a\right)\right]-\frac{1}{\beta }I\left(W;L\right)-\frac{1}{\beta }I\left(W;A|L\right)\right\}, \end{array}$$

which trades off expected utility against computational costs of the abstraction level selection *I*(*W*; *L*), and the cost of the within-level decision *I*(*W*; *A*|*L*) for each level. The bounded-optimal solution can be obtained by iterating

$$\begin{array}{lll} p^{*}\left(a|w,l\right) & = & \frac{1}{Z\left(w,l\right)}p\left(a|l\right)e^{\beta U\left(w,l,a\right)}\\ p^{*}\left(l|w\right) & = & \frac{1}{Z\left(w\right)}p\left(l\right)e^{\beta \Delta F\left(w,l\right)}, \end{array}$$

where *p*(*a*|*l*) and *p*(*l*) are marginals of *p*(*w, l, a*) = *p*(*w*) *p*(*l*|*w*) *p*(*a*|*w, l*), *Z*(*w, l*) and *Z*(*w*) are normalization constants, and $\Delta F\left(w,l\right):=\frac{1}{\beta }\log Z\left(w,l\right)$ is given by

(9) $$\begin{array}{l} \Delta F\left(w,l\right)={\text{𝔼}}_{p^{*}\left(a|w,l\right)}\left[U\left(w,l,a\right)\right]-\frac{1}{\beta }D_{\text{KL}}\left(p^{*}\left(a|w,l\right)||p\left(a|l\right)\right). \end{array}$$

The marginal *p*(*a*|*l*) serves as a prior distribution on each level, shaping the high-level partitioning of the search space. In our example, these would be uniform distributions over the actions of a given level, because of the symmetry of the utility function inside each level. More precisely, from $U\left(w,l,a\right)=u_{l}\delta_{a,a^{*}\left(w,l\right)}$, where *u*_*l*_ ∈ {0.6, 0.8, 1.0}, *N*_*l*_ ∈ {3, 6, 12} and a^*^(*w, l*) denotes the unique action with non-zero utility for world state *w* at level *l* as shown in Figure 1A, it follows that in our example the high-level utility Δ*F*(*w, l*) is independent of the world state. In fact,

$$\begin{array}{lll} \Delta F\left(w,l\right) & = & \frac{1}{\beta }\log\overset{N_{l}}{\underset{a_{l}=1}{\sum }}\frac{1}{N_{l}}\exp\left(\beta \;u_{l}\delta_{a_{l},a^{*}\left(w,l\right)}\right)\\ \; & = & \frac{1}{\beta }\log\frac{1}{N_{l}}\left(e^{\beta u_{l}}+N_{l}-1\right), \end{array}$$

and accordingly the choice of the abstraction level *l* is the same for all world states and only depends on the general performance and processing cost inside each level. We can then measure the mean level of abstraction simply by $\text{𝔼}\left[L\right]=\underset{w,l}{\sum }lp\left(w\right)p\left(l|w\right)=\underset{l}{\sum }lp\left(l\right)$ as in Figure 1C. However, since the distributions *p*(*l*|*w*) are generally asymmetric, in addition to the mean $\bar{l}:=\text{𝔼}\left[L\right]$, we also leverage the directed variances

(10) $$\begin{array}{l} \begin{array}{ccc} \sigma_{+}^{2} & = & \underset{l\geq \bar{l}}{\sum }\frac{p\left(l\right)}{\sum_{l^{\prime }\geq \bar{l}}p\left(l^{\prime }\right)}{\left(l-\bar{l}\right)}^{2}\\ \sigma_{-}^{2} & = & \underset{l\leq \bar{l}}{\sum }\frac{p\left(l\right)}{\sum_{l^{\prime }\leq \bar{l}}p\left(l^{\prime }\right)}{\left(l-\bar{l}\right)}^{2} \end{array} \end{array}$$

to characterize the level transitions, as indicated by the shaded region in Figure 1C.

#### 2.2.4. Modeling Sources of Inefficiency

When we measure subjects' efficiency based on Equation (7) and find a substantial deviation from the efficiency frontier, we can ask what causes may underlie this inefficiency. In general, one could argue that there might be more specific constraints that we have not (yet) considered in the basic form of the bounded rational model—for example, the generic distributions *p*(*a*|*w*) ∈ ℙ_Ω_ we are searching could be further constrained to be of Gaussian shape *p*_θ_(*a*|*w*) with parameters θ_*w*_ = (μ_*w*_, σ_*w*_). Formally, such constraints are represented by restricting the search space in the optimization problems (1)–(3) to a permissible subset Γ, i.e.,

(11) $$\begin{array}{l} p^{*}\left(a|w\right)=\underset{p\left(a|w\right)\in \Gamma }{\text{arg max}}\;\left\{{\text{𝔼}}_{p\left(w,a\right)}\left[U\left(w,a\right)\right]\;-\;\frac{1}{\beta }D_{\text{KL}}\left(p\left(a|w\right)||p_{0}\left(a\right)\right)\right\}, \end{array}$$

where

(12) $$\begin{array}{l} \Gamma =\{p_{\theta }\left(a|w\right)\in {\mathbb{P}}_{\Omega }|\exists \theta \;p\left(a|w\right)=p_{\theta }\left(a|w\right)\}. \end{array}$$

By solving (11) in the constraint set Γ for different values of the Lagrange multiplier β and determining the corresponding expected utility 𝔼[*U*] and mutual information *I*(*W*; *A*), we obtain the efficiency frontier under the additional constraints given by the parametrization. In the case when the parameter space is one-dimensional [e.g., when θ is given by the variance σ of a single Gaussian as in (19)], the efficiency frontier can also be generated by scanning through the parameter θ itself. We can compare subjects' performance to this constrained curve that will lie beneath the unconstrained efficiency frontier. In total, Equation (11) suggests three possible sources of inefficiency that we consider in the following:

- Non-adaptive priors *p*_0_(*a*).
- Subjective utility functions *V* ≠ *U* (different from the one stipulated by the experimenter) with
  – utility distortion of the actual payoffs (e.g., risk attitude), or
  – utility that allows for neighborhood relationships (e.g., Shepard's similarity).
- Constraints Γ ⊂ ℙ_Ω_ on the shape of permissible distributions, modeling
  – irreducible perceptual or motor execution noise, or
  – parameterized decision strategies with fixed noise structure.

Note that in contrast to Equations (1)–(3), Equation (11) does in general not allow for analytical solutions.

**(i) Non-adaptive priors**. Instead of the optimal prior distribution $p\left(a\right)=\underset{w}{\sum }p\left(w\right)p\left(a|w\right)$ that is adapted to the experimental conditions, subjects could hold arbitrary non-optimal prior beliefs *p*_0_(*a*) that do not change across conditions, for example the uniform distribution

(13) $$\begin{array}{l} p_{0}\left(a\right)=\frac{1}{21}. \end{array}$$

The prior that is uniform across levels would be

(14) $$\begin{array}{l} p_{0}\left(a\right)=\left\{ \begin{array}{cc} \frac{1}{9} & \text{if }a\in \left\{1,2,3\right\},\\ \frac{1}{18} & \text{if }a\in \left\{4,\ldots ,9\right\},\\ \frac{1}{36} & \text{if }a\in \left\{10,\ldots ,21\right\}. \end{array}\right. \end{array}$$

Instead of uniform priors, subjects could of course also have arbitrary prior beliefs across levels

(15) $$\begin{array}{l} p_{0}\left(a\right)=\left\{ \begin{array}{cc} q_{1} & \text{if }a\in \left\{1,2,3\right\},\\ q_{2} & \text{if }a\in \left\{4,\ldots ,9\right\},\\ q_{3} & \text{if }a\in \left\{10,\ldots ,21\right\}, \end{array}\right. \end{array}$$

where the probabilities of the levels are related according to 3*q*_1_ + 6*q*_2_ + 12*q*_3_ = 1. When assuming a fixed prior, we can find the best-fit values of *q*_*i*_ for each subject and all conditions. Necessarily, all these priors will induce inefficiency compared to the utility-information efficiency frontier under optimal priors.

**(ii) Subjective utility**. A subjective utility *V* is often expressed as a non-linear function of the objective function *U*, for example *V*(*x*) = [*U*(*x*)]^α^. For concave utility functions, decision-makers are risk-averse, for convex utility functions they are risk-seeking. Since we only have four utility values {0, 0.6, 0.8, 1} in our experiment, we can also explore the space of all possible local distortions by replacing 0.6 with *V*_0.6_ ∈ (0, 1) and 0.8 with *V*_0.8_ ∈ (*V*_0.6_, 1). This way, the subjects can express locally different risk attitudes, while the order of the utilities is preserved and only their absolute and relative values to each other change.

More radically, the subject could have a completely different utility function than the one stipulated by the experimenter in the task. In particular, we consider blurring the utility function as a direct way of introducing neighborhood relationships between world states. This presumes that in subjects' minds it is better to have a close miss than a distant miss. A simple way to obtain a blurred utility function *V*(*w, a*) from the original utility function *U*(*w, a*) is to assume a kernel *k*(·) that describes the decay in utility when moving away from the best action for a given stimulus, such that

$$V\left(w,a\right)=k\left(a-a^{*}\left(w,a\right)\right)U\left(w,a^{*}\left(w,a\right)\right),$$

where a*(*w, a*) denotes the unique action with non-zero utility that belongs to the same level as *a*. Here, we consider two kernels that belong to the family of Shepard's similarity functions, an exponential and a Gaussian kernel, i.e.,

(16) $$\begin{array}{l} k_{1}\left(t\right)\propto \;e^{-\theta |t|}\;k_{2}\left(t\right)\propto e^{-\frac{1}{2}\frac{t^{2}}{\theta^{2}}}. \end{array}$$

Applying the same kernel parameter θ across the three levels implies that a near-miss of a button leads to the same relative reduction in utility in all levels.

**(iii) Search space constraints**. In the basic bounded rationality model, we assume that subjects can choose according to an arbitrary strategy *p*(*a*|*w*) as long as the distribution does not undercut the entropy barrier. However, the kind of strategies subjects can implement may be further restricted in shape. In particular, we may assume that strategies can be indexed by a parameter θ, for example in Gaussian strategy profiles

(17) $$\begin{array}{l} p_{\theta }\left(a|w\right)=N\left(\mu_{w},\sigma_{w}\right)\left(a\right), \end{array}$$

with θ = {(μ_*w*_, σ_*w*_)}_*w*_. Since decision noise cannot be optimally exploited in this case, such decision-makers will be inefficient. Note that, just like in (16), parameterized decision strategies may introduce neighborhood relationships.

More sophisticated parameterized models can be obtained by assuming decision-makers with internal states. We consider a decision-maker that is not fully able to identify the state of the world *w*, but is capable of forming an internal percept *x* according to a fixed transducer *p*_θ_(*x*|*w*) whose noise characteristics are parameterized by θ. To infer the world state from a particular percept, the decision-maker can apply Bayes' rule *p*_θ_(*w*|*x*) ∝ *p*_θ_(*x*|*w*)*p*(*w*) and choose the optimal action according to $a_{\theta }^{*}\left(x\right)=\arg\max_{a}V_{\theta }\left(x,a\right)$, where *V*_θ_(*x, a*) : = 𝔼_*p*_θ_(*w*|*x*)_[*U*(*w, a*)]. Over many trials, the average response of the decision-maker to a particular world state *w* is then given by

(18) $$\begin{array}{l} p_{\theta }\left(a|w\right)=\underset{x}{\sum }p_{\theta }\left(x|w\right)\delta_{a,a_{\theta }^{*}\left(x\right)}. \end{array}$$

As a possible transducer model we consider a truncated Gaussian model,

(19) $$\begin{array}{l} p_{\sigma }\left(x|w\right)=\frac{1}{Z\left(w\right)}e^{-\frac{1}{2}\frac{{\left(x-w\right)}^{2}}{\sigma^{2}}}H\left(x\right)H\left(1-x\right), \end{array}$$

where *H*(*x*) is the Heaviside function to ensure truncation at the boundaries 0 and 1. In this model, which is also known as the Thurstonian model, the internal perceptual space is a copy of the world space but the mapping between world states and their representation is explicitly noisy. Moreover, as another transducer model we consider a binomial model motivated from Bayesian inference, where the roundnesses are treated like the success probabilities of a Bernoulli experiment that is repeated *T* times, so that the internal representation *x* corresponds to the number of successes with binomial distribution

(20) pT(x|w)=(Tx)wx(1-w)T-x.

Thus, *T* plays the role of a resource parameter (see Gottwald and Braun, 2019) that controls how well the world state can be perceived. Here, the Bayesian inverse is easily computed by using the fact that the conjugate prior of a Bernoulli likelihood *p*_*T*_(*x*|*w*) is the Beta distribution. More precisely, if the prior *p*_0_(*w*) is a beta distribution with parameters *a* and *b* (e.g., *a* = *b* = 1 for a uniform prior), then the Bayesian posterior *p*(*w*|*x*) is a Beta distribution with parameters *a* + *x* and *b* + *T* − *x*. An important side effect of having perceptual noise distributions of the form (19) and (20) is the emergence of neighborhood relationships. If two world states are so close to each other that they can be confused, their expected utility will be similar.

In the case of additional execution noise, we may assume that the observed action *a* is not the intended action that we denote by *x*, but that the planned action *x* is contaminated during the execution phase of the movement according to some fixed execution noise model *p*(*a*|*x*). During motor planning such a decision-maker can only optimize the expected utility *V*(*w, x*) = 𝔼_*p*(*x*|*a*)_[*U*(*w, a*)] for the intended action $x^{*}\left(w\right)=\arg\max_{a}V\left(w,x\right)$. Over many trials the average response of the decision-maker is

$$p\left(a|w\right)=\underset{x}{\sum }p\left(a|x\right)\delta_{x,x^{*}\left(w\right)}.$$

Unlike in our previous study (Schach et al., 2018), we regard execution noise effects as negligible in the current study due to the generously sized touch screen buttons in our experiment.

## 3. Results

Our experiment is a hierarchically organized absolute identification task involving two information-processing stages, stimulus perception and action planning, where action planning again consists of two stages, choosing a level of abstraction and identifying the stimulus given that level. This way we can manipulate the information processing capacity both in the perception and action channel to determine the relationship between overall information resources and abstraction through level selection. During stimulus presentation subjects are faced with a video clip representing the world state *w*, showing randomly moving dots, some of which trace the shape of an ellipse with roundness *w* = λ ∈ (0, 1). After processing this information, subjects are required to select an action *a* that represents an interval on the roundness scale which they deem compatible with the roundness of the perceived stimulus. The action space is organized in three levels allowing subjects to choose to make decisions with more or less precision (see Figure 2A). Each level is associated with a certain number of points subjects can earn for a correct identification, which defines a utility function whose values decrease with the size of the response intervals (see Figure 2B). As the action consists in choosing both a level of abstraction and an identification inside the level, the hypothesis is that lower computational resources imply more abstraction, which means in our task subjects should prefer lower levels as task demands become more difficult. We manipulate computational resources in two ways: by corrupting the stimuli with different levels of noise (easy, medium, and hard), and by constraining the reaction time of the decision process to fast and slow responses (see Table 2 for details). The research question in our study is how close to optimal subjects choose the level of abstraction for different ranges of information resources, and to quantify the degree of sub-optimality we might encounter.

**Table 2:** Experimental conditions.

	**Perception**	**Reaction**
**Task**	**level**	**time (ms)**
easySlow	Easy	5,000
easyFast	Easy	450
mediumSlow	Medium	5,000
mediumFast	Medium	450
hardSlow	Hard	5,000
hardFast	Hard	450

We evaluate subjects' performance by their average utility 𝔼[*U*] under their experimentally recorded response distribution *p*_exp_(*a*|*w*), which indicates the probability that subjects choose action *a* when presented with world state *w*. The recorded response distributions *p*_exp_(*a*|*w*) for all subjects are shown in Figure 3, where each row belongs to one subject. The six columns correspond to the six experimental conditions with easy, medium and hard perception and slow and fast reaction time. The stimulus-response pattern is visualized with the stimulus on the abscissa and the corresponding distribution over actions on the ordinate. The roundness of the stimulus increases from left to right. The actions correspond to choosing intervals on the roundness axis, where the twelve top level actions choose the roundness most precisely, and the six medium and three bottom actions less precisely, respectively. We see from this data that there is a substantial variability across subjects regarding level selection, but that overall subjects tend to choose more low-level actions as the experimental conditions become more difficult.

**Figure 4:**
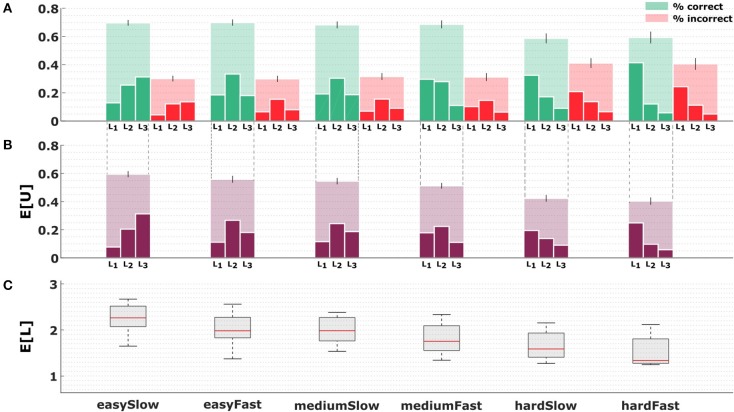
Subjects' performance depending on abstraction level across conditions. **(A)** The big opaque bars in the top row show the hit (green) and miss (red) rates for all six conditions. The dainty bars inside the big bars indicate the relative contribution of each level to the overall number of hits or misses, respectively, such that the added height of the dainty bars equals the height of the big bar. **(B)** The opaque bars in the middle row depict the average utility achieved by subjects in the six different conditions, the dainty bars show the contribution of each level to the total utility score. **(C)** The bottom row indicates the average level selected by subjects across conditions.

In Figure 4 we can see the change in level selection averaged over all subjects. The top row shows the hit and miss rates for all six conditions, where the hit rates for the easy and medium perceptual conditions range on average around 70%, and for the hard perceptual condition around 60%. These rates are achieved by subjects operating on different levels, with lower levels prevailing in the hard conditions, and higher levels dominating the easy conditions. The same picture is obtained when analyzing the average score achieved by subjects depending on the precision level, with higher precision levels having a higher utility contribution in the easier conditions. The expected utility decreases only slightly across conditions. In the bottom row, we can see the average level selected by subjects, and how this average clearly decreases as the conditions become more difficult in terms of information-processing. In the following we proceed to quantify subjects' choice efficiency with respect to a bounded rational decision-making process.

### 3.1. Subjects' Performance Compared to the Efficiency Frontier

**Figure 5:**
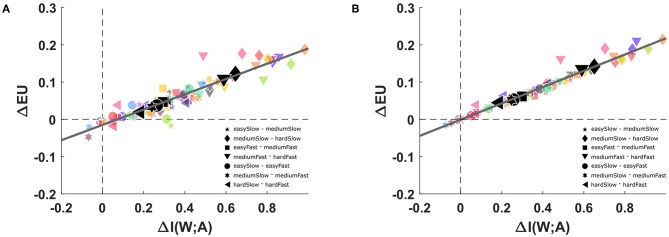
Changes in mutual information and expected utility. **(A)** Subjects' changes in expected utility and mutual information across conditions, in particular comparing slow/fast reaction time conditions, and easy/medium and medium/hard perceptual conditions. The solid line shows the linear regression fit with a correlation value of *r*^2^ = 0.78. **(B)** Changes in expected utility predicted by a bounded rational decision-making model that is afforded the same changes in mutual information as the subjects. The solid line shows the linear regression fit with a correlation value of *r*^2^ = 0.93.

In the first part of the study we compare subjects' performance against the normative performance of a bounded rational decision-maker with information constraints. To this end, we determine subjects' average utility 𝔼[*U*] and the mutual information *I*(*W*; *A*) between world states and actions. When considering the subject as an information channel, the mutual information represents the computational resources available to the subject, as more overall information-processing resources should allow for more specific stimulus-response relationships, and hence for higher mutual information and utility. The mutual information can be determined from the experimentally known distribution *p*(*w*) over stimuli and the recorded response probabilities *p*_exp_(*a*|*w*), as indicated in Equation (5). As expected, we found that mutual information in subjects' stimulus-response pattern decreases systematically when perceptual processing becomes more difficult, and similarly when reaction time is more limited (*p* < 0.01 for all comparisons between conditions, Wilcoxon ranksum test). The accompanying changes in expected utility are more subtle and only significantly decrease as perceptual processing becomes more difficult (*p* < 0.01, for all comparisons between perceptual conditions, Wilcoxon ranksum test). A summary plot of the changes in expected utility and mutual information across conditions is shown in Figure 5A. As both expected utility and mutual information increase when the task becomes easier, we find that almost all data points are in the first quadrant of Figure 5A. Moreover, the points lie on an almost perfect line, suggesting that an increase in mutual information leads to a proportionate increase in expected utility. From the results, it becomes apparent that the perceptual difficulty (especially when changing from medium to hard) has a bigger impact on available information than the reaction time limitation, notwithstanding that the combination of both constraints causes a further information reduction (compare Figure S2) for a separate accounting of changes in mutual information and expected utility due to perceptual difficulty and reaction time. In summary, we may therefore conclude that our variation of task difficulty was successful in manipulating both mutual information and expected utility.

**Figure 6:**
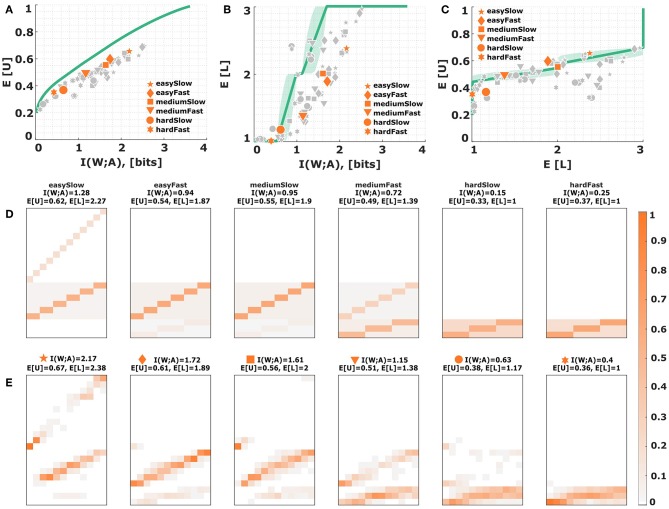
Subjects' choice efficiency. **(A)** Utility-information efficiency frontier. The solid line corresponds to the optimal efficiency of a bounded rational decision-maker and indicates the best possible expected utility achievable with a given level of mutual information between actions and world states. Each data point corresponds to a subject in a particular condition. The six emphasized data points correspond to subject S09 for who the stimulus-response distribution is shown below. **(B,C)** Level selection. The solid line in **(B)** shows the mean level selected by a bounded rational decision-maker with a given stimulus-response mutual information. The solid line in **(C)** shows the utility that it is expected to be obtained by the bounded rational actor when selecting a given level. Each data point corresponds to a subject in a particular condition, with the same subject S09 emphasized. The shaded region indicates the confidence interval given by the directed standard deviations (10). **(D)** Theoretical stimulus-response distributions for different points along the efficiency frontier closest to the data points of subject S09. **(E)** Stimulus-response pattern of subject S09 for comparison against the theoretical distributions. The fits of the remaining subjects can be seen in the Figures S8B–S17B.

The main result comparing the absolute values of mutual information and expected utility to the bounded rational optimum are presented in Figure 6 for all subjects. The solid line in Figure 6A depicts the efficiency frontier of the bounded rational decision-maker that achieves the maximum expected utility for any particular level of mutual information, or conversely, minimizes the mutual information for any given level of expected utility. The individual data points indicate subjects' performance for each experimental condition marked by different symbols. Equation (7) quantifies subjects' performance with respect to the bounded optimum and we obtain efficiencies of ~70% in our experiment (see Table S1 for details). In line with the bounded rational decision framework we find an increased use of the lower levels of the action space for the more difficult conditions with less stimulus-response mutual information. This can be seen in Figure 6B, where the solid line depicts the mean level selected by a bounded rational decision-maker. We can also quantify the average utility obtained by each subject in each condition given the average level they selected in that condition (see Figure 6C). Naturally, higher levels are predicted to be accompanied by higher average utilities, which is fulfilled by most data points (*p* < 0.01, regression slope unequal to zero). Figures 6D,E show stimulus-response distributions for different levels of information resources, where panel E shows an exemplary subject (S09) across the six conditions, and panel D depicts the theoretical stimulus-response distributions corresponding to several points along the efficiency frontier that are closest to the subject's data points in terms of the norm distance between the two distributions.

When comparing the theoretical curves and experimental data points, it appears that there is a systematic performance gap in Figures 6A,B. In particular, when comparing the stimulus-response distributions a few conspicuous differences become apparent. First, the theoretical distributions only ever spread across two adjacent levels at any one time, whereas the experimental distributions can spread across all three levels. Second, while the theoretical distribution consists of homogeneous probability blocks with clear diagonals corresponding to correct identifications, the experimental distributions are more blurry, indicating an increased tendency to respond in the neighborhood of the correct stimulus. Third, in the theoretical distributions, due to the symmetry of the utility values, there is no probability differences between stimulus-response pairs inside a particular level, whereas in the experimental data we observe that subjects prefer higher levels for stimuli close to the boundaries. This is a direct consequence of the previously reported bow effect, where extreme stimuli can be identified more reliably, which is usually explained as a boundary effect in the presence of neighborhood relationships (Kent and Lamberts, 2005; Sims, 2018). Interestingly, though, while subjects are inefficient in terms of absolute levels of performance, they are practically optimal in terms of marginal performance, that is the extra utility they achieve given an extra amount of mutual information in simpler conditions—compare Figure 5B. In our task subjects afforded on average an extra 0.22 ± 0.02 units of utility for every additional bit of information, which is practically identical to the theoretical optimum of 0.21 ± 0.01. This offset in performance hints at more specific constraints that we have not considered in the basic information-theoretic model. Therefore, in the following we consider several possible sources for subjects' inefficiency and corresponding deviations from the bounded-optimal solution (11): (i) non-optimal prior distributions, (ii) subjectively distorted utility functions reflecting neighborhood relationships and risk attitudes, and (iii) constraints on the shape of subjects' response distributions due to maladaptive noise with fixed structure in the sensorimotor system.

**Figure 7:**
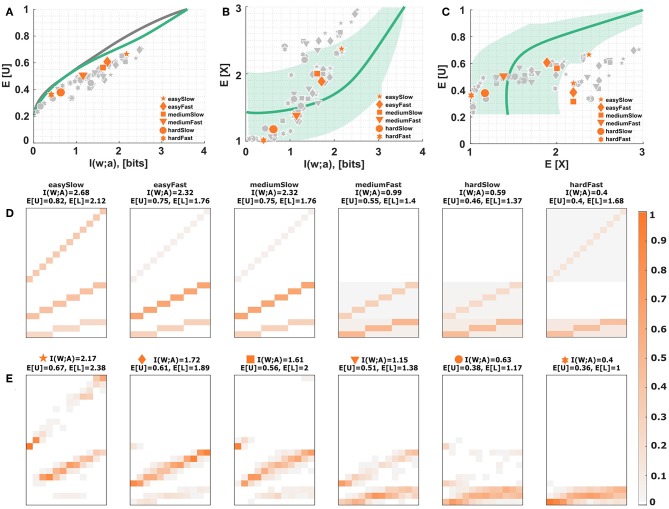
Non-optimal priors. **(A)** Utility-information efficiency frontier. The solid gray line corresponds to the optimal efficiency of a bounded rational decision-maker, the solid green line shows the efficiency frontier with the fixed prior of Equation (15). The six emphasized data points correspond to subject S09 for who the stimulus-response distribution is shown below. **(B,C)** Level selection. The solid line in **(B)** shows the mean level selected by a bounded rational decision-maker with non-optimal prior from Equation (15). The solid line in **(C)** shows the utility that it is expected to be obtained by the bounded rational actor with non-optimal prior in each level. Each data point corresponds to a subject in a particular condition, with the same subject S09 emphasized. The shaded region indicates the confidence interval given by the directed standard deviations (10). **(C)** Level selection. The solid line shows the mean level selected by a bounded rational decision-maker with non-optimal prior from Equation (15). Each data point corresponds to a subject in a particular condition, with the same subject S09 emphasized. The shaded region indicates confidence intervals given by the directed standard deviations (10). **(D)** Theoretical stimulus-response distributions for different points along the green efficiency frontier closest to the data points of subject S09 fitted according to Equation (15). **(E)** Stimulus-response pattern of subject S09 for comparison against the theoretical distributions. The fits of the remaining subjects can be seen in the Figures S8C–S17C.

**(i) Non-optimal priors**. For the efficiency frontier in Figure 6 it was assumed that subjects would adapt their choice prior *p*_0_(*a*) for each condition optimally to $p\left(a\right)=\sum_{w}p\left(w\right)p^{*}\left(a|w\right)$ as in Equation (3). Contrary to this assumption subjects might not adapt their choice priors optimally over the training course—for example, because of the limited number of training trials— and maintain a fixed prior throughout the experiment. As described in section 2.2.4, we test three different fixed priors *p*_0_(*a*), either giving more weight to the higher levels, or equal weights across levels, or more weight to the lower levels. The latter case is depicted in Figure 7, as it is the only prior where subjects start entirely at the bottom level in the absence of informational resources. The effects of the other two priors can be inspected in Figures S3, S4. Figure 7A shows that the efficiency frontier moves closer to the data under the sub-optimal prior, especially for data points with higher mutual information. However, Figures 7B,C show the prediction that subjects should only ever leave the first level when they perform perfectly in the lowest level both in terms of utility and required information. In reality, however, subjects leave the lower level much earlier, as would be predicted by a bounded optimal decision-maker with adaptive prior. While it is possible to simultaneously cover all three levels with the sub-optimal prior, it is not possible to exclusively cover the intermediate level (see Figures 7D,E).

**Figure 8:**
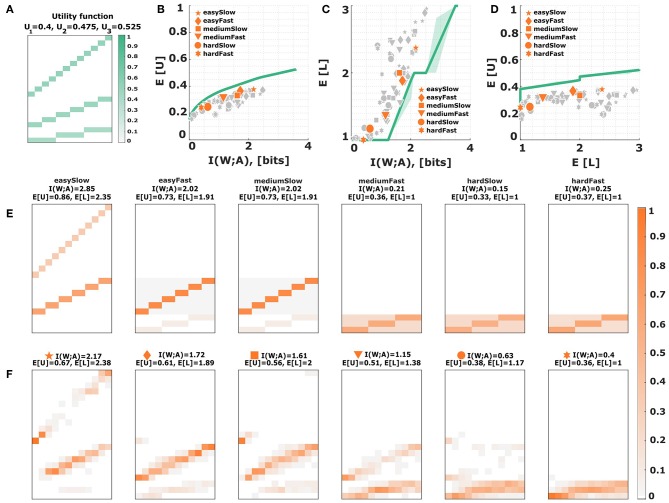
Subjective utility distortion. **(A)** Assuming a subjective distortion of the utility function changes the relative utility of each level. The depicted utility function represents a risk-seeking attitude and is chosen so as to maximize subjects' efficiency. **(B)** Efficiency frontier under utility **(A)**. Each data point corresponds to a subject in a particular condition. The six emphasized data points correspond to subject S09 for who the stimulus-response distribution is shown below. **(C,D)** Level selection. The solid line in **(C)** shows the mean level selected under a given stimulus-response mutual information. The solid line in **(D)** shows the utility that it is expected to be obtained in each level. Each data point corresponds to a subject in a particular condition, with the same subject S09 emphasized. The shaded region indicates confidence intervals given by the directed standard deviations (10). **(E)** Theoretical stimulus-response distributions for different points along the efficiency frontier closest to the data points of subject S09. **(F)** Stimulus-response pattern of subject S09 for comparison against the theoretical distributions. The fits of the remaining subjects can be seen in the Figures S8D–S17D.

**(ii) Subjective utility**. Subjects' inefficiency could also be a consequence of their risk-attitude, as higher level actions have a higher chance to fail than lower level actions. Risk-attitude can be modeled by characteristic distortions of the utility function, where concave distortions between subjective and objective utility functions represent risk-averse choice behavior and convex distortions capture risk-seeking choice behavior. To test whether such utility distortions might be able to explain the efficiency gap in our experiment, we distorted the relative utility values in the [0, 1]-interval so as to best fit the experimental response distributions. The resulting distorted utility values for a particular subject can be inspected in Figure 8A. Changing the relative utility values affects of course the efficiency frontier and the predicted level selection. In general, we found that subjects' behavior with high hitting rates is best explained with utility distortions that are close together, whereas subjects' behavior with lower hitting rates is best explained with high differences in subjective utility, such that the zero-utility of a miss can be compensated. Consequently, many subjects achieve a distorted utility that lies considerably below the theoretical optimum in each level (see Figures 8C,D as an example). While the absolute performance gap is narrowed down under a distorted utility (see Figure 8B), the subjects' average efficiency according to Equation (7) is close to 60%, and thus even lower than the efficiencies with undistorted utility. Since distorting utility values does not introduce neighborhood relationships, optimal behavior has a block structure with no preference for extreme stimuli, which does not fit subjects' behavior (see Figures 8E,F).

**Figure 9:**
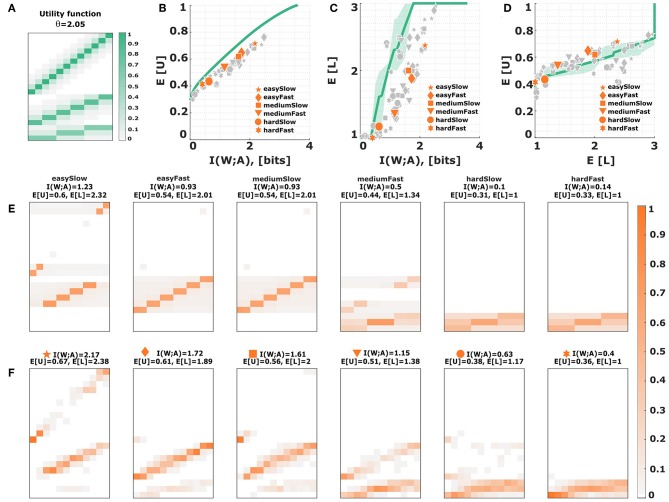
Subjective utility with neighborhood relationships. **(A)** Assuming a blurred utility function using an exponential similarity kernel of the shape *k*_1_ in Equation (16) with a decay constant of θ = 2.05 leads to non-zero off-diagonal entries. **(B)** Efficiency frontier under utility **(A)**. Each data point corresponds to a subject in a particular condition. The six emphasized data points correspond to subject S09 for who the stimulus-response distribution is shown below. **(C,D)** Level selection. The solid line in **(C)** shows the mean level selected under a given stimulus-response mutual information. The solid line in **(D)** shows the average utility achieved in each condition depending on the mean level selected. Each data point corresponds to a subject in a particular condition, with the same subject S09 emphasized. The shaded region indicates confidence intervals given by the directed standard deviations (10). **(E)** Theoretical stimulus-response distributions for different points along the efficiency frontier closest to the data points of subject S09. **(F)** Stimulus-response pattern of subject S09 for comparison against the theoretical distributions. The fits of the remaining subjects can be seen in the Figures S8F–S17F.

A slightly more radical reason why subjects' behavior might seem inefficient could always be that they optimize a completely different utility function than proposed by the experimenter. One example, is the valuation of a near-miss. The basic bounded rationality model with discrete stimuli and actions does not care whether we miss by a small or large margin, however subjects may feel that it is better to have a near-miss than a far-miss. We therefore consider blurred utility functions generated by the kernels in Equation (16), thereby creating neighborhood relationships by assigning positive utility to near misses. The blurred utility with exponentially decaying utility is depicted in Figure 9A. As can be seen in Figure 9B, the absolute distance between subjects' data points and the efficiency curve is small for the difficult conditions, but with high stimulus-response mutual information the efficiency gap remains. In the hard conditions, a blurred utility allows to efficiently populate a broad neighborhood in action space with decision noise, but when decision noise is reduced under higher rationality parameters only the maximum of the utility function matters, such that the blurred utility gives almost the same efficiency frontier than the precise utility in this regime. It is therefore not too surprising that the relative distance to the frontier given by the efficiencies in Equation (7) remains around 70% across all conditions. The difference to subjects' behavior becomes particularly apparent for level selection, as shown in Figures 9C,D. Because subjects receive utility for near-misses, there is a much stronger incentive to choose higher levels, even for the hard conditions. Since blurring the utility also introduces neighborhood relationships that produce higher expected utilities at the boundaries, this model can in principle explain the bow effect of preferring higher levels for extreme stimuli (see Figures 9E,F), but also predicts level changes for isolated stimuli a bit further away from the boundary. A similar picture is obtained if we choose a Gaussian kernel function instead of the exponential kernel (compare Figure S5).

**Figure 10:**
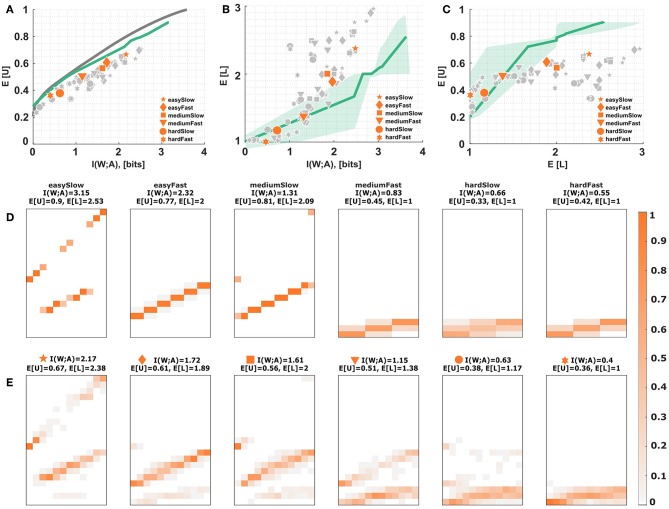
Process-dependent noise: Gaussian responses. **(A)** Assuming Gaussian decision strategies according to Equation (17) with varying standard deviation imposes a new efficiency frontier (solid green line) that lies below the bounded rational efficiency frontier (solid gray line). Each data point corresponds to a subject in a particular condition. The six emphasized data points correspond to subject S09 for who the stimulus-response distribution is shown below. **(B,C)** Level selection. The solid line in **(B)** shows the mean level selected by the Gaussian strategy profile with a given stimulus-response mutual information. The solid line in **(C)** shows the average utility achieved in each condition depending on the mean level selected. Each data point corresponds to a subject in a particular condition, with the same subject S09 emphasized. The shaded region indicates the confidence intervals given by the directed standard deviations (10). **(D)** Theoretical stimulus-response distributions for different points along the efficiency frontier closest to the data points of subject S09. **(E)** Stimulus-response pattern of subject S09 for comparison against the theoretical distributions. The fits of the remaining subjects can be seen in the Figures S8G–S17G.

**(iii) Search space constraints**. The bounded rational decision model assumes that subjects can adapt their behavior to arbitrary distributions that satisfy a mutual information constraint. However, subjects' behavioral strategies may be constrained to be of a certain shape or structural form due to neighborhood relationships in the action space, for example, which may entail a certain limitation in terms of efficiency. For instance, subjects could be limited to Gaussian strategies as proposed in Equation (17). In this case, we can find Gaussian distributions that optimize the utility information trade-off, but may differ from non-Gaussian optimal strategy distributions. Figures 10A–C show the inefficiency induced by such a model. While it looks like the restriction to Gaussian distributions might explain some of the inefficiency, the predicted level selection characteristics are far off subjects' data. The model predicts changing to a higher level only when performance is perfect, whereas subjects choose higher levels much earlier. Due to the neighborhood induced by the Gaussian response profile, the model can in principle explain the selection of high levels for boundary world states (bow effect), but wrongly predicts higher levels for center world states too (see Figures 10D,E).

Other search space constraints like perceptual noise with a fixed structure or neighborhood relationships in perceptual maps could further compromise performance, leading to a decrement both in expected utility and in mutual information. A standard model for perceptual noise is the Thurstonian model (19) that assumes internal states *x* = *w* + ϵ that represent the stimulus *w* without bias but with additive Gaussian noise ϵ. Given a perceptual noise model with a single one-dimensional parameter, such as (19) and (20), we can generate a new parametric family of response distributions with a performance curve that lies below the utility-information efficiency frontier from Figure 6A by modulating the perceptual noise parameter—for example, for the Gaussian we may scan through different levels of variance.

**Figure 11:**
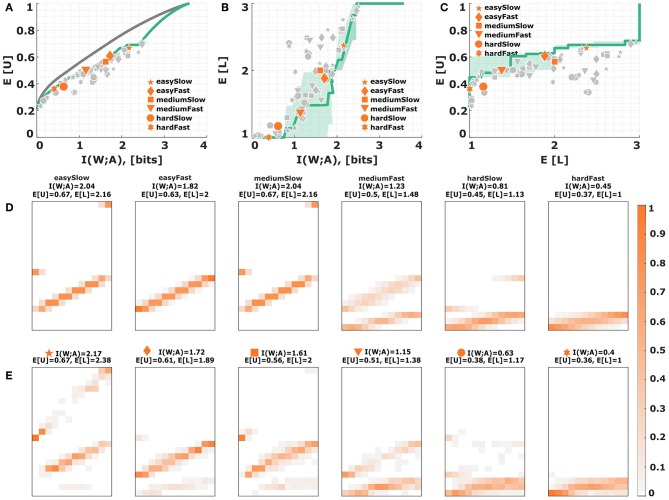
Process-dependent perceptual noise: Thurstonian model. **(A)** Assuming additive Gaussian noise for perception with different levels of variance imposes a new efficiency frontier (solid green line) that lies below the bounded rational efficiency frontier (solid gray line). Each data point corresponds to a subject in a particular condition. The six emphasized data points correspond to subject S09 for who the stimulus-response distribution is shown below. **(B,C)** Level selection. The solid line in **(B)** shows the mean level selected by a Thurstonian decision-maker with a given stimulus-response mutual information. The solid line in **(C)** shows the average utility achieved in each condition depending on the mean level selected. Each data point corresponds to a subject in a particular condition, with the same subject S09 emphasized. The shaded region indicates confidence intervals given by the directed standard deviations (10). **(D)** Theoretical stimulus-response distributions for different points along the efficiency frontier closest to the data points of subject S09. **(E)** Stimulus-response pattern of subject S09 for comparison against the theoretical distributions. The fits of the remaining subjects can be seen in the Figures S8I–S17I.

We can see subjects' behavior quantified with respect to this constrained performance curve in Figure 11A. The Thurstonian model predicts in Figure 11B that subjects should move to the upper levels only when considerably more mutual information is available than in the plain information constraint scenario of Figure 6B, which does not agree with subjects' actual level selection. When comparing the stimulus-response distributions between the Thurstonian model and subjects' data, it can be seen that neighborhood relations and blurry response profiles can be accounted for, but never across all three levels at once. Moreover, we can see that the model is able to identify extreme stimuli with higher precision, as required by the bow effect. However, this feature provides only a qualitative fit for the most extreme stimuli, and not the stimuli adjacent to the extremes.

**Figure 12:**
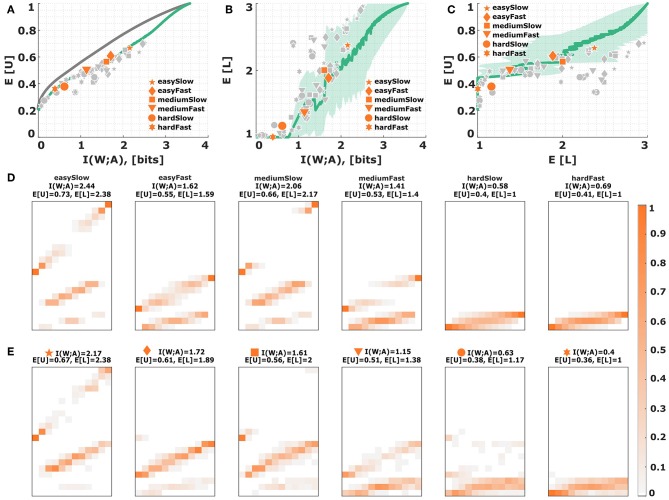
Process-dependent inference noise: Binomial model. **(A)** Assuming the unit interval as the hypothesis space in a Binomial likelihood model with different observation lengths imposes a new efficiency frontier (solid green line) that lies below the bounded rational efficiency frontier (solid gray line). Each data point corresponds to a subject in a particular condition. The six emphasized data points correspond to subject S09 for who the stimulus-response distribution is shown below. **(B,C)** Level selection. The solid line in **(B)** shows the mean level selected by a Binomial inference machine with a given stimulus-response mutual information. The solid line in **(C)** shows the average utility achieved in each condition depending on the mean level selected. Each data point corresponds to a subject in a particular condition, with the same subject S09 emphasized. The shaded region indicates confidence intervals given by the directed standard deviations (10). **(D)** Theoretical stimulus-response distributions for different points along the efficiency frontier closest to the data points of subject S09. **(E)** Stimulus-response pattern of subject S09 for comparison against the theoretical distributions. The fits of the remaining subjects can be seen in the Figures S8J–S17J.

In the Gaussian model (19) we need to artificially truncate the hypothesis space which introduces asymmetry, as roundness is only defined on the unit interval [0, 1]. We therefore also consider an inference model (20) that is slightly more abstract, but naturally constrained on the unit interval. In particular, we investigate a Binomial model, where the latent variable θ ∈ [0, 1] can be thought to reflect the unobserved roundness of an ellipse, and where the observation is given by binary strings, i.e., sequences of 0 and 1 outcomes, reflecting round and non-round cues present in the stimulus. Naturally, this is not a mechanistic model of perceptual processing, but simply phenomenological. Accordingly, the variance of the distribution is elevated for medium roundness values, and smaller for extreme roundness values, which makes it easier to identify extreme stimuli. The Bayesian belief *p*(*w*|*x*) can be expressed analytically by a Beta distribution, where the length *T* of the binary observation string determines the noise level. Just like in the Gaussian noise model, we can scan through different values of the noise parameter *T* to generate a new parameterized response distribution with a constrained efficiency frontier that lies below the utility-information efficiency frontier from Figure 6A. Subjects' behavior quantified with respect to the constrained performance curve (solid green line) of the Binomial model is depicted in Figure 12A. Subjects' level selection is better captured by the Binomial noise model as shown in Figures 12B,C. When comparing the stimulus-response distributions between model and experiment in Figures 12D,E, the model captures both neighborhood relations and blurry response profiles, even across all three levels for a single condition. The model also reproduces the bow effect, this time including both extreme stimuli and their close vicinity. Only for very large mutual information values, the model seems to break down, as subjects' data in this regime lie below the green efficiency line in Figure 12C.

### 3.2. Model Comparison

**Figure 13:**
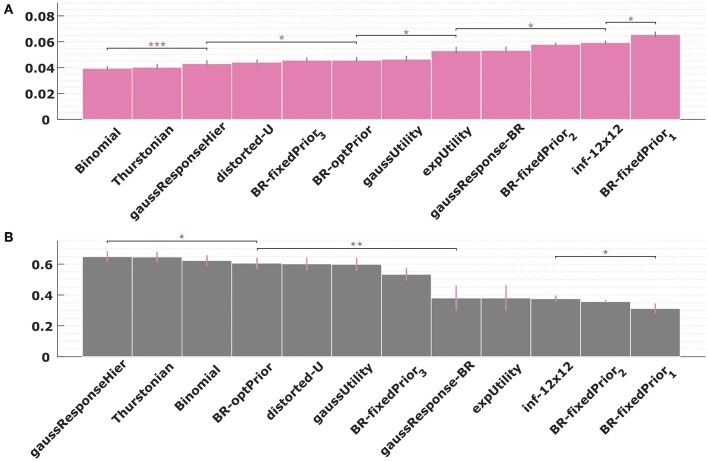
Model cross validation. **(A)** Mean absolute error between subjects' response frequencies and predicted choice probabilities. Error bars indicate standard error across subjects. Lower values indicate better predictions. **(B)** Average predicted probability of subjects choice of abstraction level. Higher values indicate better predictions. The asterisks indicate significant differences in mean (two-factor repeated measures ANOVA) between pairs of models: ***p-values < 0.001, **p < 0.01, and *p < 0.05.

In order to assess the adequacy of our models in a quantitative manner, we have performed a 10-fold cross-validation over the empirical posteriors *p*(*a*|*w*) of each experimental condition with all the models proposed. By using a cross-validation procedure we test the models' strength to predict unseen data while avoiding overfitting and/or selection bias (Cawley and Talbot, 2010). We used the same grid search over the parameters space that was used to find the best-fitting response distributions in Figures 6–12 to obtain the minimal mean absolute error between subjects' response frequencies and predicted choice probabilities. The exact parameter ranges of the grid search can be found in Table S2. Figure 13A shows the mean absolute error across all folds, conditions and subjects. The best models are the two process-dependent perceptual noise models. Comparing the models for each subject separately gives a similar picture. We found that the two perceptual models were amongst the top three fitting models for 9 out of 11 subjects (see Figures S20–S30). The best fit parameter values for the two perceptual models are heterogeneous across subjects and vary across two orders of magnitude, but are consistent within subjects across conditions (see Figures S18, S19). Finally, we performed a similar model comparison for level selection, where we applied a cross-validation considering the predicted probability of subjects' choice of abstraction level. This can be seen in Figure 13B. Again the best models from the set of models considered so far are the two perceptual noise models. However, in terms of level selection they are outperformed by the hierarchical model that we consider next.

Instead of selecting between 21 actions (*l, a*) at once, the decision-making process could also be split into two steps, where we either first select the level *l* and then the action *a* inside the level, or decide about the action *a* first and then about our confidence in this choice by selecting the level *l*. The first case can be thought of as a hierarchical decision-making problem as in Equation (9), except that both decision steps would have their own constraints (parameterized by β_1_ and β_2_, respectively), thereby optimizing

$$\text{𝔼}\left[U\right]-\frac{1}{\beta_{1}}I\left(W;L\right)-\frac{1}{\beta_{2}}I\left(W;A|L\right).$$

**Figure 14:**
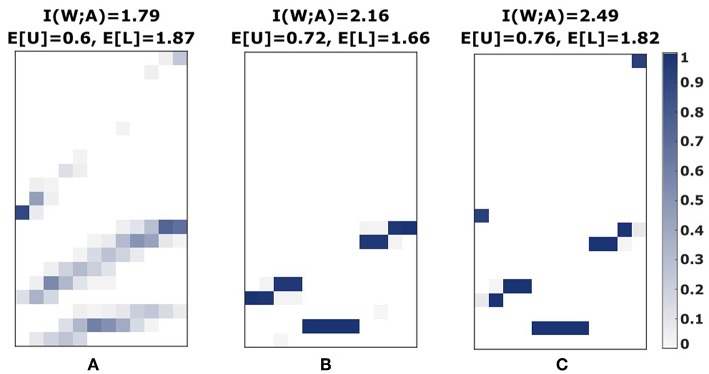
Hierarchical decision models. **(A)** Experimental stimulus-response profile of subject S08 for the fast condition with medium perceptual difficulty. **(B)** The bounded rational model with Gaussian response function spreads only over two levels. **(C)** The hierarchical bounded rational model with Gaussian response function can spread over three levels.

Crucially, such a hierarchical model allows modeling the precision of choosing a level specific for each world state, that could for example reflect the decision-maker's ability to estimate the effective utility Δ*F*(*w, l*) reflecting the trade-off between expected utility and level-specific information-processing costs. For our utility function from Figure 1A, the effective utility does not depend on the world state, so that *I*(*W*; *L*) is zero under the optimal prior *p*(*l*) and the entire constraints can be captured by one β. In case of neighborhood relationships (either introduced directly in the utility function or through process-dependent search space constraints), the hierarchical model can modulate world-state specific level selection for high β_1_ across all three levels (see Figure 14), which is impossible to achieve with a single constraint. In our experiment, however, subjects' level selection was more consistent with low and intermediate values of β_1_, as they rarely spread across all three levels, and if so, then only with high stochasticity (compare Figure S6).

## 4. Discussion

In this study we have argued that the theory of bounded rationality with information constraints provides a conceptual and normative framework to reason about abstraction and hierarchical decision-making. We have demonstrated the application of this theory to an absolute identification task where subjects could not only identify a given stimulus on a scale, but also choose the precision of that scale, which corresponds to different levels of abstraction. In order to encourage subjects to change their chosen abstraction levels, we manipulated information resources in two ways, by corrupting the stimuli and thereby aggravating stimulus identifiability and by constraining reaction time. This allowed for testing subjects' efficiency of identifying different stimuli and selecting the appropriate level of abstraction across a broad range of information conditions. We found a systematic efficiency gap in subjects' behavior across that range (~70% efficiency), which implies that with the measured amount of choice uncertainty it would in principle be possible to achieve considerably better performance (see Figure 6A). Consequently, subjects' level selection is also sub-optimal without considering further constraints on perceptual and action processes. In particular, while subjects generally obtained close-to-optimal utility in each level (see Figure 6C), they generated more information than necessary (see Figure 6B). As possible explanations for this efficiency gap we consider non-optimal prior distributions, subjective utility functions, and search space constraints in the sensorimotor system.

The assumption of bounded rationality with fixed priors is amongst the worst explanations of subjects' choice behavior and the efficiency gap—see cross validation results in Figure 13A. All models with fixed priors fail completely in explaining the adaptation of subjects' level selection across the different conditions. Similarly, allowing subjective distortions of the payoffs provided in the experiment, does not provide a satisfactory explanation of subjects' behavior. While the distorted utility model fitted subjects' responses slightly better than the undistorted model in Figure 13A, subjective utility distortion does not lead to preferences of world states inside a level, and so the block structure of predicted responses remains exactly the same as in the original undistorted model, which is in contradiction to the experimentally recorded responses showing a bow effect. A natural way to allow for different precisions for different world states, is to introduce neighborhood relationships. This can be done, for example, by assuming a blurred utility function (see Figure 9A)—whose boundary naturally leads to higher precision for extreme world states. Such a blurred utility can be regarded as an effective utility that lumps together all neighborhood constraints that are not otherwise considered in a simple bounded rationality model with just two variables. The blurred utility can explain subjects' preference of near misses, especially in the low-information range, but in the high-information range the model posteriors concentrate on the maximum utility just as in the original utility without blur. While the blurred utility leads to an increase in mutual information because responses are more concentrated within the blur, it also becomes more attractive to advance to higher levels early, as high utilities can also be achieved for near misses. These two effects compensate each other, and level selection in relation to available information remains suboptimal in the sense that choice variability is smaller and more biased than it would need to be.

Finally, we analyze process-dependent search space constraints, where the nature of the constraint depends on the particular framework under consideration, such as Markov Chain Monte Carlo planning or particular reinforcement learning models (Wang and Sandholm, 2002; Ortega et al., 2014; Viejo et al., 2015). Here we study process-dependent constraints in the context of a bounded rational analysis of information and utility by limiting the admissible set of distributions based on particular parameterizations that introduce neighborhood relationships either in the perception or action path, but with the veridical utility given by the payoffs in the experiment. In the *action path*, we assumed for example, that subjects' responses are normally distributed, centered around the correct response, and that the bounded rational decision-maker effectively has a bound on by how much the variance of these Gaussians can be reduced. The discrepancy between this model and subjects' data with respect to level selection is evident in Figures 10B,C. In the *perception path* we assumed parameterized distributions like the Gaussian and the Binomial distribution, to map inputs to an internal state and to use Bayesian inference for recovering a belief over inputs. These two models provide a good explanation of the efficiency gap in utility-information space and also correctly describe the relation between information and level selection in the low information regime, as both models introduce neighborhood relationships through their likelihood models in the internal state. However, in the high information regime, in particular with the Binomial distribution, the top level is only predicted for very high precisions. In contrast, subjects already populate the top level with considerably less precision in the experiment.

From our models, the two perceptual models are the best fitting models explaining subjects' responses in a cross-validation. Prima facie, it seems natural to classify these inference models as perfectly rational decision-making models under uncertainty. However, the perceptual uncertainty in our experiment was not a result of ambiguity inherent in the stimuli, but a result of imperfect perceptual processing. Instead of conceptualizing the decision-maker as a composite system of noisy perception and perfect action selection, where the action selection stage is trying to undo the noise induced by perception by doing inference, we may thus regard the inference process as an instance of bounded rational decision-making under information constraints, where the noise parameter effectively plays the role of a rationality parameter. This can be seen as follows. Given a parameterized distribution *p*_θ_(*a*|*w*) with a single one-dimensional parameter θ describing the noise level, there is a one-to-one mapping between θ and the rationality parameter β: we may find the optimal θ for each β value when maximizing the free energy functional, or we may simply scan through all the values of θ and determine the corresponding β, utility and information values based on θ. Consequently, what is called perfect rationality under uncertainty may generally be regarded as bounded rationality where the uncertainty is conceptualized as some kind of information constraint. So what additional insights can the bounded rationality perspective contribute? It allows us to compare behavior to the theoretical optimum with limited information independent of a particular process or the physical implementation of a decision-maker. If this optimum is not reached, it allows to quantify the efficiency gap, which then spurns research into the question what properties or constraints—expressed by the permissible subset Γ ⊂ ℙ_Ω_ in the optimization problem in Equation (11)—of a system are responsible for not reaching the optimum. In our analysis we found that subjects' level selection was close to optimal in terms of utility, but subjects also showed bias in the information produced at each level because of neighborhood relationships that are neglected in the basic model.

It has been previously criticized (Luce, 2003) that one of the major shortcomings of information theory is that information is defined over a set of unstructured elements without neighborhood relationships, which would make it unsuitable for many questions in behavioral psychology and neuroscience. However, this problem is alleviated in a bounded rational analysis, because the basic elements of choice are also assigned a utility, which can be used to introduce neighborhood relationships either directly—as in the case of the blurred utility—or indirectly—for example, by defining expected utilities on a finer grained mash. But of course, the initial definition of the choice space is critical. In our task subjects faced 12 possible world states and 21 possible actions. Under a uniform prior distribution, choosing one action therefore generates log_2_21 ≈ 4.4bits. One might argue, however, that this overstates the number of possibilities in some sense because these 21 actions are not independent and, in fact, represent only 12 possible actions with varying degree of granularity. In this interpretation, pressing a large button in the bottom is equal to pressing 4 of the 12 actions with uniform distribution, and pressing a medium sized button to pressing 2 out of 12 actions with equal probability. In this case, a random action selection would generate log_2_12bits for a small button and log_2_3bits in case of a large button. We could then pose a new optimization problem, where the decision-maker maximizes expected utility over the space of probability distributions over 21 actions, but where the information constraint is determined as if there were only 12 actions. However, the efficiency gap widens under this assumption which results in a decrement in the cross validation performance compared to the original information cost, both in action and level selection (see Figure S7).

Another possible criticism is that our experimental design conflates bounded rationality with the study of confidence in decision-making (Fleming and Daw, 2017). The fact that subjects not only had to identify a stimulus, but also had to choose the precision of their choice, may be construed as subjects making a confidence judgement by selecting the level of precision. Instead of subjects' first choosing a level and then selecting an action inside this level, one could assume that subjects first identify the stimulus—which corresponds to choosing an internal action—, and then choose the level of abstraction according to their confidence. Such confidence judgements have recently been investigated in a number of psychophysical tasks (Pallier et al., 2002; Aitchison et al., 2015; Spence et al., 2016; Kennedy and Bai, 2019). Confidence, in contrast to uncertainty, is often defined as the probability that a chosen action is correct (Pouget et al., 2016). It can be shown that in many sequential decision-making tasks it is enough for the decision-maker to reason about confidence instead of a full uncertainty analysis, for example in post-decision wagers (Persaud et al., 2007; Clifford et al., 2008; Middlebrooks and Sommer, 2012; Konstantinidis and Shanks, 2014; Vo et al., 2014). However, in our task the different levels are associated with different utilities, such that an optimal decision requires to consider the probability for each level, that is a simple confidence judgement would not be suitable. Some of the effects reported in the confidence literature may nevertheless be relevant for our study, for example the Dunning-Kruger effect (Kruger and Dunning, 1999; Dunning, 2011), where inexperienced subjects with a bad performance tend to be unaware of their incompetence and be overly confident. Such subjects might choose inadequately high levels in our task. Moreover, confidence misjudgments may also be influenced by continued processing or new evidence (Moran et al., 2015; Yu et al., 2015).

## 5. Conclusion

Our study is part of a large body of research that has investigated absolute identification with information-theoretic means in tasks involving judgements on figures (Lacouture and Marley, 2004; Stewart et al., 2005), tones (Mori and Ward, 1995), tastes (Shepard et al., 2016), and odors (Laing, 1991; Schab and Cain, 1991). Often information limits are investigated depending on the number of discriminable objects, in particular the bow effect of enhanced discriminability at the boundaries and sequence effects from one trial to the next. For the absolute judgement of different sets of line lengths, for example, information constraints have been investigated in dependence of different loss functions for misidentification, which may be regarded as a direct application of Shannon's rate distortion theory (Rouder et al., 2004; Sims, 2016). Previous analyses based on rate distortion theory have usually focused on the concept of optimal coding in perception—how many bits does it take to encode a stimulus—, but the same argument can also be applied to action selection—how many bits does it take to generate an action from a distribution (see MacKay, 2003). As pointed out in the methods, rate distortion is mathematically equivalent to a two-step bounded rational analysis with information constraints when optimizing the action prior. The novelty in our study is therefore not the quantification of information efficiency in absolute identification, but in the hierarchical design of our experiment with multiple levels of abstractions and the application of a bounded rational decision analysis to this design.

The formation of partitions, categories and abstractions has a long history in the psychological sciences (Reznikova, 2007; Braun et al., 2010). Peter Gardenfors has proposed, for example, that concepts are formed by convex sets, with the idea that a discretized perceptual space speeds up learning (Gärdenfors, 2000). Categorical perception would be a case in point, where an individual cannot distinguish anymore between some stimuli that are close together in perceptual space, so they form a discriminative unit against other stimulus groups. In our case, subjects' ability to discriminate also depends on the stimulus, where extreme stimuli can be distinguished well and are abstracted on a higher level than equivocal stimuli that are abstracted on a lower level—in our introductory example this may correspond to easily separable items (e.g., Rottweiler) and equivocal items like a xoloitzcuintle that we may simply call dog. However, unlike categorical perception the distinction of two stimuli in action space may still exist, even if identification is barely above chance level. Moreover, in our experiment we preordained the levels of abstraction in the utility function and the design of the experiment, where in the future it may be interesting to study how abstractions develop just from a given set of choice options without pre-specifying the levels.

## Data Availability Statement

The datasets generated for this study are available on request to the corresponding author.

## Ethics Statement

The studies involving human participants were reviewed and approved by Ethics committee of Ulm University. The patients/participants provided their written informed consent to participate in this study.

## Author Contributions

CL-L, SG, and DB designed the experiment. CL-L performed the experiments and analyzed the data. CL-L and SG generated the predictions from computer simulations. SG and DB supervised the project. CL-L and DB wrote the paper.

### Conflict of Interest

The authors declare that the research was conducted in the absence of any commercial or financial relationships that could be construed as a potential conflict of interest.

## References

[B1] Aitchison L. Bang D. Bahrami B. Latham P. E. (2015). Doubly bayesian analysis of confidence in perceptual decision-making. PLoS Comput. Biol. 11:e1004519. 10.1371/journal.pcbi.1004519 26517475 PMC4627723

[B2] Braun D. A. Mehring C. Wolpert D. M. (2010). Structure learning in action. Behav. Brain Res. 206, 157–165. 10.1016/j.bbr.2009.08.031 19720086 PMC2778795

[B3] Braun D. A. Ortega P. A. Theodorou E. Schaal S. (2011). Path integral control and bounded rationality, in 2011 IEEE Symposium on Adaptive Dynamic Programming and Reinforcement Learning (ADPRL), 202–209.

[B4] Cawley G. C. Talbot N. L. C. (2010). On over-fitting in model selection and subsequent selection bias in performance evaluation. J. Mach. Learn. Res. 11, 2079–2107.

[B5] Clifford C. W. Arabzadeh E. Harris J. A. (2008). Getting technical about awareness. Trends Cogn. Sci. 12, 54–58. 10.1016/j.tics.2007.11.009 18178511

[B6] Cover T. M. Thomas J. A. (2006). Elements of Information Theory (Wiley Series in Telecommunications and Signal Processing). New York, NY: Wiley-Interscience.

[B7] Dunning D. (2011). Chapter 5 - The dunning-kruger effect: on being ignorant of one's own ignorance. Adv. Exp. Soc. Psychol. 44, 247–296. 10.1016/B978-0-12-385522-0.00005-6

[B8] Feldman H. Friston K. (2010). Attention, uncertainty, and free-energy. Front. Hum. Neurosci. 4:215. 10.3389/fnhum.2010.00215 21160551 PMC3001758

[B9] Fitts P. M. (1954). The information capacity of the human motor system in controlling the amplitude of movement. J. Exp. Psychol. 47, 381–391. 13174710

[B10] Fleming S. M. Daw N. D. (2017). Self-evaluation of decision-making: a general bayesian framework for metacognitive computation. Psychol. Rev. 124, 91–114. 10.1037/rev0000045 28004960 PMC5178868

[B11] Friston K. (2010). The free-energy principle: a unified brain theory? Nat. Rev. Neurosci. 11, 127–138. 10.1038/nrn2787 20068583

[B12] Friston K. J. Daunizeau J. Kilner J. Kiebel S. J. (2010). Action and behavior: a free-energy formulation. Biol. Cybernet. 102, 227–260. 10.1007/s00422-010-0364-z 20148260

[B13] Gärdenfors P. (2000). Conceptual Spaces: The Geometry of Thought. Cambridge, MA: MIT Press.

[B14] Genewein T. Braun D. A. (2013). Abstraction in decision-makers with limited information processing capabilities. CoRR abs/1312.4353

[B15] Genewein T. Leibfried F. Grau-Moya J. Braun D. A. (2015). Bounded rationality, abstraction, and hierarchical decision-making: An information-theoretic optimality principle. Front. Robot. AI 2:27 10.3389/frobt.2015.00027

[B16] Gottwald S. Braun D. A. (2019). Bounded rational decision-making from elementary computations that reduce uncertainty. Entropy 21:375 10.3390/e21040375 PMC7514859 33267089

[B17] Hick W. E. (1952). On the rate of gain of information. Q. J. Exp. Psychol. 4, 11–26.

[B18] Kappen H. J. Gómez V. Opper M. (2012). Optimal control as a graphical model inference problem. Mach. Learn. 87, 159–182. 10.1007/s10994-012-5278-7

[B19] Kennedy J. M. Bai J. (2019). Haptic pictures: fit judgments predict identification, recognition memory, and confidence. Perception 31, 1013–1026. 10.1068/p3259 12269583

[B20] Kent C. Lamberts K. (2005). An exemplar account of the bow and set-size effects in absolute identification. J. Exp. Psychol. Learn. Mem. Cogn. 31, 289–305. 10.1037/0278-7393.31.2.289 15755246

[B21] Konstantinidis E. Shanks D. R. (2014). Don't bet on it! wagering as a measure of awareness in decision making under uncertainty. J. Exp. Psychol. Gen. 143, 2111–2134. 10.1037/a0037977 25313949

[B22] Körding K. P. Wolpert D. M. (2004). Bayesian integration in sensorimotor learning. Nature. 427, 244–247. 10.1038/nature02169 14724638

[B23] Kruger J. Dunning D. (1999). Unskilled and unaware of it: how difficulties in recognizing one's own incompetence lead to inflated self-assessments. J. Pers. Soc. Psychol. 77, 1121–1134. 10626367 10.1037//0022-3514.77.6.1121

[B24] Lacouture Y. Marley A. A. (2004). Choice and response time processes in the identification and categorization of unidimensional stimuli. Percept. Psychophys. 66, 1206–1226. 10.3758/BF03196847 15751477

[B25] Laing D. G. (1991). Characteristics of the human sense of smell when processing odor mixtures, in The Human Sense of Smell, eds Laing D. G. Doty R. L. Breipohl W. (Berlin; Heidelberg: Springer), 241–259.

[B26] Luce R. D. (2003). Whatever happened to information theory in psychology? Rev. Gen. Psychol. 7, 183–188. 10.1037/1089-2680.7.2.183

[B27] MacKay D. J. C. (2003). Information Theory, Inference, and Learning Algorithms. New York, NY: Cambridge University Press.

[B28] Mattsson L.-G. Weibull J. (2002). Probabilistic choice and procedurally bounded rationality. Games Econ. Behav. 41, 61–78. 10.1016/S0899-8256(02)00014-3

[B29] McKelvey R. D. Palfrey T. (1995). Quantal response equilibria for normal form games. Games Econ. Behav. 10, 6–38. 10.1006/game.1995.1023

[B30] Mckelvey R. D. Palfrey T. R. (1998). Quantal response equilibria for extensive form games. Exp. Econ. 1, 9–41. 10.1023/A:1009905800005

[B31] Middlebrooks P. G. Sommer M. A. (2012). Neuronal correlates of metacognition in primate frontal cortex. Neuron 75, 517–530. 10.1016/j.neuron.2012.05.028 22884334 PMC3418516

[B32] Miller G. (1956). The magical number seven, plus or minus 2: some limits on our capacity for processing information. Psychol. Rev. 63, 81–97. 10.1037/h0043158 13310704

[B33] Moran R. Teodorescu A. R. Usher M. (2015). Post choice information integration as a causal determinant of confidence: novel data and a computational account. Cogn. Psychol. 78, 99–147. 10.1016/j.cogpsych.2015.01.002 25868113

[B34] Mori S. Ward L. (1995). Pure feedback effects in absolute identification. Percept. Psychophys. 57, 1065–1079. 10.3758/BF03205465 8532496

[B35] Norwich K. H. (1981). The magical number seven: making a “bit” of “sense”. Percept. Psychophys. 29, 409–422. 10.3758/BF03207354 7279567

[B36] Ortega P. A. Braun D. A. (2011). Information, utility and bounded rationality, in Artificial General Intelligence, eds Schmidhuber J. Thórisson K. R. Looks M. (Berlin; Heidelberg: Springer), 269–274.

[B37] Ortega P. A. Braun D. A. (2013). Thermodynamics as a theory of decision-making with information-processing. Proc. R. Soc. A abs/2012.0683.

[B38] Ortega P. A. Braun D. A. Tishby N. (2014). Monte carlo methods for exact & efficient solution of the generalized optimality equations, in Proceedings of IEEE International Conference on Robotics and Automation (Hong Kong).

[B39] Pallier G. Wilkinson R. Danthiir V. Kleitman S. Knezevic G. Stankov L.. (2002). The role of individual differences in the accuracy of confidence judgments. J. Gen. Psychol. 129, 257–299. 10.1080/00221300209602099 12224810

[B40] Persaud N. McLeod P. Cowey A. (2007). Post-decision wagering objectively measures awareness. Nat. Neurosci. 10, 257–261. 10.1038/nn1840 17237774

[B41] Pouget A. Drugowitsch J. Kepecs A. (2016). Confidence and certainty: distinct probabilistic quantities for different goals. Nat. Neurosci. 19, 366–374. 10.1038/nn.4240 26906503 PMC5378479

[B42] Reznikova Z. I. (2007). Animal Intelligence: From Individual to Social Cognition. Cambridge; New York, NY: Cambridge University Press.

[B43] Rouder J. N. Morey R. D. Cowan N. Pealtz M. (2004). Learning in a unidimensional absolute identification task. Psychon. Bull. Rev. 11, 938–944. 10.3758/bf03196725 15732707

[B44] Schab F. R. Cain W. S. (1991). Memory for odors, in The Human Sense of Smell, eds Laing D. G. Doty R. L. Breipohl W. (Berlin; Heidelberg: Springer), 217–240.

[B45] Schach S. Gottwald S. Braun D. A. (2018). Quantifying motor task performance by bounded rational decision theory. Front. Neurosci. 12:932. 10.3389/fnins.2018.00932 30618561 PMC6302104

[B46] Shepard T. G. Shavit A. Y. Veldhuizen M. G. Marks L. (2016). Contextual effects in judgments of taste intensity: No assimilation, sometimes contrast. Perception 46:301006616686099. 10.1177/0301006616686099 28024444 PMC5944861

[B47] Sims C. A. (2003). Implications of rational inattention. J. Monet. Econ. 50, 665–690. 10.1016/S0304-3932(03)00029-1

[B48] Sims C. R. (2016). Rate-distortion theory and human perception. Cognition 152, 181–198. 10.1016/j.cognition.2016.03.020 27107330

[B49] Sims C. R. (2018). Efficient coding explains the universal law of generalization in human perception. Science 360, 652–656. 10.1126/science.aaq1118 29748284

[B50] Slovic P. (1972). From shakespeare to simon: speculations–and some evidence– about man's ability to process information. Oregon Res. Inst. Res. Bull. 12, 1–19. 10.1037/e310462005-001

[B51] Spence M. L. Dux P. E. Arnold D. H. (2016). Computations underlying confidence in visual perception. J. Exp. Psychol. Hum. Percept. Perform. 42, 671–682. 10.1037/xhp0000179 26594876

[B52] Stewart N. Brown G. Chater N. (2005). Absolute identification by relative judgment. Psychol. Rev. 112, 881–911. 10.1037/0033-295X.112.4.881 16262472

[B53] Still S. (2009). Information-theoretic approach to interactive learning. EPL 85:28005 10.1209/0295-5075/85/28005

[B54] Tishby N. Polani D. (2011). Information Theory of Decisions and Actions. New York, NY: Springer, 601–636. 10.1007/978-1-4419-1452-1_19

[B55] Todorov E. (2009). Efficient computation of optimal actions. Proc. Natl Acad. Sci. U.S.A. 106, 11478–11483. 10.1073/pnas.0710743106 19574462 PMC2705278

[B56] Treisman M. (1985). The magical number seven and some other features of category scaling: Properties of a model for absolute judgment. J. Math. Psychol. 29, 175–230. 10.1016/0022-2496(85)90015-X

[B57] Viejo G. Khamassi M. Brovelli A. Girard B. (2015). Modeling choice and reaction time during arbitrary visuomotor learning through the coordination of adaptive working memory and reinforcement learning. Front. Behav. Neurosci. 9:225. 10.3389/fnbeh.2015.00225 26379518 PMC4549628

[B58] Vo V. A. Li R. Kornell N. Pouget A. Cantlon J. F. (2014). Young children bet on their numerical skills: metacognition in the numerical domain. Psychol. Sci. 25, 1712–1721. 10.1177/0956797614538458 24973137 PMC4217213

[B59] Wang X. Sandholm T. (2002). Reinforcement learning to play an optimal nash equilibrium in team markov games, in Proceedings of the 15th International Conference on Neural Information Processing Systems, NIPS'02 (Cambridge, MA: MIT Press), 1603–1610.

[B60] Yu S. Pleskac T. J. Zeigenfuse M. D. (2015). Dynamics of postdecisional processing of confidence. J. Exp. Psychol. Gen. 144, 489–510. 10.1037/xge0000062 25844627

