# Supplementary material for: Analyzing Abstraction and Hierarchical Decision-Making in Absolute Identification by Information-Theoretic Bounded Rationality
Source: Front Neurosci. 2019 Nov 20;13:1230. doi: 10.3389/fnins.2019.01230 (PMC6879553; doi:10.3389/fnins.2019.01230)
Supplement: Supplementary file 1 [file Data_Sheet_1.pdf]

![frontiers]

Supplementary Material: Analyzing abstraction and hierarchical decision-making in absolute identification by information-theoretic bounded rationality

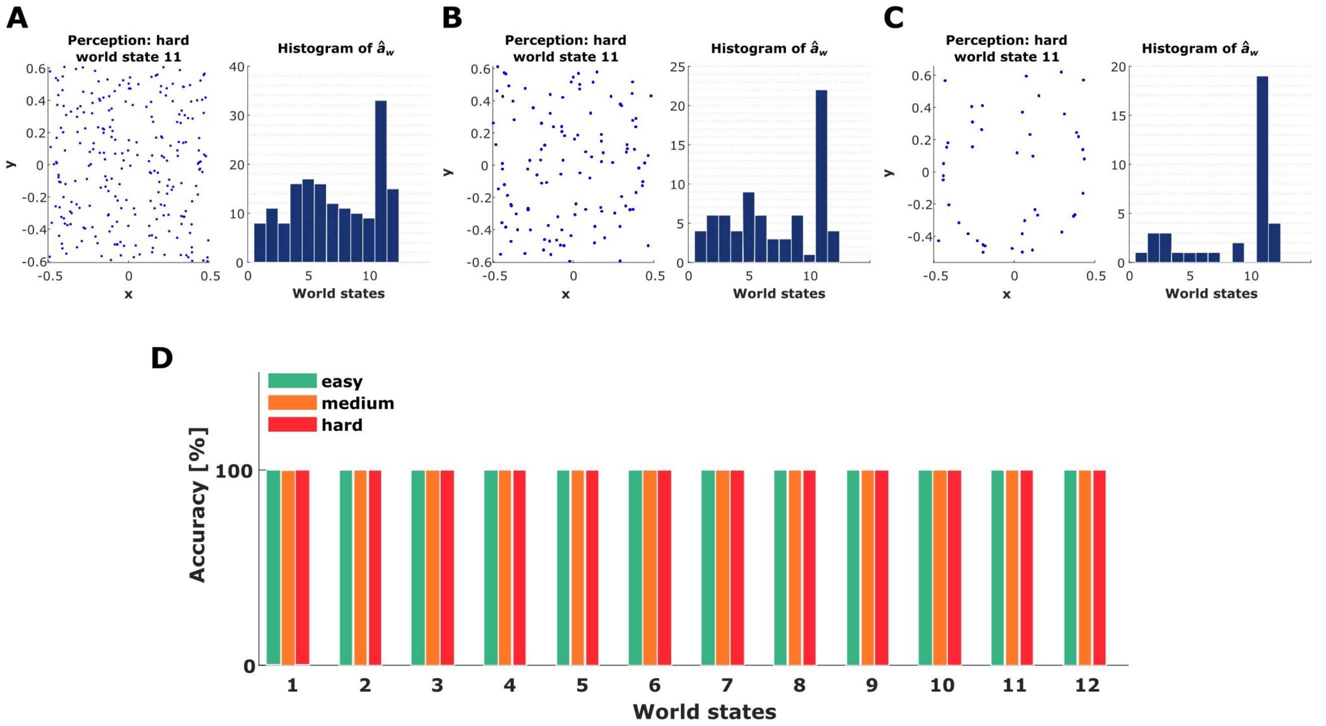

Figure S1: **Automatic recognition algorithm for ellipses.** (A) Example screen shot of a stimulus in the hard perceptual condition with world state 11, i.e. an almost circular ellipse. (B) Points of the same stimulus that can be paired to neighboring points in the next stimulus frame. (C) Points that can be paired across frames and have similar estimates $\hat{a}_w$ of the vertical semi-axis a_w. The histograms show the frequency of the estimates $\hat{a}_w$ of the points considered. (D) Accuracy of the recognition algorithm in identifying the elliptic shape.

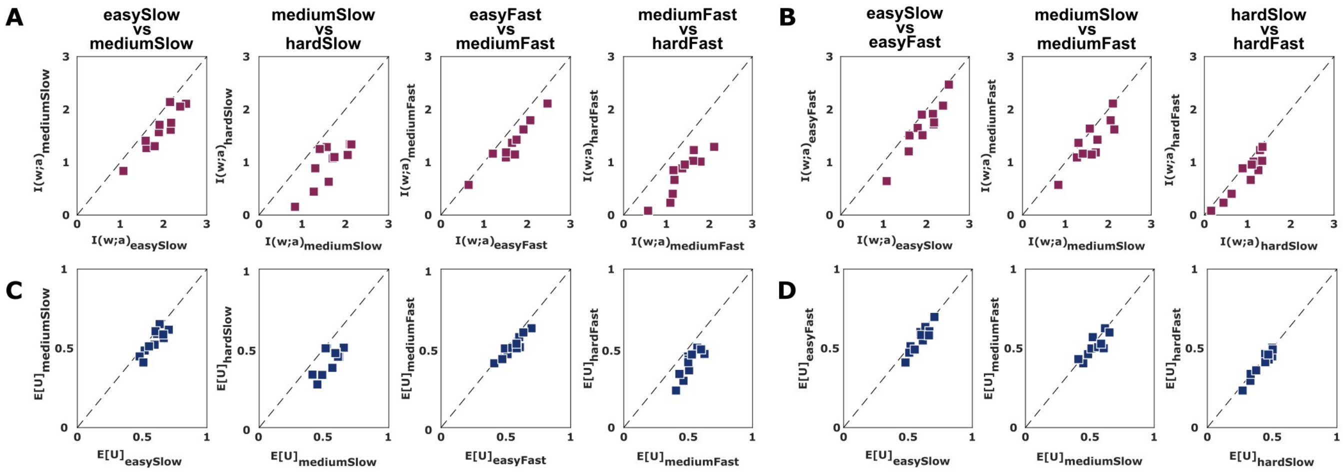

Figure S2: **Changes in mutual information and expected utility.** (A,B) Subjects' stimulus-response pattern are quantified by mutual information values between stimulus and action across experimental conditions with easy, medium and hard perception in panel A and slow and fast reaction time in panel B. (C,D) Change in subjects' expected utility across experimental conditions with easy, medium and hard perception in panel C and slow and fast reaction time in panel D. The dashed line is the diagonal that indicates no change across conditions. Each data point corresponds to a subject.

Table S1. Subjects' efficiencies

	easySlow	easyFast	mediumSlow	mediumFast	hardSlow	hardFast
S01	0.72	0.73	0.67	0.75	0.72	0.75
S02	0.66	0.67	0.68	0.70	0.57	0.57
S03	0.75	0.76	0.76	0.79	0.53	0.35
S04	0.59	0.55	0.50	0.53	0.40	0.41
S05	0.73	0.72	0.69	0.76	0.74	0.76
S06	0.76	0.76	0.71	0.72	0.57	0.63
S07	0.68	0.75	0.78	0.76	0.78	0.81
S08	0.76	0.75	0.77	0.76	0.73	0.83
S09	0.78	0.80	0.74	0.79	0.64	0.73
S10	0.68	0.70	0.69	0.61	0.57	0.58
S11	0.77	0.74	0.75	0.73	0.71	0.75

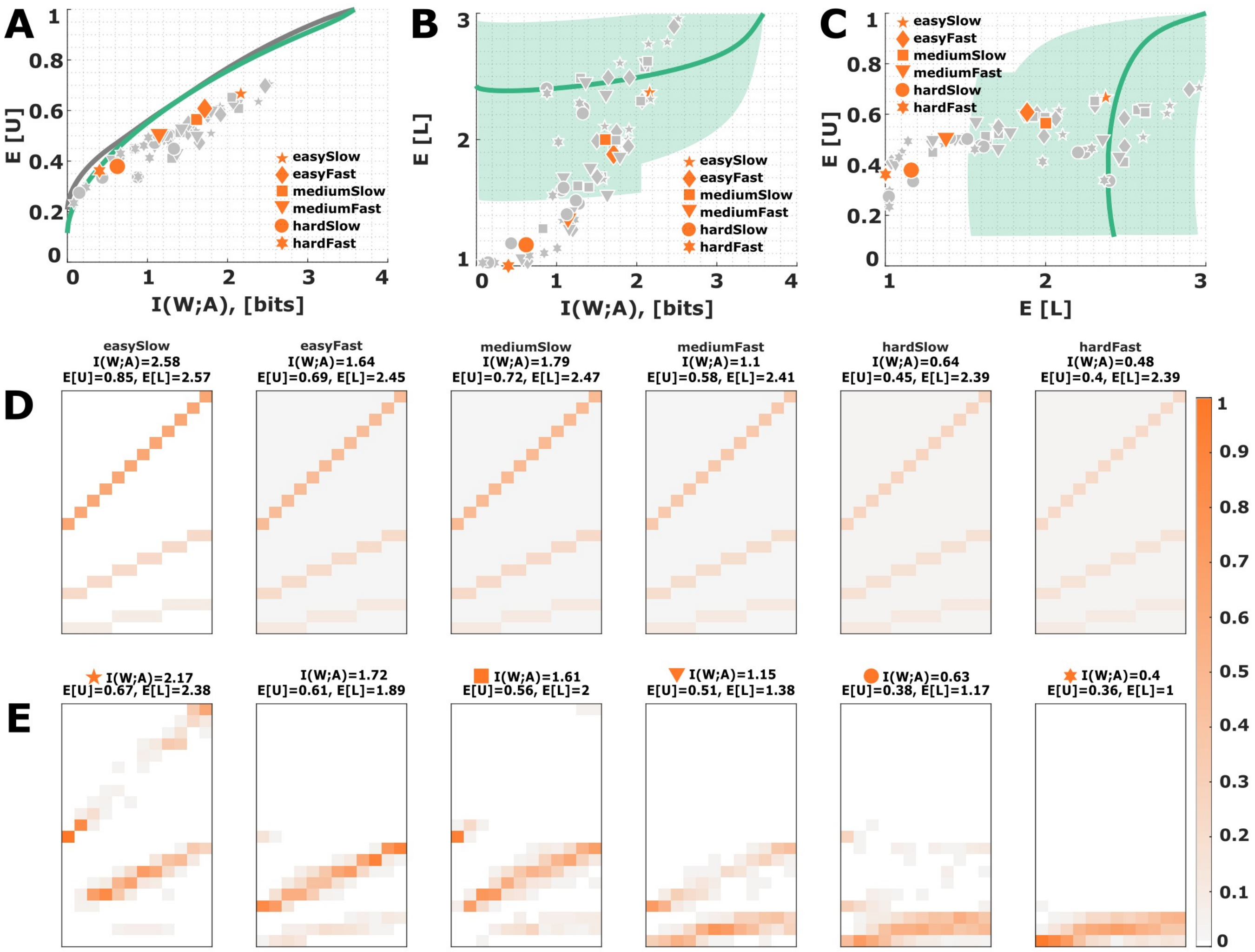

Figure S3: **Non-optimal priors.** (A) Utility-information efficiency frontier. The solid gray line corresponds to the optimal efficiency of a bounded rational decision-maker, the solid orange line shows the efficiency frontier with the fixed prior of equation 13. The six emphasized data points correspond to subject S09 for who the stimulus-response distribution is shown below. (B,C) Level selection. The solid line in panel B shows the mean level selected by a bounded rational decision-maker with non-optimal prior 13. The solid line in panel C shows the utility that it is expected to be obtained by the bounded-rational actor with non-optimal prior in each level. Each data point corresponds to a subject in a particular condition, with the same subject S09 emphasized. The shaded region indicates the signed standard deviation. (C) Level selection. The solid line shows the mean level selected by a bounded rational decision-maker with non-optimal prior 13. Each data point corresponds to a subject in a particular condition, with the same subject S09 emphasized. The shaded region indicates the signed standard deviation. (D) Theoretical stimulus-response distributions for different points along the orange efficiency frontier closest to the data points of subject S09. (E) Stimulus-response pattern of subject S09 for comparison against the theoretical distributions.

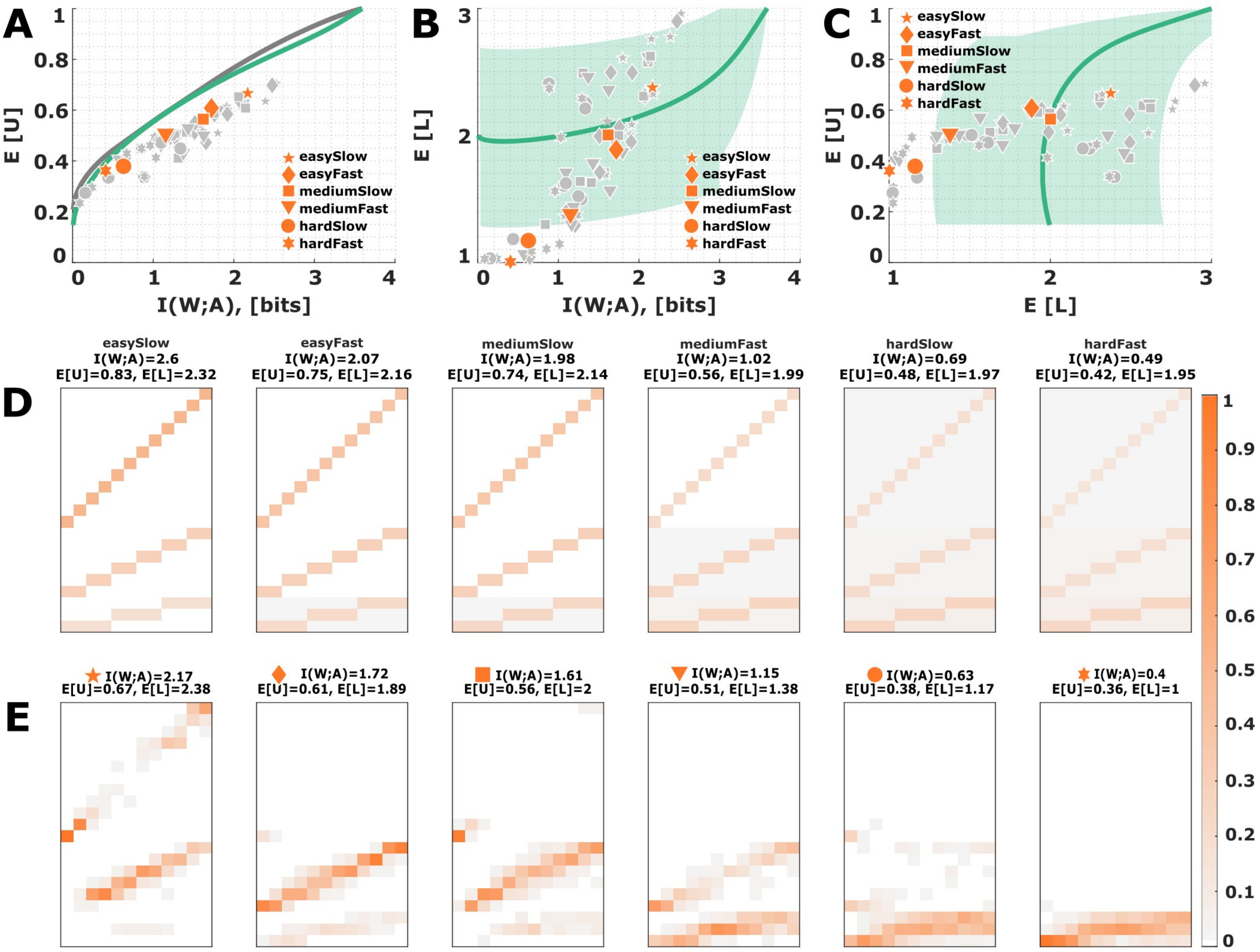

Figure S4: **Non-optimal priors.** (A) Utility-information efficiency frontier. The solid gray line corresponds to the optimal efficiency of a bounded rational decision-maker, the solid orange line shows the efficiency frontier with the fixed prior of equation 14. The six emphasized data points correspond to subject S09 for who the stimulus-response distribution is shown below. (B,C) Level selection. The solid line in panel B shows the mean level selected by a bounded rational decision-maker with non-optimal prior 14. The solid line in panel C shows the utility that it is expected to be obtained by the bounded-rational actor with non-optimal prior in each level. Each data point corresponds to a subject in a particular condition, with the same subject S09 emphasized. The shaded region indicates the signed standard deviation. (C) Level selection. The solid line shows the mean level selected by a bounded rational decision-maker with non-optimal prior 14. Each data point corresponds to a subject in a particular condition, with the same subject S09 emphasized. The shaded region indicates the signed standard deviation. (D) Theoretical stimulus-response distributions for different points along the orange efficiency frontier closest to the data points of subject S09. (E) Stimulus-response pattern of subject S09 for comparison against the theoretical distributions.

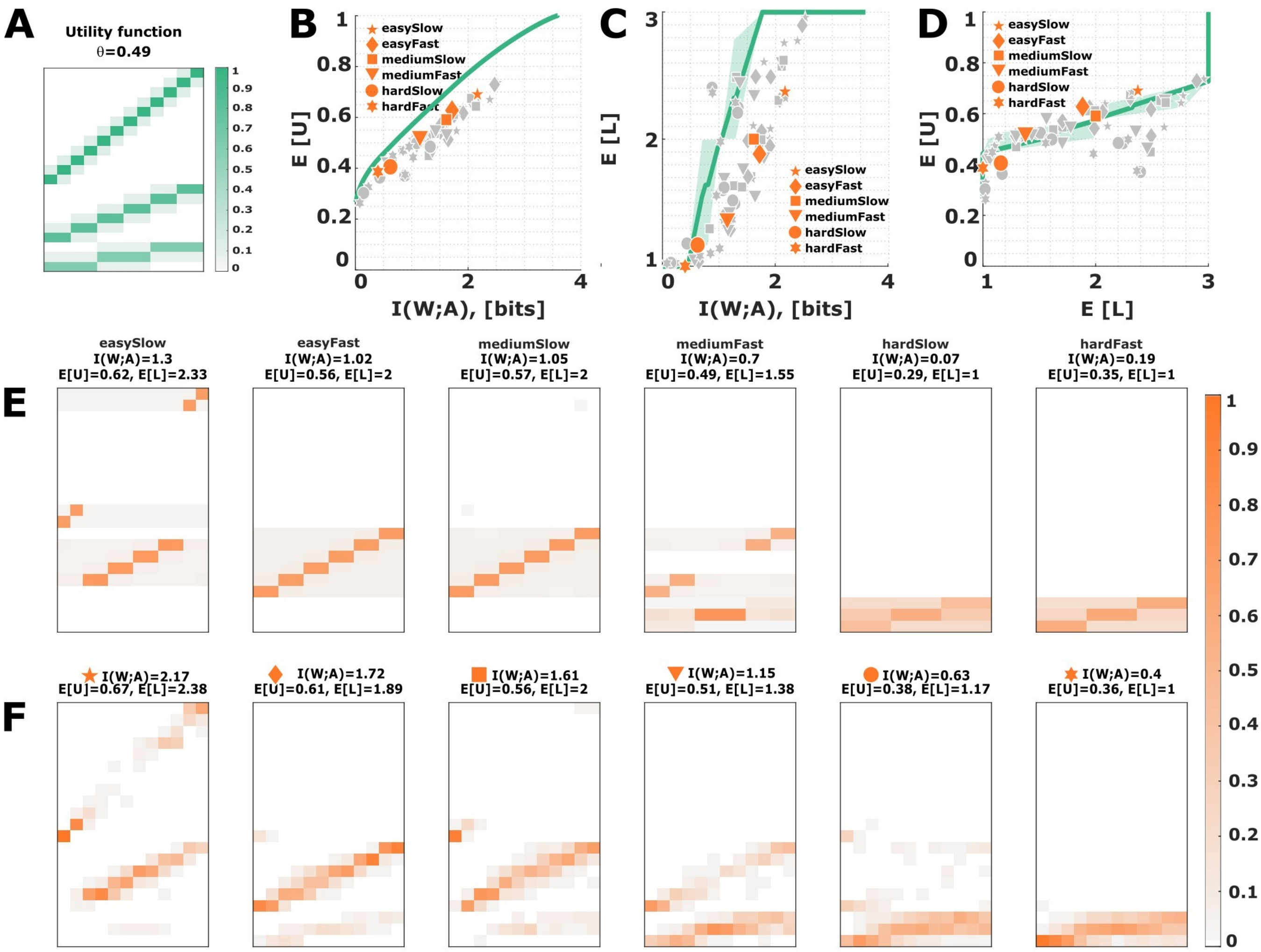

Figure S5: **Subjective utility with neighborhood relationships.** (A) Assuming a blurred utility function using a Gaussian similarity kernel according to equation 16 with a decay constant of $\theta = 2.05$ leads to non-zero off-diagonal entries. (B) Efficiency frontier under utility (A). Each data point corresponds to a subject in a particular condition. The six emphasized data points correspond to subject S09 for who the stimulus-response distribution is shown below. (C,D) Level selection. The solid line in panel C shows the mean level selected under a given stimulus-response mutual information. The solid line in panel D shows the average utility achieved in each condition depending on the mean level selected. Each data point corresponds to a subject in a particular condition, with the same subject S09 emphasized. The shaded region indicates the signed standard deviation. (E) Theoretical stimulus-response distributions for different points along the efficiency frontier closest to the data points of subject S09. (F) Stimulus-response pattern of subject S09 for comparison against the theoretical distributions.

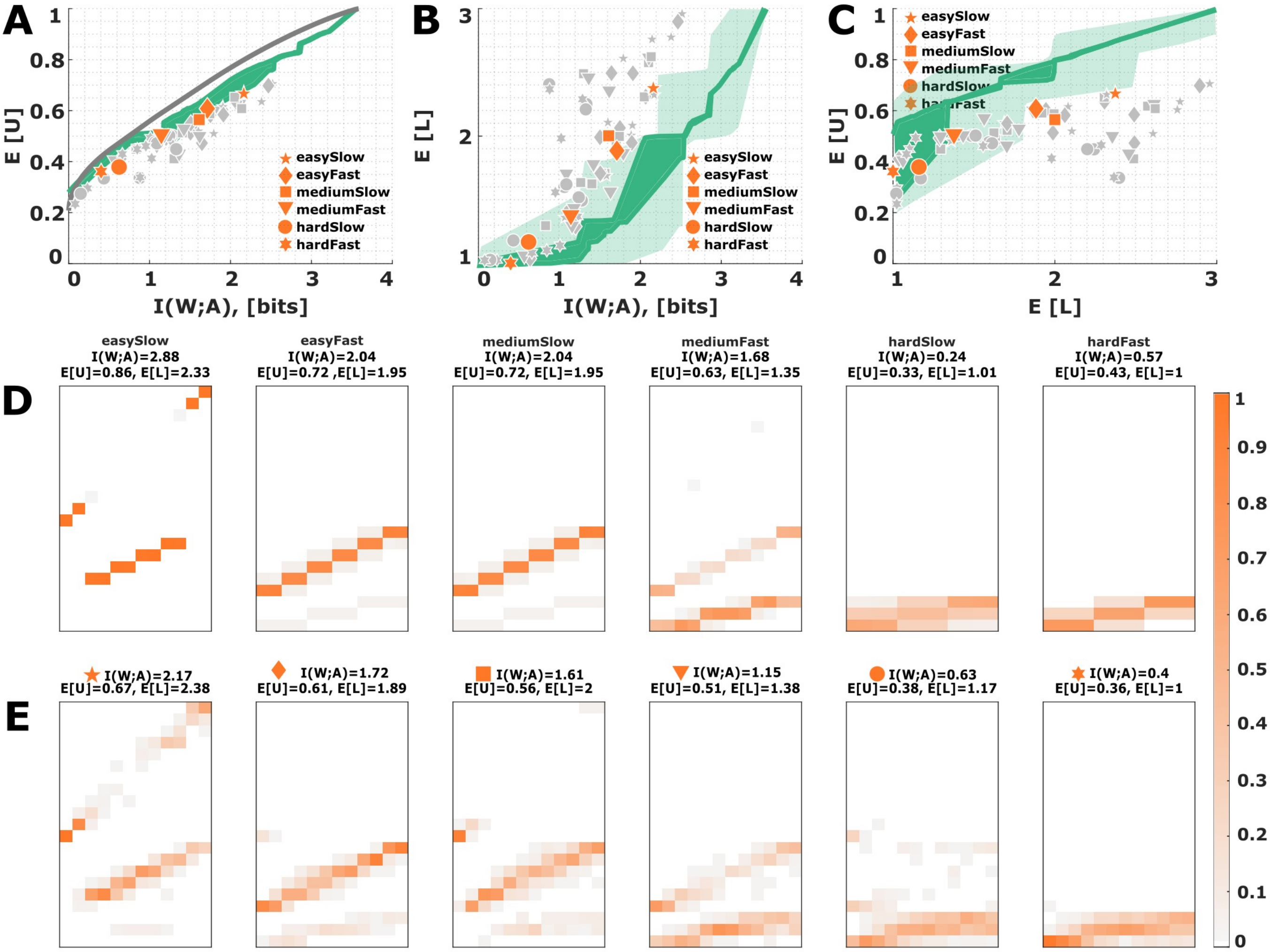

Figure S6: **Process-dependent noise: Gaussian responses with hierarchical decision-making.** (A) Assuming Gaussian decision strategies according to Equation (17) with varying standard deviation imposes a new efficiency frontier (solid green line) that lies below the bounded rational efficiency frontier (solid gray line). Each data point corresponds to a subject in a particular condition. The six emphasized data points correspond to subject S09 for who the stimulus-response distribution is shown below. (B,C) Level selection. The solid line in panel B shows the mean level selected by the Gaussian strategy profile with a given stimulus-response mutual information. The solid line in panel C shows the average utility achieved in each condition depending on the mean level selected. Each data point corresponds to a subject in a particular condition, with the same subject S09 emphasized. The shaded region indicates the confidence intervals given by the directed standard deviations (10). (D) Theoretical stimulus-response distributions for different points along the efficiency frontier closest to the data points of subject S09. (E) Stimulus-response pattern of subject S09 for comparison against the theoretical distributions.The fits of the remaining subjects can be seen in Figures S8H- S17H.

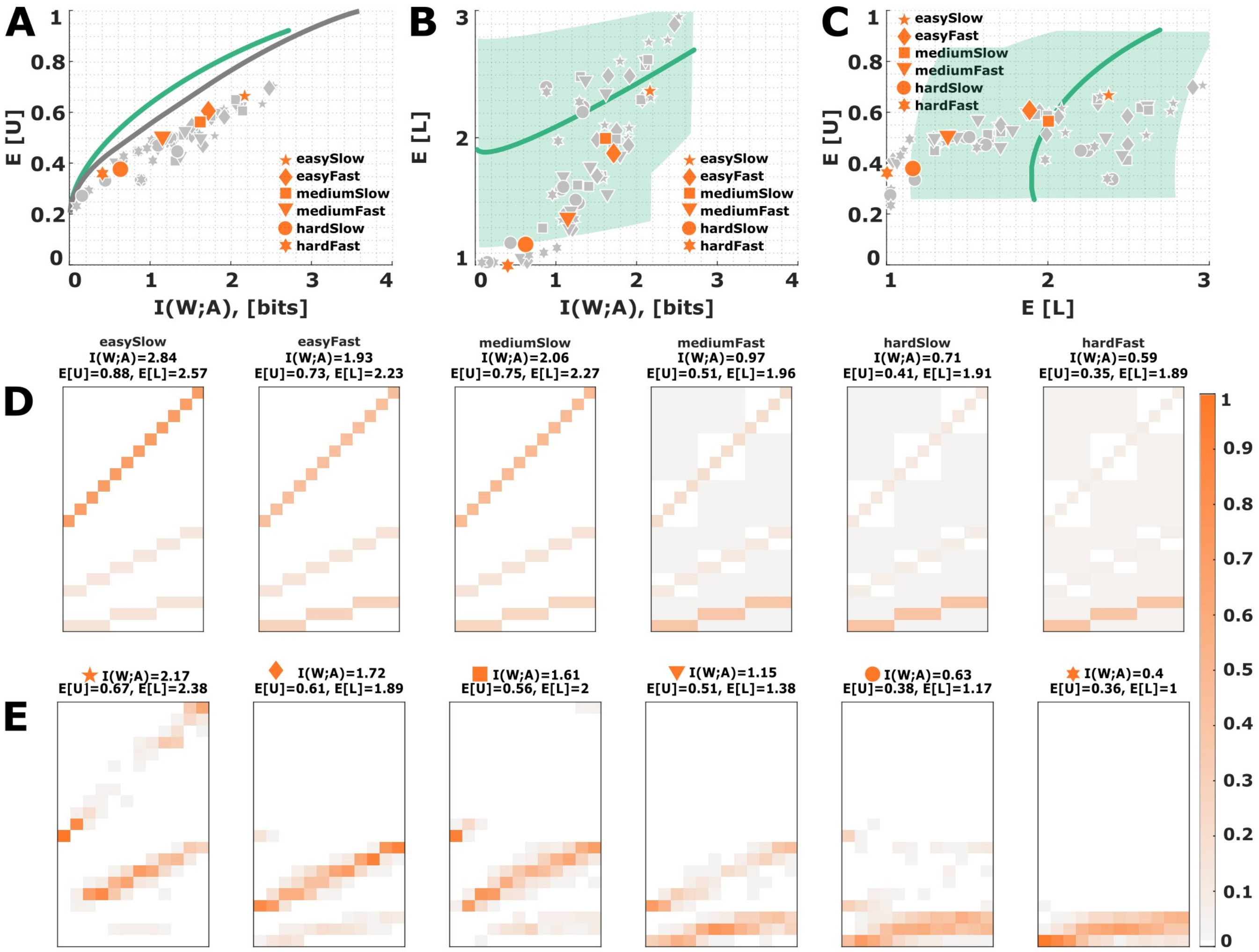

Figure S7: **Alternative information cost.** (A) Efficiency frontier with information costs for equal sized stimulus and action spaces. Each data point corresponds to a subject in a particular condition. The six emphasized data points correspond to subject S09 for who the stimulus-response distribution is shown below. (B,C) Level selection. The solid line in panel B shows the mean level selected under a given stimulus-response mutual information. The solid line in panel C shows the utility that it is expected to be obtained in each level. Each data point corresponds to a subject in a particular condition, with the same subject S09 emphasized. The shaded region indicates the signed standard deviation. (D) Theoretical stimulus-response distributions for different points along the efficiency frontier closest to the data points of subject S09. (E) Stimulus-response pattern of subject S09 for comparison against the theoretical distributions.

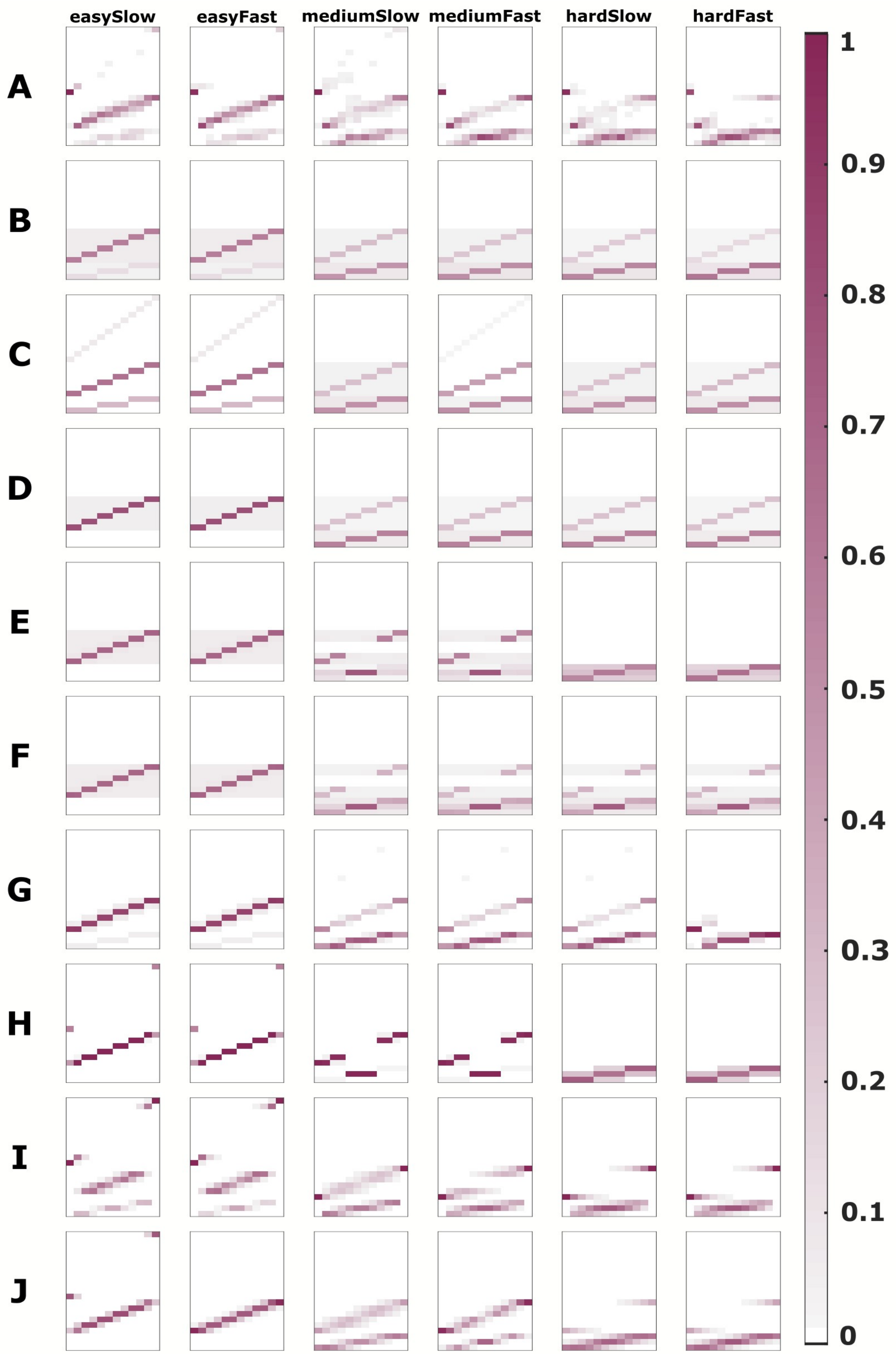

Figure S8: **Posterior model fits S01.** (A) Subjects' response distribution as measured in the experiment. (B) Bounded rational posterior fit with optimal prior. (C) Bounded rational posterior fit with fixed prior from equation (15). (D) Distorted utility posterior fit. (E) Posterior fit of subjective utility model with Gaussian neighborhood. (F) Posterior fit of subjective utility model with exponential neighborhood. (G) Posterior fit of the Gaussian response model. (H) Posterior fit of the Hierarchical Gaussian response model. (I) Posterior fit of binomial model. (J) Posterior fit of Thurstonian model.

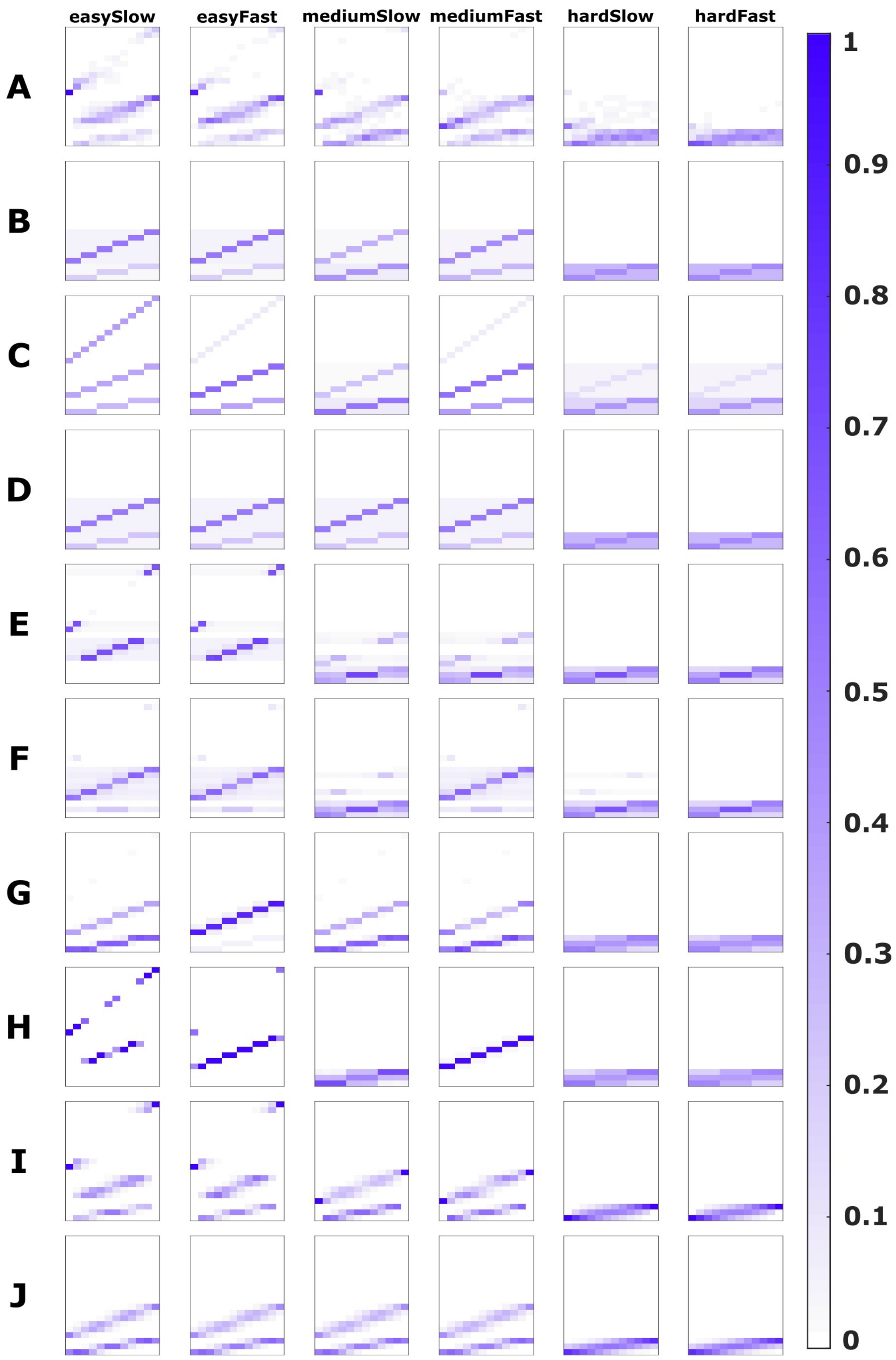

Figure S9: **Posterior model fits S02.** (A) Subjects' response distribution as measured in the experiment. (B) Bounded rational posterior fit with optimal prior. (C) Bounded rational posterior fit with fixed prior from equation (15). (D) Distorted utility posterior fit. (E) Posterior fit of subjective utility model with Gaussian neighborhood. (F) Posterior fit of subjective utility model with exponential neighborhood. (G) Posterior fit of the Gaussian response model. (H) Posterior fit of the Hierarchical Gaussian response model. (I) Posterior fit of binomial model. (J) Posterior fit of Thurstonian model.

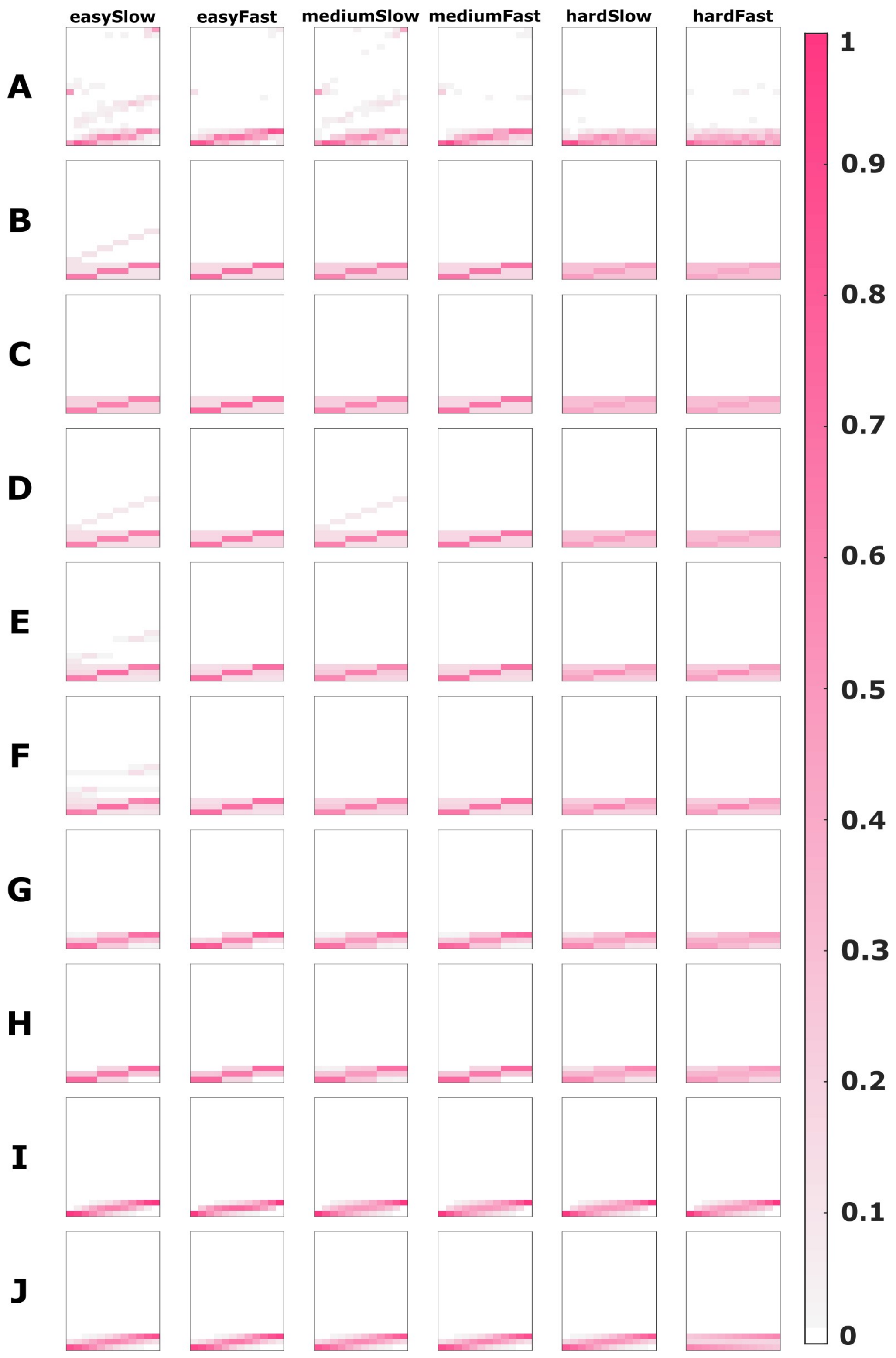

Figure S10: **Posterior model fits S03.** (A) Subjects' response distribution as measured in the experiment. (B) Bounded rational posterior fit with optimal prior. (C) Bounded rational posterior fit with fixed prior from equation (15). (D) Distorted utility posterior fit. (E) Posterior fit of subjective utility model with Gaussian neighborhood. (F) Posterior fit of subjective utility model with exponential neighborhood. (G) Posterior fit of the Gaussian response model. (H) Posterior fit of the Hierarchical Gaussian response model. (I) Posterior fit of binomial model. (J) Posterior fit of Thurstonian model.

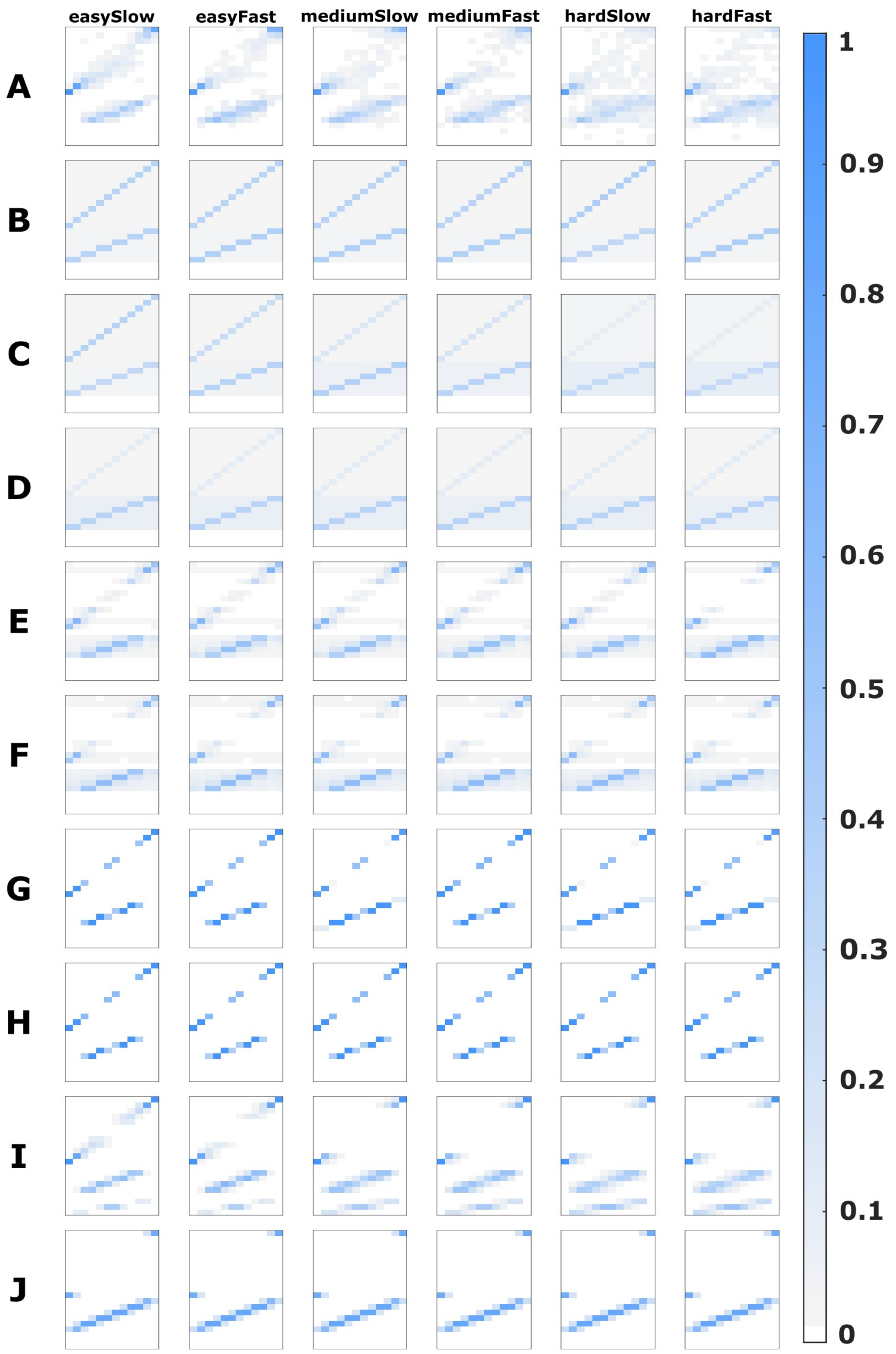

Figure S11: **Posterior model fits S04.** (A) Subjects' response distribution as measured in the experiment. (B) Bounded rational posterior fit with optimal prior. (C) Bounded rational posterior fit with fixed prior from equation (15). (D) Distorted utility posterior fit. (E) Posterior fit of subjective utility model with Gaussian neighborhood. (F) Posterior fit of subjective utility model with exponential neighborhood. (G) Posterior fit of the Gaussian response model. (H) Posterior fit of the Hierarchical Gaussian response model. (I) Posterior fit of binomial model. (J) Posterior fit of Thurstonian model.

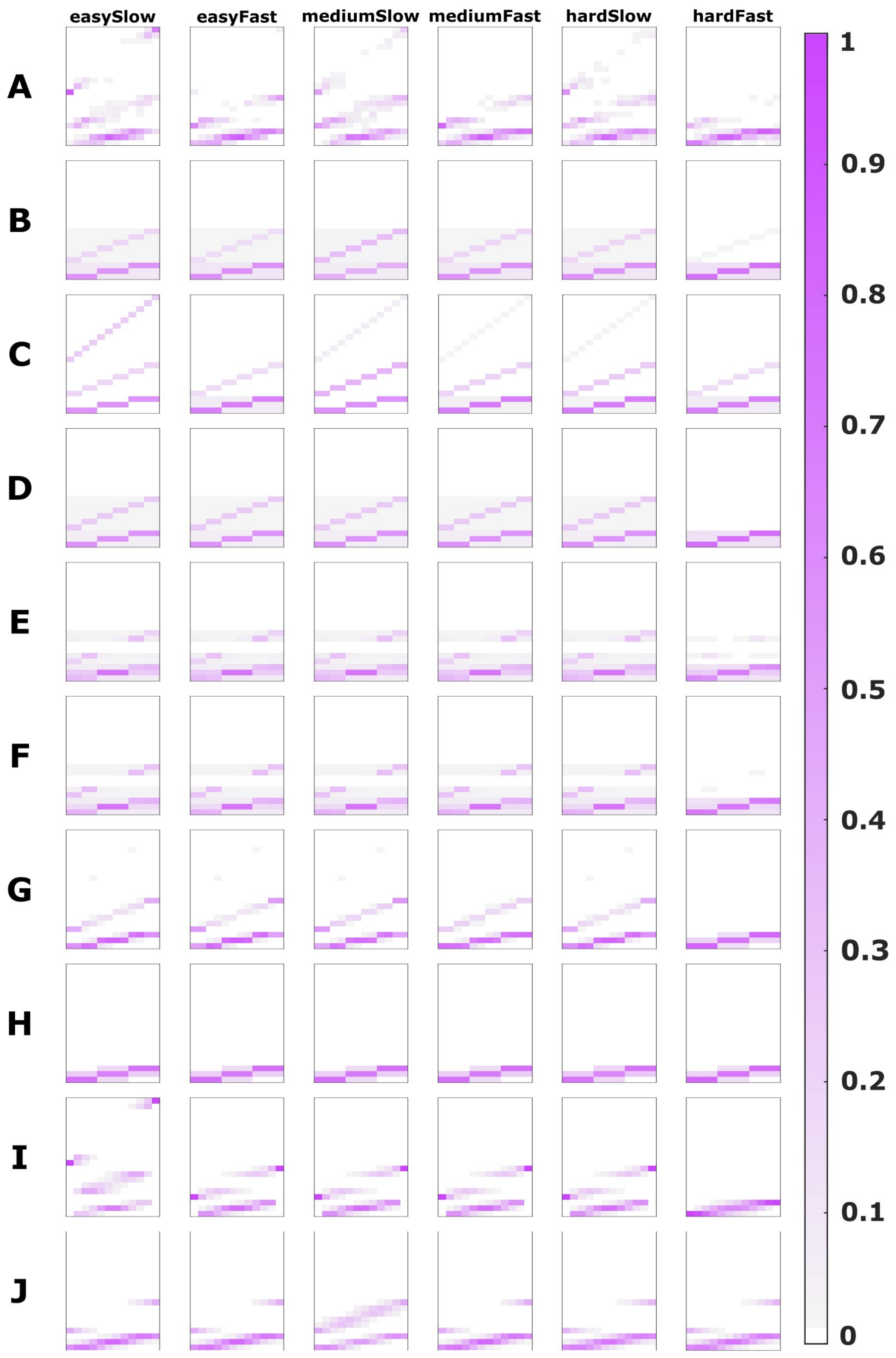

Figure S12: **Posterior model fits S05.** (A) Subjects' response distribution as measured in the experiment. (B) Bounded rational posterior fit with optimal prior. (C) Bounded rational posterior fit with fixed prior from equation (15). (D) Distorted utility posterior fit. (E) Posterior fit of subjective utility model with Gaussian neighborhood. (F) Posterior fit of subjective utility model with exponential neighborhood. (G) Posterior fit of the Gaussian response model. (H) Posterior fit of the Hierarchical Gaussian response model. (I) Posterior fit of binomial model. (J) Posterior fit of Thurstonian model.

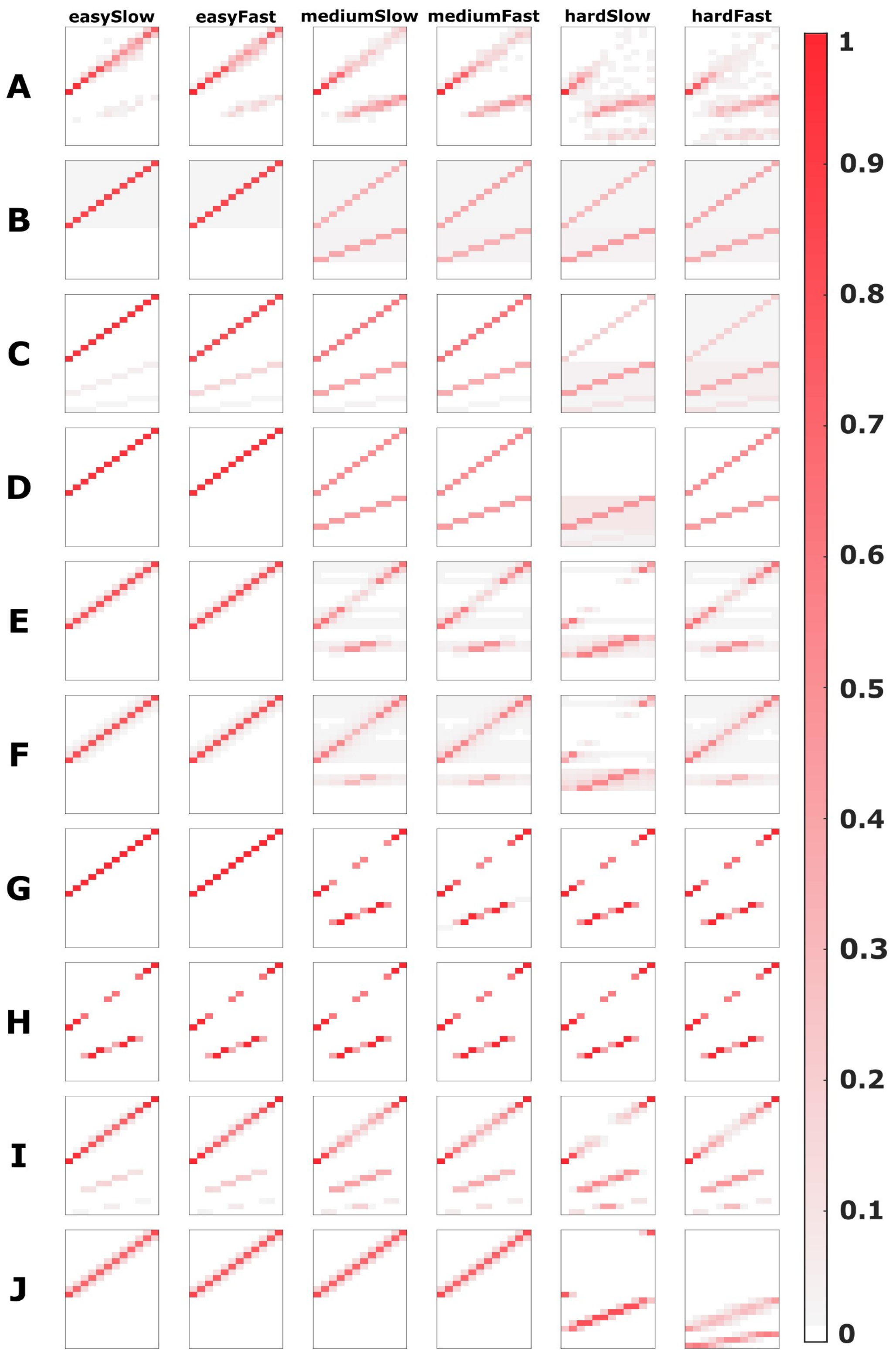

Figure S13: **Posterior model fits S06.** (A) Subjects' response distribution as measured in the experiment. (B) Bounded rational posterior fit with optimal prior. (C) Bounded rational posterior fit with fixed prior from equation (15). (D) Distorted utility posterior fit. (E) Posterior fit of subjective utility model with Gaussian neighborhood. (F) Posterior fit of subjective utility model with exponential neighborhood. (G) Posterior fit of the Gaussian response model. (H) Posterior fit of the Hierarchical Gaussian response model. (I) Posterior fit of binomial model. (J) Posterior fit of Thurstonian model.

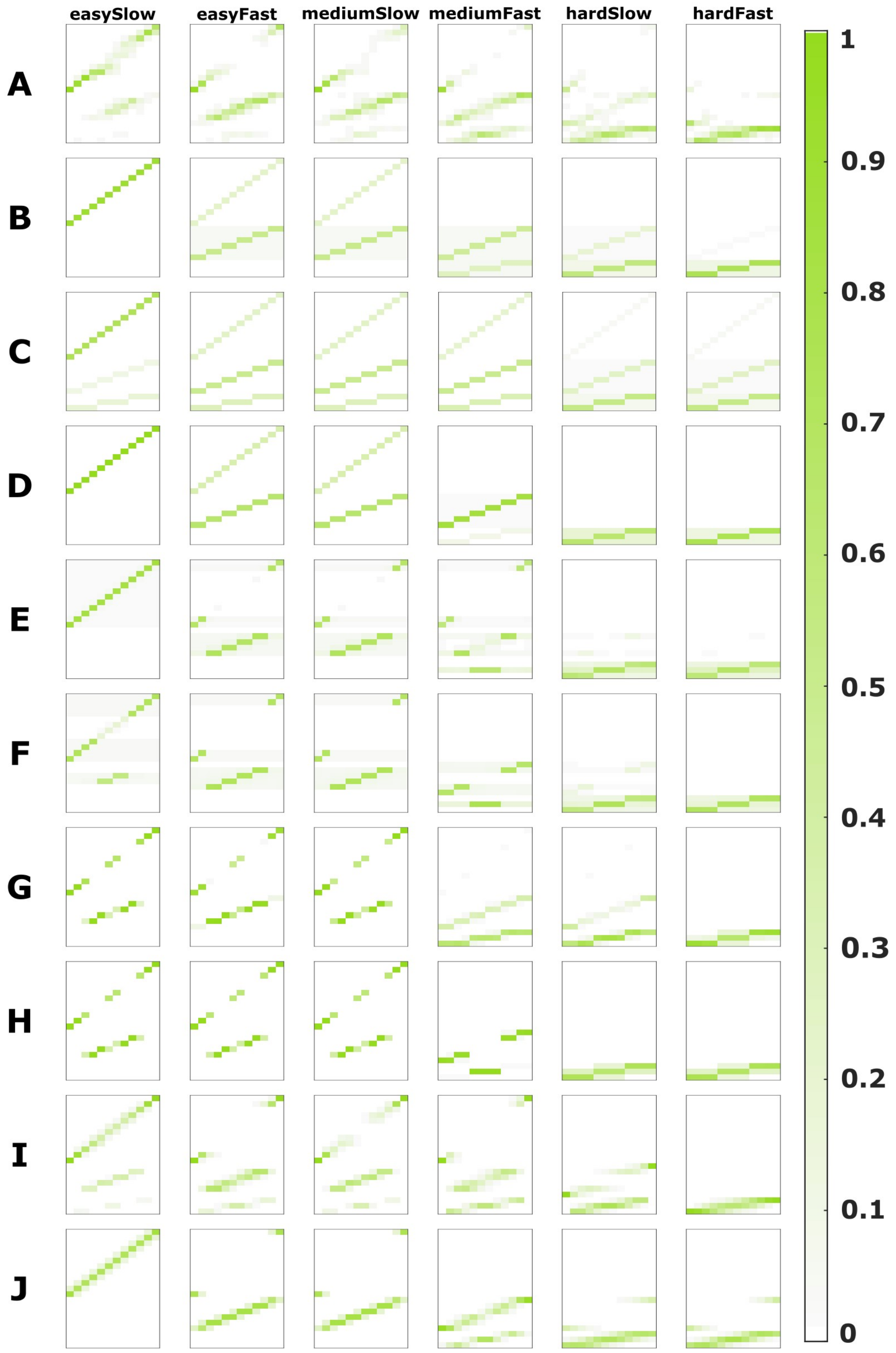

Figure S14: **Posterior model fits S07.** (A) Subjects' response distribution as measured in the experiment. (B) Bounded rational posterior fit with optimal prior. (C) Bounded rational posterior fit with fixed prior from equation (15). (D) Distorted utility posterior fit. (E) Posterior fit of subjective utility model with Gaussian neighborhood. (F) Posterior fit of subjective utility model with exponential neighborhood. (G) Posterior fit of the Gaussian response model. (H) Posterior fit of the Hierarchical Gaussian response model. (I) Posterior fit of binomial model. (J) Posterior fit of Thurstonian model.

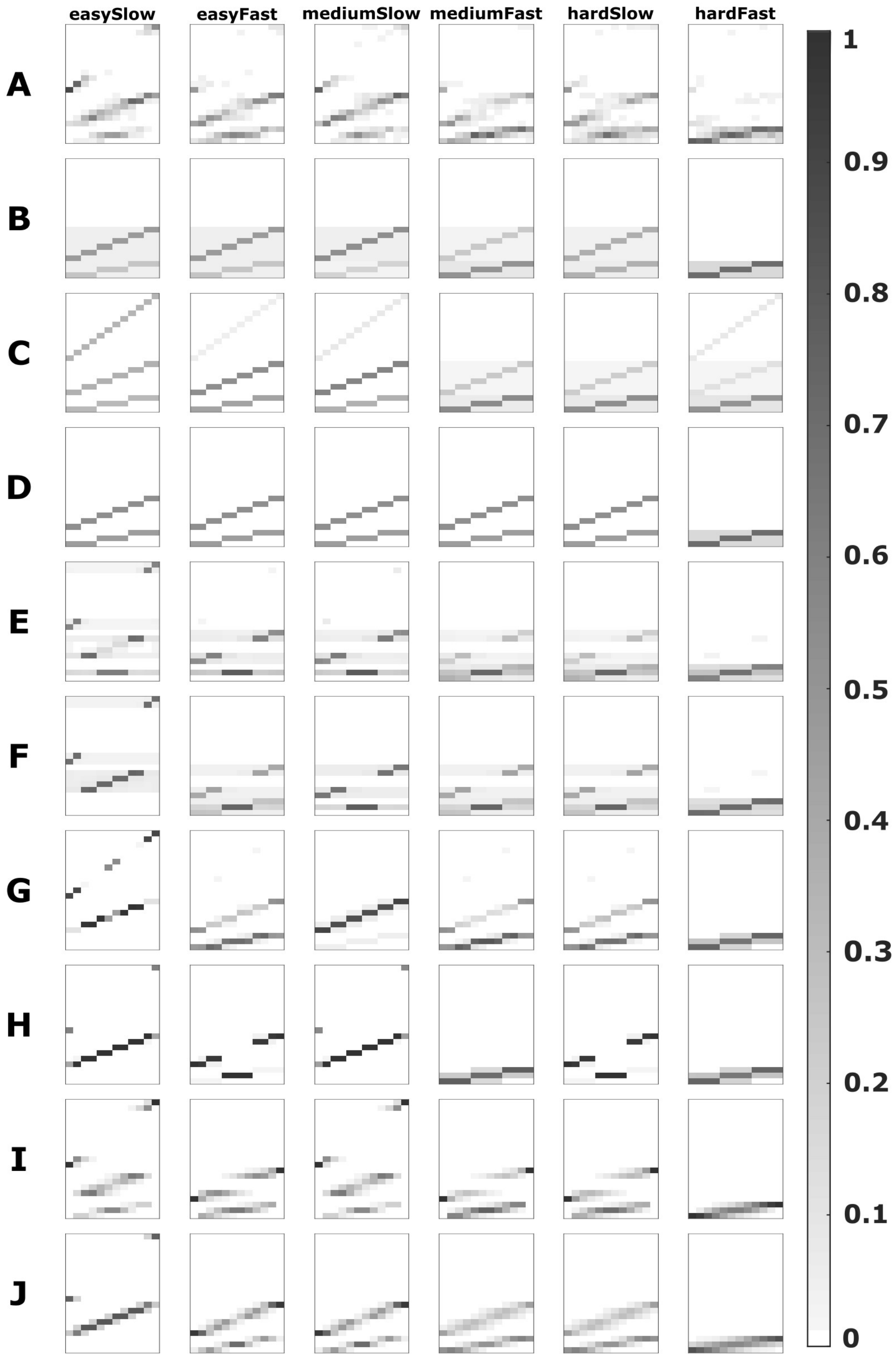

Figure S15: **Posterior model fits S08.** (A) Subjects' response distribution as measured in the experiment. (B) Bounded rational posterior fit with optimal prior. (C) Bounded rational posterior fit with fixed prior from equation (15). (D) Distorted utility posterior fit. (E) Posterior fit of subjective utility model with Gaussian neighborhood. (F) Posterior fit of subjective utility model with exponential neighborhood. (G) Posterior fit of the Gaussian response model. (H) Posterior fit of the Hierarchical Gaussian response model. (I) Posterior fit of binomial model. (J) Posterior fit of Thurstonian model.

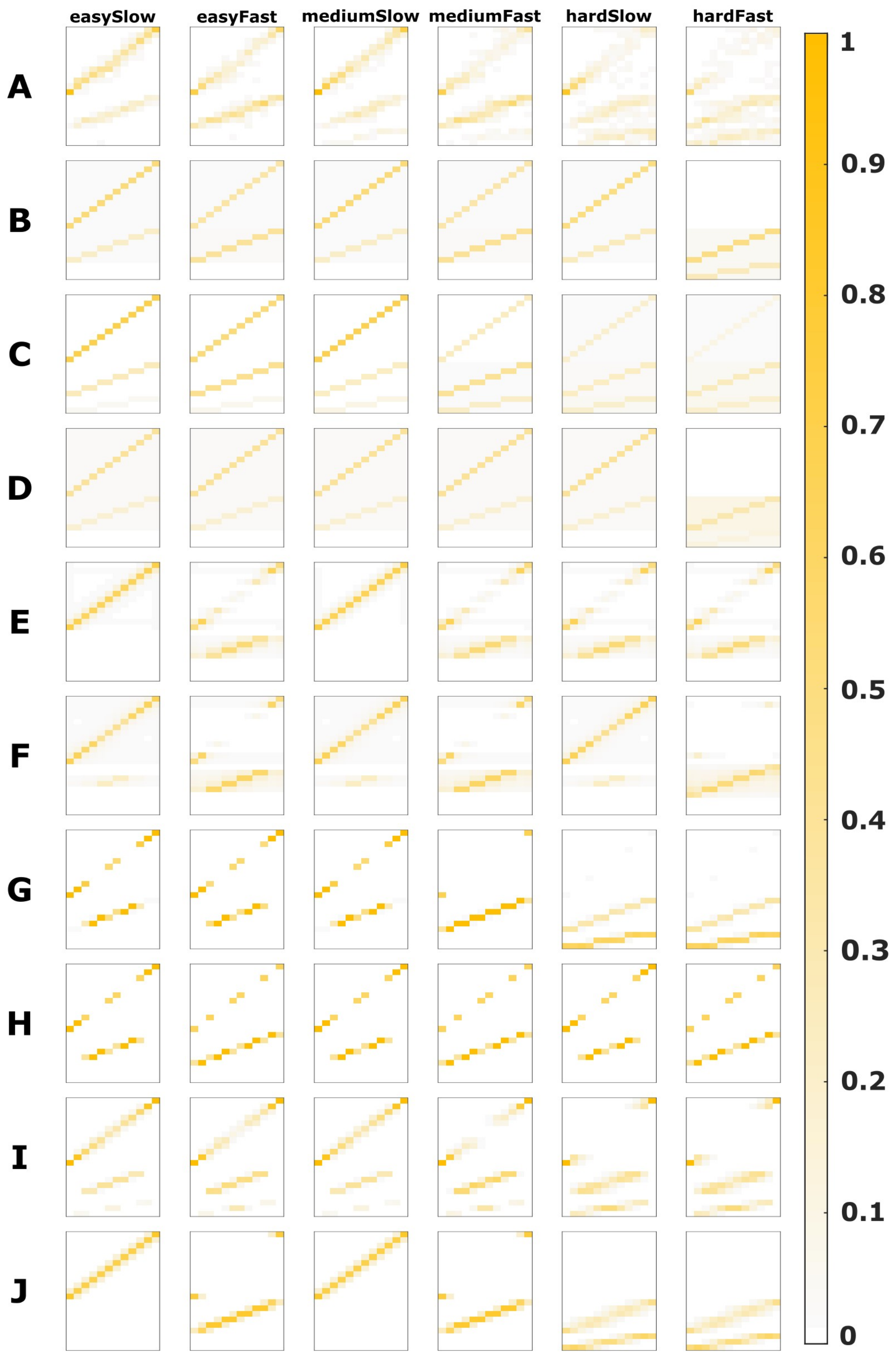

Figure S16: **Posterior model fits S10.** (A) Subjects' response distribution as measured in the experiment. (B) Bounded rational posterior fit with optimal prior. (C) Bounded rational posterior fit with fixed prior from equation (15). (D) Distorted utility posterior fit. (E) Posterior fit of subjective utility model with Gaussian neighborhood. (F) Posterior fit of subjective utility model with exponential neighborhood. (G) Posterior fit of the Gaussian response model. (H) Posterior fit of the Hierarchical Gaussian response model. (I) Posterior fit of binomial model. (J) Posterior fit of Thurstonian model.

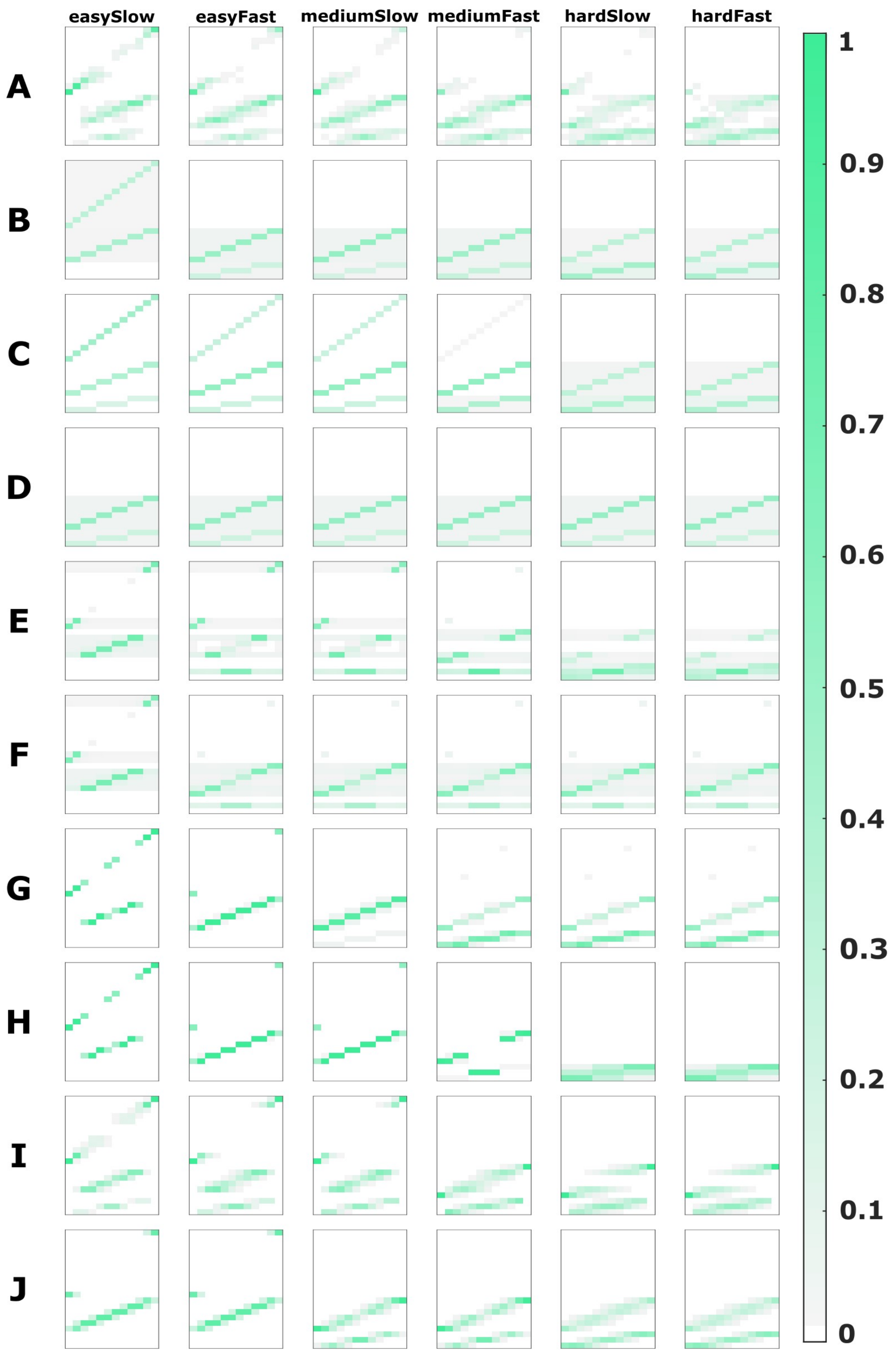

Figure S17: **Posterior model fits S11.** (A) Subjects' response distribution as measured in the experiment. (B) Bounded rational posterior fit with optimal prior. (C) Bounded rational posterior fit with fixed prior from equation (15). (D) Distorted utility posterior fit. (E) Posterior fit of subjective utility model with Gaussian neighborhood. (F) Posterior fit of subjective utility model with exponential neighborhood. (G) Posterior fit of the Gaussian response model. (H) Posterior fit of the Hierarchical Gaussian response model. (I) Posterior fit of binomial model. (J) Posterior fit of Thurstonian model.

Table S2. Grid search ranges for the different parameters

Model	Range
BR-optPrior	$\beta = [0.5 - 6], \Delta\beta = 0.11 \times 10^{-3}$
BR-fixedPrior$_1$	$\beta = [0.5 - 6], \Delta\beta = 0.11 \times 10^{-3}$
BR-fixedPrior$_2$	$\beta = [0.5 - 6], \Delta\beta = 0.11 \times 10^{-3}$
BR-fixedPrior$_3$	$q_1 = [0 - 1], \Delta q_1 = 0.01$<br>$q_2 = [0 - 1], \Delta q_2 = 0.01$<br>$q_3 = [0 - 1], \Delta q_3 = 0.01$<br>$\beta = [0.5 - 10], \Delta\beta = 0.0019$
Distorted utility	$U_1 = [0 - 1], \Delta U_1 = 0.025$<br>$U_2 = [0 - 1], \Delta U_2 = 0.025$<br>$U_3 = [0 - 1], \Delta U_3 = 0.025$<br>$\beta = [0.5 - 100], \Delta\beta = 0.0996$
expUtility	$\theta = [0.1 - 10], \Delta\theta = 0.0099$<br>$\beta = [0.5 - 50], \Delta\beta = 0.0495$
gaussUtility	$\theta = [0.001 - 1], \Delta\theta = 0.0099$<br>$\beta = [0.5 - 50], \Delta\beta = 0.0495$
gaussResponse-BR	$\theta = [0.001 - 1], \Delta\theta = 0.0099$<br>$\beta = [0.1 - 10], \Delta\beta = 0.001$
Thurstonian	$\sigma = [0.01 - 1], \Delta\sigma = 1.98 \times 10^{-5}$
Binomial	$T = [1 - 700], \Delta T = 1$
gaussResponseHier	$\sigma = [0.001 - 1], \Delta\sigma = 0.001$<br>$\beta_1 = [0.1 - 10], \Delta\beta_1 = 0.1$<br>$\beta_2 = [0.1 - 10], \Delta\beta_2 = 0.001$
Inf-12x12	$\beta = [0.1 - 10], \Delta\beta = 0.001$

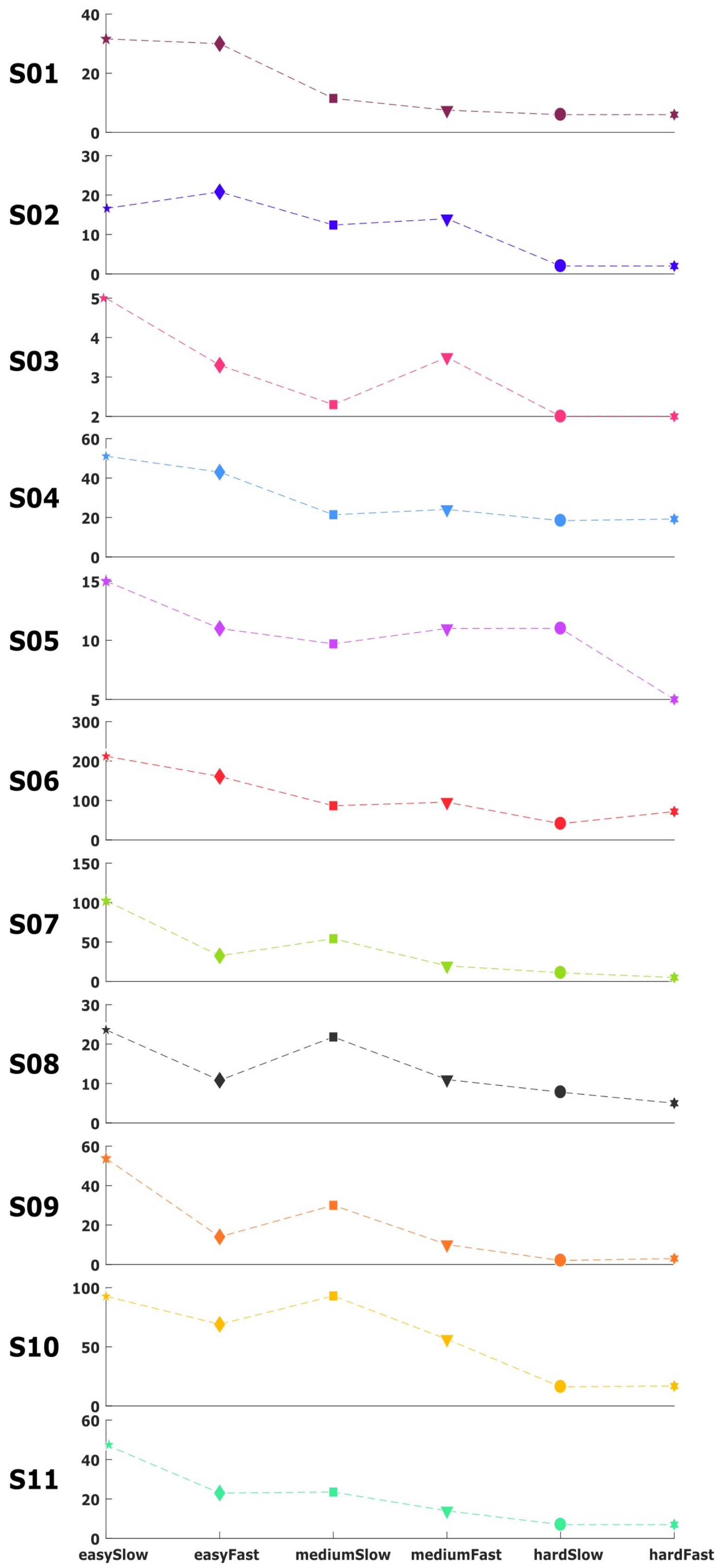

Figure S18: **Binomial model.** Best fitting parameter values for θ for all subjects and conditions. As expected, the parameter θ decreases when the conditions are more difficult.

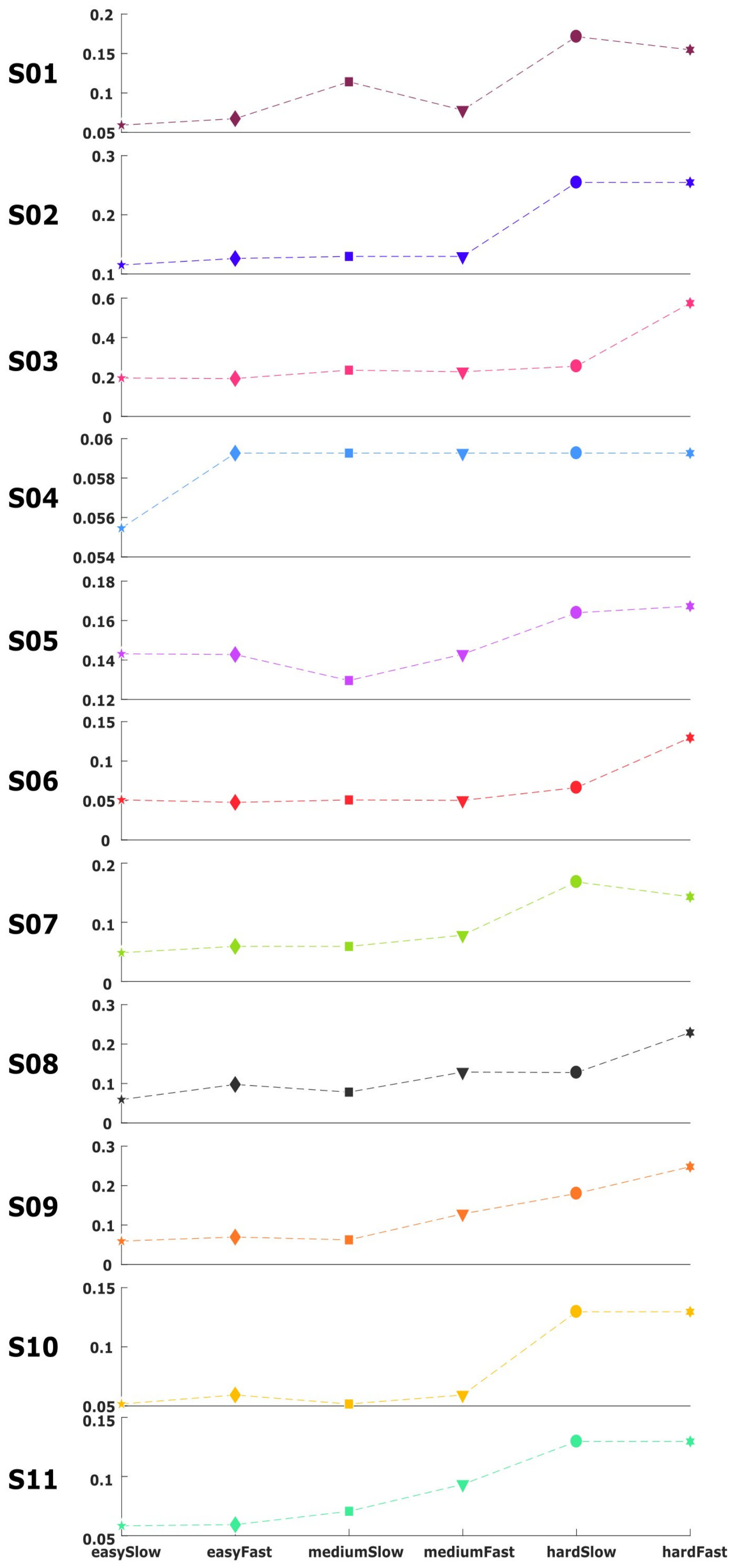

Figure S19: **Thurstonian model.** Best fitting parameter values for σ for all subjects and conditions. As expected, the parameter σ increases when the conditions are more difficult.

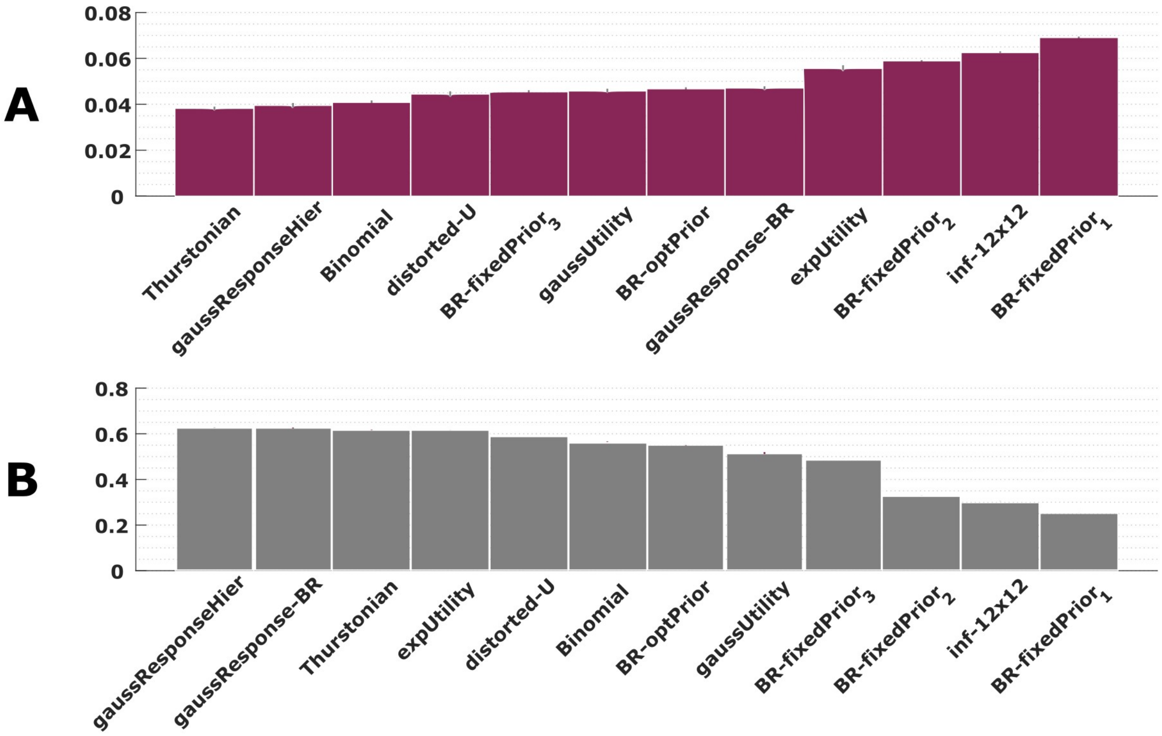

Figure S20: **Model cross validation for subject S01.** (A) Mean absolute error between the subject's response frequencies and predicted choice probabilities. Error bars indicate standard error across folds. Lower values indicate better predictions. (B) Average predicted probability of subject's choice of abstraction level. Higher values indicate better predictions.

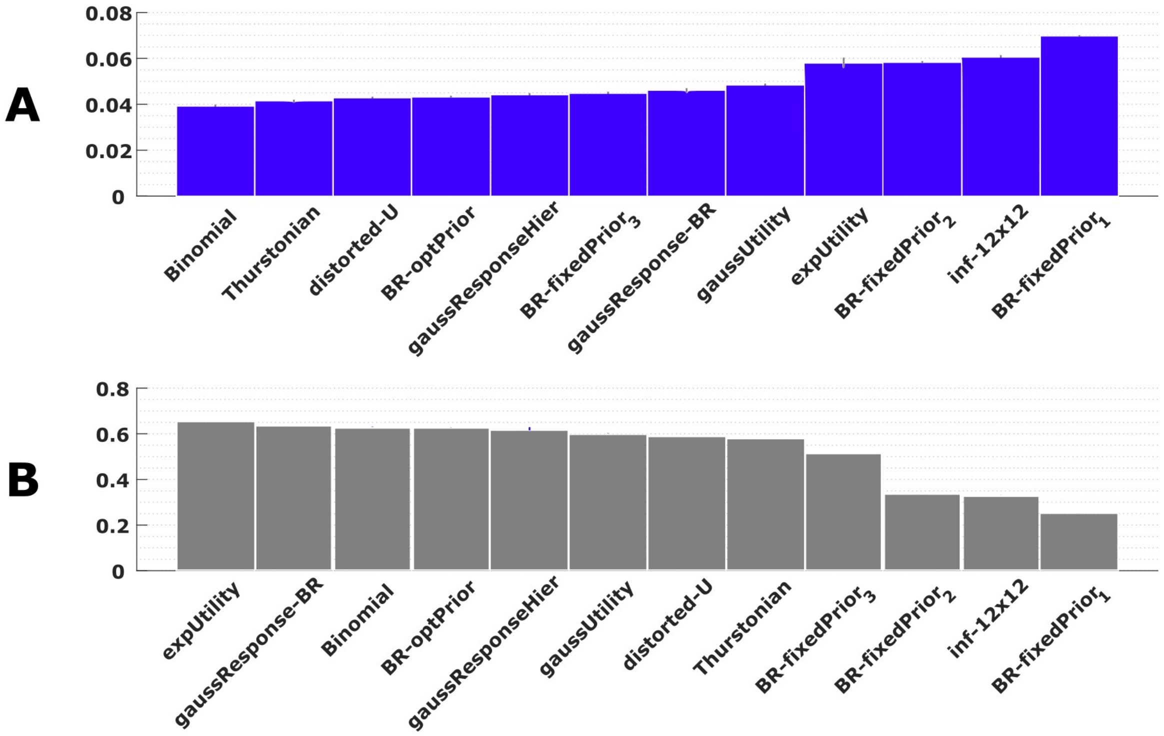

Figure S21: **Model cross validation for subject S02.** (A) Mean absolute error between the subject's response frequencies and predicted choice probabilities. Error bars indicate standard error across folds. Lower values indicate better predictions. (B) Average predicted probability of subject's choice of abstraction level. Higher values indicate better predictions.

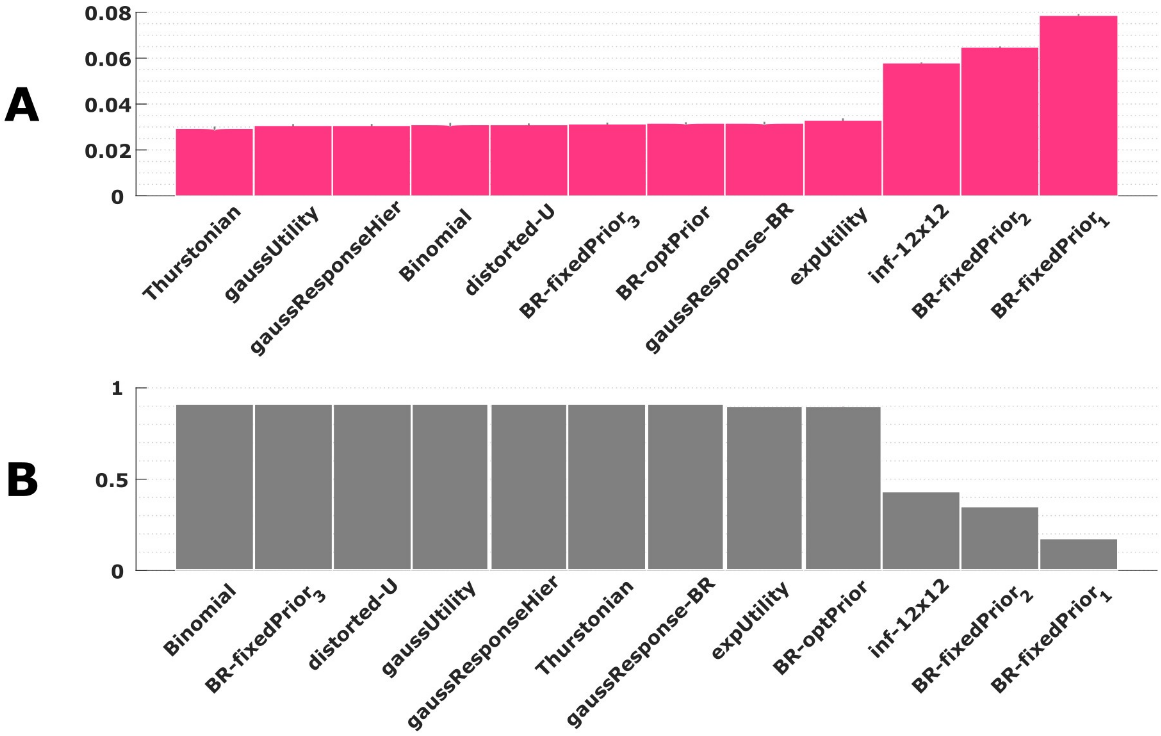

Figure S22: **Model cross validation for subject S03.** (A) Mean absolute error between the subject's response frequencies and predicted choice probabilities. Error bars indicate standard error across folds. Lower values indicate better predictions. (B) Average predicted probability of subject's choice of abstraction level. Higher values indicate better predictions.

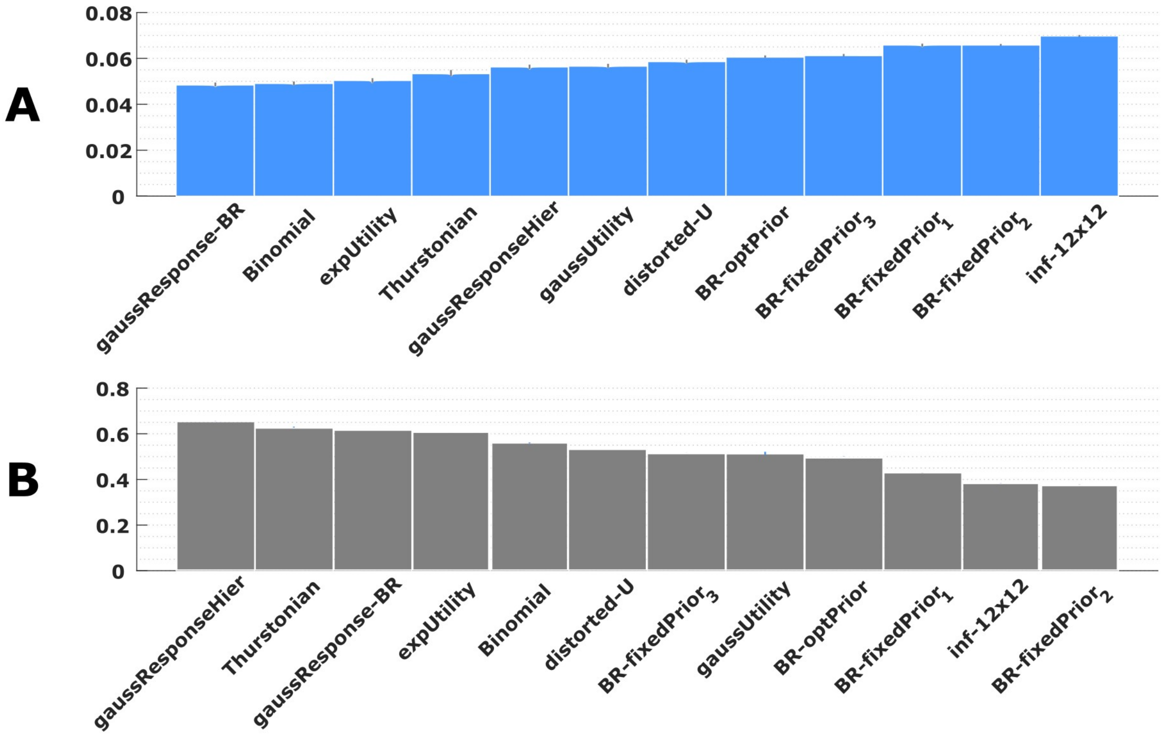

Figure S23: **Model cross validation for subject S04.** (A) Mean absolute error between the subject's response frequencies and predicted choice probabilities. Error bars indicate standard error across folds. Lower values indicate better predictions. (B) Average predicted probability of subject's choice of abstraction level. Higher values indicate better predictions.

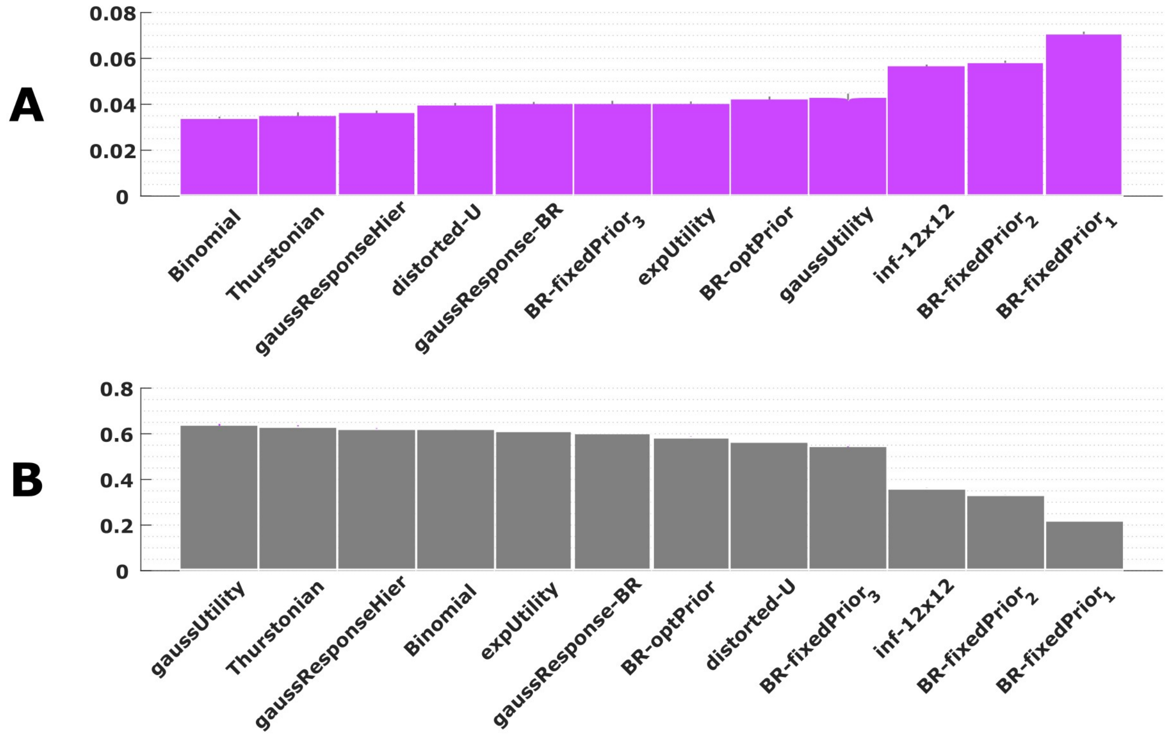

Figure S24: **Model cross validation for subject S05.** (A) Mean absolute error between the subject's response frequencies and predicted choice probabilities. Error bars indicate standard error across folds. Lower values indicate better predictions. (B) Average predicted probability of subject's choice of abstraction level. Higher values indicate better predictions.

A

B

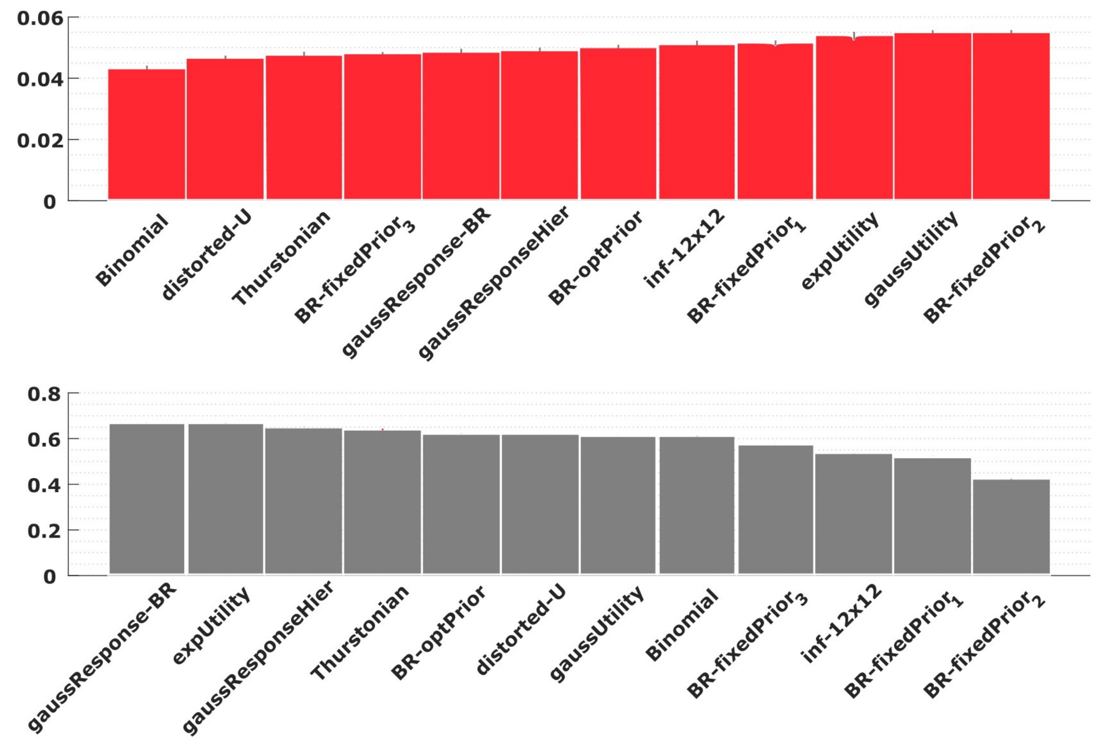

Figure S25: **Model cross validation for subject S06.** (A) Mean absolute error between subjects' response frequencies and predicted choice probabilities. Error bars indicate standard error across subjects. Lower values indicate better predictions. (B) Average predicted probability of subjects choice of abstraction level. Higher values indicate better predictions.

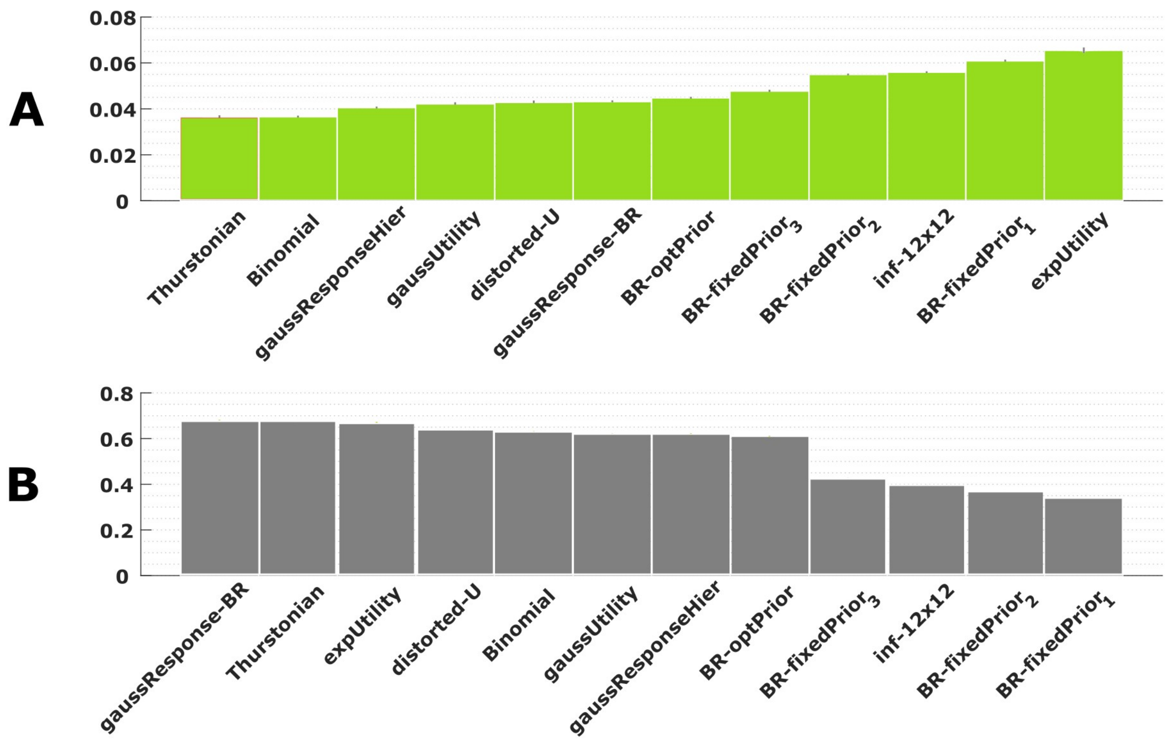

Figure S26: **Model cross validation for subject S07.** (A) Mean absolute error between the subject's response frequencies and predicted choice probabilities. Error bars indicate standard error across folds. Lower values indicate better predictions. (B) Average predicted probability of subject's choice of abstraction level. Higher values indicate better predictions.

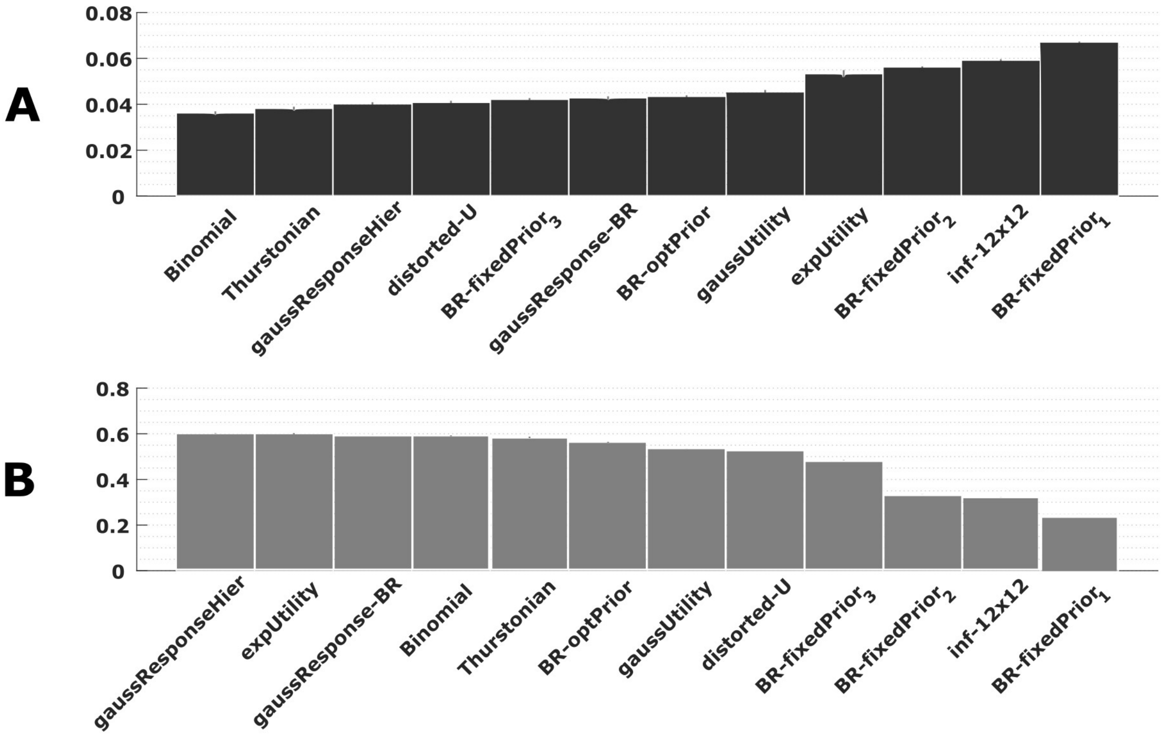

Figure S27: **Model cross validation for subject S08.** (A) Mean absolute error between the subject's response frequencies and predicted choice probabilities. Error bars indicate standard error across folds. Lower values indicate better predictions. (B) Average predicted probability of subject's choice of abstraction level. Higher values indicate better predictions.

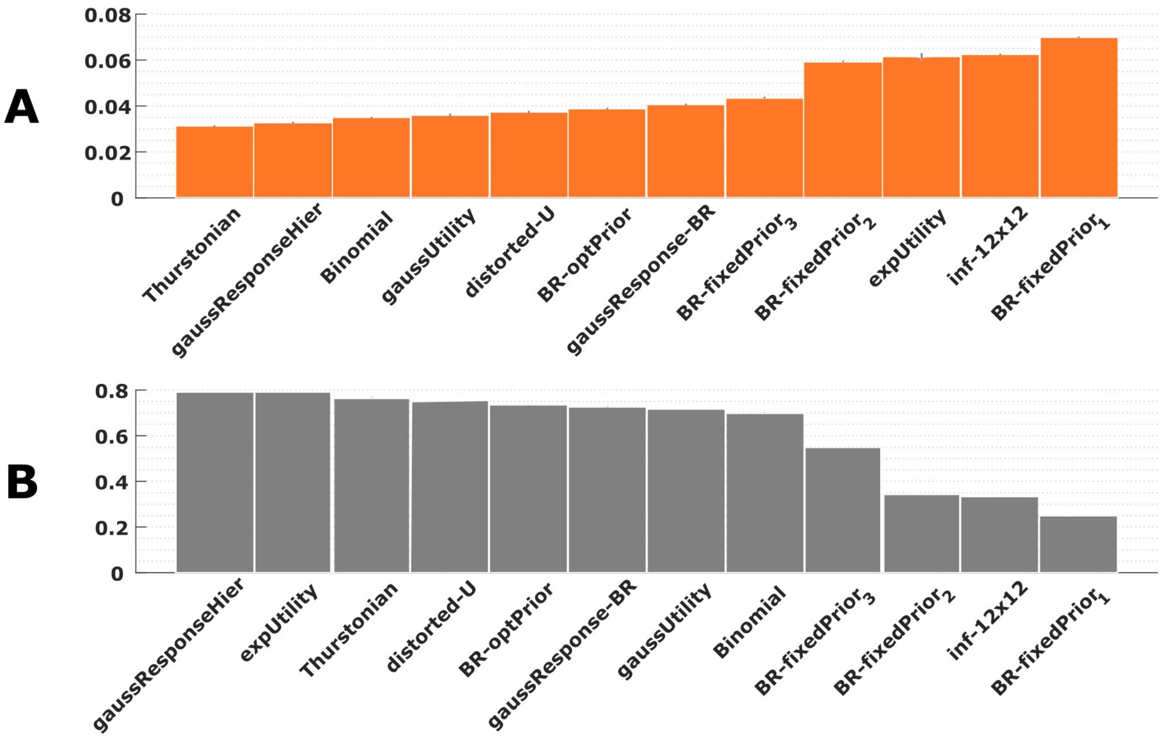

Figure S28: **Model cross validation for subject S09.** (A) Mean absolute error between the subject's response frequencies and predicted choice probabilities. Error bars indicate standard error across folds. Lower values indicate better predictions. (B) Average predicted probability of subject's choice of abstraction level. Higher values indicate better predictions.

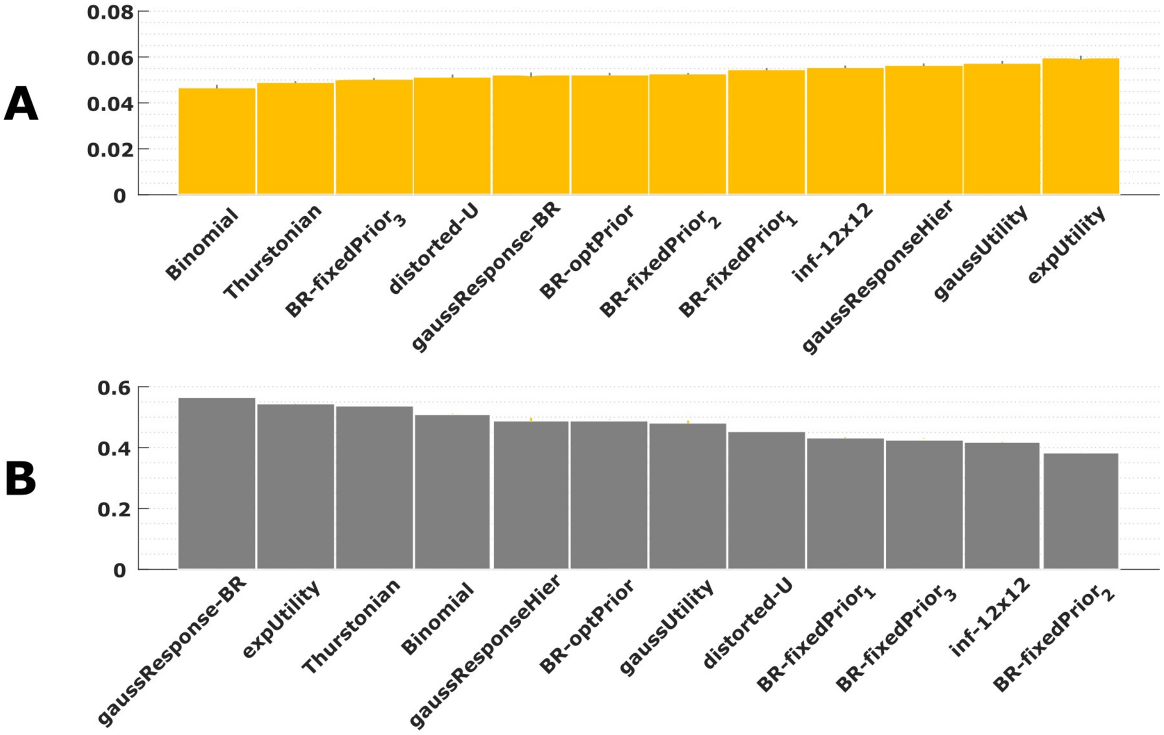

Figure S29: **Model cross validation for subject S10.** (A) Mean absolute error between the subject's response frequencies and predicted choice probabilities. Error bars indicate standard error across folds. Lower values indicate better predictions. (B) Average predicted probability of subject's choice of abstraction level. Higher values indicate better predictions.

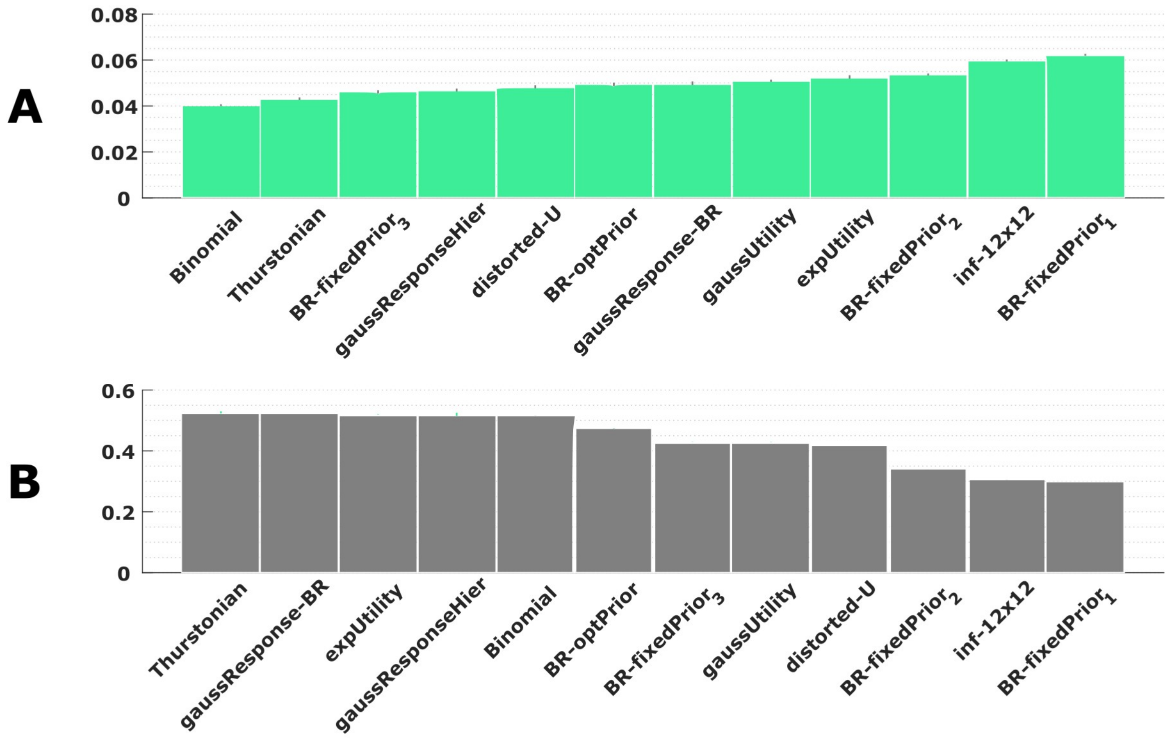

Figure S30: **Model cross validation for subject S11.** (A) Mean absolute error between the subject's response frequencies and predicted choice probabilities. Error bars indicate standard error across folds. Lower values indicate better predictions. (B) Average predicted probability of subject's choice of abstraction level. Higher values indicate better predictions.
